# How *Salmonella* Works Under Osmotic and Desiccation Stresses

**DOI:** 10.1111/1541-4337.70439

**Published:** 2026-03-29

**Authors:** Mayara Messias Oliveira, Dionisio P. Amorim‐Neto, Anderson S. Sant'Ana

**Affiliations:** ^1^ Department of Food Science and Nutrition, Faculty of Food Engineering University of Campinas Campinas São Paulo Brazil

**Keywords:** biofilm formation, cross‐protection, low‐moisture foods, stress adaptation, viable but non‐culturable state, virulence regulation

## Abstract

*Salmonella* remains one of the leading threats in foods with reduced water activity, where it can survive for long periods and cause outbreaks. Its persistence stems from a wide array of adaptive strategies shaped by the selective pressures imposed by low‐moisture foods. Under osmotic or desiccation stress, the bacterium quickly adjusts its gene expression, activates DNA repair systems, and alters its growth dynamics, often forming elongated cells that conserve energy. At the same time, it reshapes its membranes, accumulates protective molecules such as trehalose, proline, and betaine, and frequently organizes into biofilms that shield cells from harsh conditions. These protective responses are closely tied to its disease‐causing potential, because stress signals also influence virulence factors, secretion systems, fimbriae, and flagella, ultimately making the pathogen more capable of establishing infection. Stress exposure can further trigger cross‐protection, enhancing resistance to heat, disinfectants, and other common barriers in food processing. In some cases, cells enter a dormant, viable but non‐culturable state, remaining undetectable until favorable conditions allow their revival. Together, these mechanisms reveal the extraordinary resilience of *Salmonella* in dry food environments and highlight the challenge of ensuring safety in products that are often ready to eat. A deeper understanding of these adaptations is essential to develop more effective control strategies and reduce the burden of salmonellosis worldwide. In this review, we provide a detailed discussion of the morphological, transcriptional, and metabolic aspects of *Salmonella*’s response to osmotic and desiccation stresses.

## 1 Introduction

Although water is essential for life and for many metabolic reactions, microorganisms are remarkably resilient and can survive in diverse stressful environments, including those with limited water availability (Lebre et al. 2017). When water is scarce, bacteria can face two major forms of water‐related stress: osmotic stress and desiccation stress.

**FIGURE 1:**
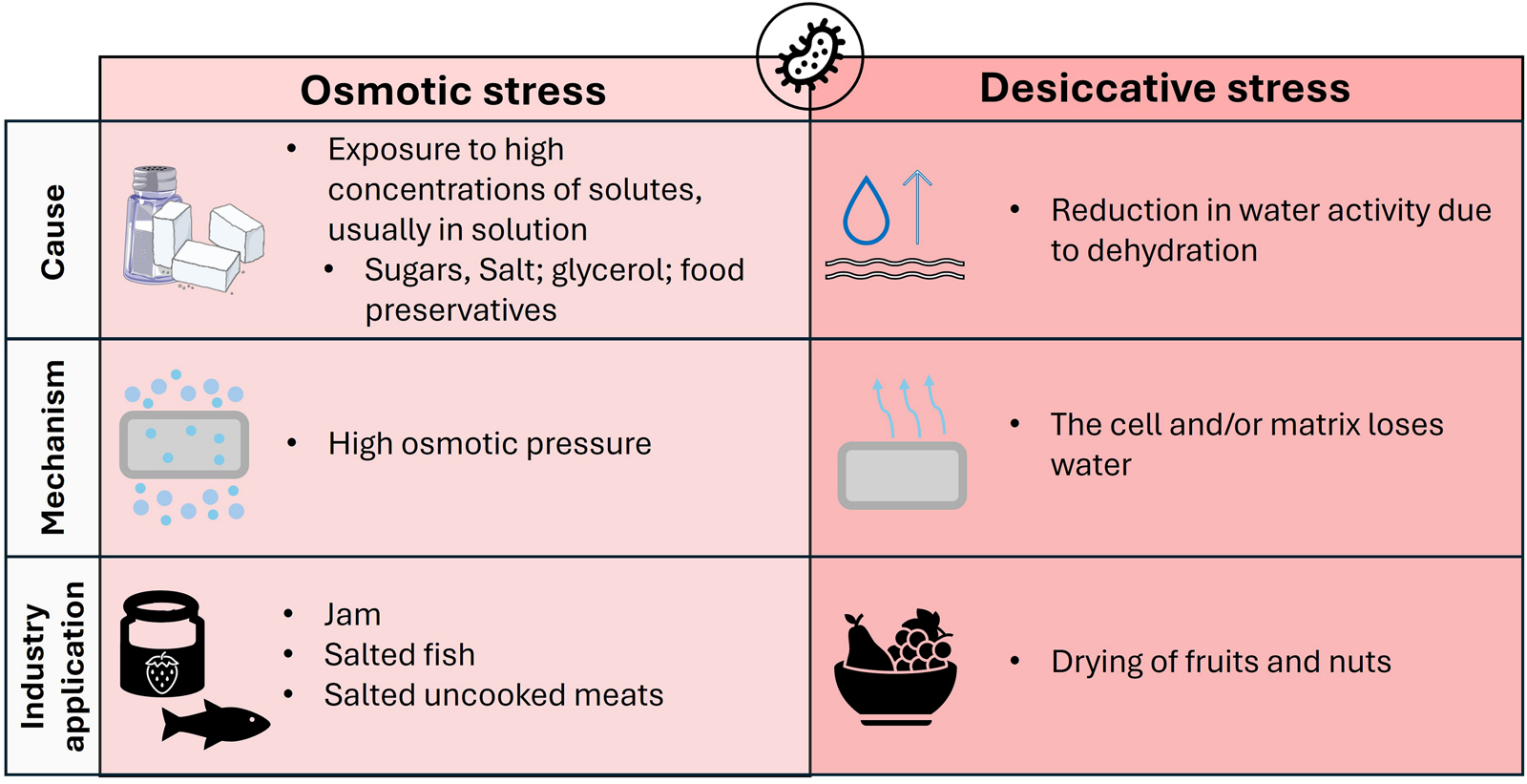
Main characteristics of osmotic and desiccative stress phenomena. Image credit: The figure was drawn by using modified pictures from Servier Medical Art. Servier Medical Art by Servier is licensed under a Creative Commons Attribution 3.0 Unported License <https://creativecommons.org/licenses/by/3.0/>.

Osmotic stress is characterized by the exposure of bacteria to high concentrations of solute, generally dispersed in a solution with a low water content, giving the cells high osmotic pressure (Lebre et al. 2017). It can be caused by different types of solutes, such as sugar (Jayeola et al. 2022), salt (Oliveira et al. 2021), preservatives (Quirós et al. 2009), and glycerol (Finn, Hinton, et al. 2013), among others. In the food industry, for example, this type of stress is applied to the production of fruit juices and some preserves, such as selected vegetables, syrup, jam, salted fish, or salted uncooked meats (e.g., ham) (Figure 1).

Desiccation stress, on the other hand, results from reduced water activity (aw), usually due to dehydration, where bacteria lose water from their cells or surrounding matrix to the atmosphere until reaching equilibrium with relative humidity or the desired water activity level. Industrial preservation processes, such as fruit and nut drying, explore this principle by lowering water activity to inhibit contaminants and prevent food‐borne pathogen growth (Alp and Bulantekin 2021) (Figure 1).

Pure water has an aw of 1.00, whereas foods are always measured below this value (Belitz and Grosch 2013) and are classified as high‐moisture (HMF, aw up to 0.90, e.g., fresh foods), intermediate‐moisture (IMF, aw 0.60–0.90, e.g., fermented meats, jellies, and canned foods), or low‐moisture (LMF, aw <0.60, e.g., dehydrated foods) (Barbosa‐Cánovas et al. 2003; Beuchat et al. 2013; Fernández‐Salguero et al. 1993; Jay et al. 2008).

This variable is important because microorganisms require optimal aw levels for growth, with most bacteria unable to divide at <0.91 aw and most fungi inactivate their metabolism at aw <0.7, though some survive in much drier environments (Lebre et al. 2017). *Salmonella* grows optimally at aw 0.99, with a minimum growth limit of aw 0.94 (Jay et al. 2008). In LMF, *Salmonella* populations show slow reduction rates; for instance, in chocolate, a reduction of 4.19 log_10_ CFU/g was observed over 12 months (Sun et al. 2023), whereas in *paçoca* and roasted peanuts, reductions of 2.53 and 3.82 log_10_ CFU/g were observed after 420 days (Nascimento et al. 2018). Similarly, in almond kernels, a 1.2‐log_10_ CFU/g reduction was measured after 70 weeks of storage (Limcharoenchat et al. 2019). Together, these data highlight that *Salmonella* exhibits a low rate of reduction in LMF, maintaining viability over extended storage periods.

*Salmonella* is the leading cause of outbreaks and recalls associated with low‐aw foods (Beuchat et al. 2013) (Table 1). In European Union (EU) countries, one in three foodborne disease outbreaks are caused by *Salmonella* (ECDC 2019a). The CDC estimates *Salmonella* bacteria cause about 1.35 million infections, 26,500 hospitalizations, and 420 deaths in the United States every year (CDC 2025a). Thereby, *Salmonella* contamination in low‐aw foods can arise from raw materials, ingredients, processing environments, or equipment. Once in the product destined for final consumption, *Salmonella* can remain viable for long periods, being an eminent risk of foodborne infection (Beuchat et al. 2013). Therefore, given *Salmonella*’s public health relevance, understanding its stress responses is crucial for its eradication from the food chain, especially since low‐aw foods are commonly ready‐to‐eat.

**TABLE 1:** Outbreaks of *Salmonella* infections associated with consumption of low‐aw foods from 2012 to 2025 and associated product recalls.

Year	Food	Serovar	Location	No. affected	Recall	Reference
2012	Peanut butter	*Salmonella* Bredeney	The United States	42	Yes	CDC (2012)
2013	Tahini	*Salmonella* Montevideo and *Salmonella* Mbandaka	The United States	16 (1 death)	Yes	CDC (2013)
2013	Pistachios	*Salmonella* Senftenberg	The United States	8	Yes	Whitham et al. (2021)
2013	Sugar cane	*Salmonella Virchow*	The United States	7	No	Whitham et al. (2021)
2014	Raw cashew cheese	*Salmonella* Stanley	The United States	17	Yes	CDC (2014a)
2014	Organic sprouted chia	*Salmonella* Newport, *Salmonella* Hartford, or *Salmonella* Oranienburg	The United States	31	Yes	CDC (2014b)
2014	Powder nut butter	*Salmonella* Braenderup	The United States	6	Yes	CDC (2014c)
2014	Sprouted chia seed powder	*Salmonella* Newport, *Salmonella* Hartford, *Salmonella* Oranienburg, and *Salmonella* Saintpaul	Canada	63	Yes	PHAC (2014)
2015	Moringa leaf	*Salmonella Virchow*	The United States	35	Yes	Whitham et al. (2021)
2016	Nut butter	*Salmonella* Paratyphi B variant L(+) tartrate(+)	The United States	13	Yes	CDC (2016a)
2016	Raw real organic shake and meal products	*Salmonella* Virchow	The United States	33	Yes	CDC (2016b)
2016	Pistachios	*Salmonella* Montevideo and *Salmonella* Senftenberg	The United States	11	Yes	CDC (2016c)
2016	Hazelnut	*Salmonella* Typhimurium	The United States	6	Yes	Whitham et al. (2021)
2017	Sesame seeds	*Salmonella* 11:z41:e,n,z15	Czech Republic, Germany, Greece, Luxembourg, and United Kingdom	47	Yes	ECDC (2017)
2018	Kratom powder	*Salmonella* I 4,[5],12:b:, *Salmonella* Heidelberg, *Salmonella* Javiana, *Salmonella* Okatie, *Salmonella* Weltevreden, and *Salmonella* Thompson	The United States	199	Yes	CDC (2018b)
2018	Dried coconut	*Salmonella* Typhimurium	The United States	14	Yes	CDC (2018a)
2018	Cereal	*Salmonella* Mbandaka	The United States	135	Yes	CDC (2018d)
2018	Spring pasta salad	*Salmonella* Sandiego	The United States	101	Yes	CDC (2018c)
2018	Cake mix	*Salmonella* Agbeni	The United States	7	Yes	CDC (2019a)
2018	Tahini	*Salmonella* Concord	The United States	8	Yes	CDC (2019b)
2018	Infant formula	*Salmonella* Agona	France, Spain	37	Yes	ECDC (2018)
2019	Cream puffs and mini chocolate eclairs	*Salmonella* Enteritidis	Canada	85 (3 death)	Yes	PHAC (2019)
2019	Infant formula	*Salmonella* Poona	France, Belgium, Luxembourg	32	Yes	ECDC (2019b)
2021	Cashew brie	*Salmonella* Typhimurium, *Salmonella* Chester, *Salmonella* Duisburg, and *Salmonella* Urbana	The United States	20	Yes	CDC (2021a)
2021	Sesame‐based products (tahini and halva)	*Salmonella* Amsterdam, *Salmonella* Havana, *Salmonella* Kintambo, *Salmonella* Mbandaka	Denmark, Germany, the Netherlands, Norway, Sweden	121	Yes	ECDC (2021)
2021	Salame sticks	*Salmonella I 4,[5],12:i:—*	The United States	34	Yes	CDC (2021b)
2022	Milk chocolate	*Salmonella* Typhimurium	United Kingdom, Ireland, France, Norway, the Netherlands	134	Yes	ECDC (2022)
2022	Peanut butter	*Salmonella* Senftenberg	The United States	21	Yes	CDC (2022)
2023	Dry dog food	*Salmonella* Kiambu	The United States	7	Yes	CDC (2024a)
2023	Flour	*Salmonella* Infantis	The United States	14	Yes	CDC (2024c)
2024	Charcuterie meats	*Salmonella* I 4:i:—	The United States	104	Yes	CDC (2024b)
2025	Pistachio cream	*Salmonella* Oranienburg	The United States	04	Yes	CDC (2025b)

Serovars capable of causing disease in humans belong to *Salmonella* Enterica subsp. *enterica*. Currently, there are more than 2500 serovars belonging to this species and described taxonomically (Gast and Porter 2020). Table 1 shows that low‐aw food outbreaks and recalls involve diverse serovars, often multiple per outbreak, likely reflecting the globalized food trade and the wide geographic origins of these strains.

The ubiquity of *Salmonella* in the food chain favors the emergence of highly stress‐resistant strains through mutations (Humphrey 2004). This pathogen adapts to osmotic and desiccation stresses via well‐regulated systems that reprogram its phenotype, including altered morphology, compatible solute production, suppression of virulence mechanisms, and metabolic shifts (Abdelhamid and Yousef 2020; Maserati et al. 2017; White et al. 2006). In addition, in some cases, such general stress responses also confer cross‐protection against other stresses (Empadinhas and Da Costa 2008; Maserati et al. 2017; Pereira et al. 2020; Zhao et al. 2016). These stress‐resistant variants are particularly concerning in LMF because osmotic and desiccation conditions select for phenotypes capable of surviving long‐term storage. This review discusses these molecular, phenotypic, and metabolic protective mechanisms, offering an integrated understanding of *Salmonella*’s responses to osmotic and desiccation stress.

## 2 Why Is *Salmonella* Resistant to Low Water Activity?

The optimal conditions necessary for the growth of *Salmonella* in a laboratory involve pH close to neutrality, temperature around 37°C, and aw values above 0.94 (Jay et al. 2005). Within a healthy host, however, *Salmonella* faces stable conditions such as constant temperature and pH, but once in external environments like food, it encounters heterogeneous factors, microbial competition, variable temperature, pH, water activity, light, and oxygen that act as selective pressures driving adaptive responses for long‐term survival (Beuchat et al. 2013; Humphrey 2004).

**FIGURE 2:**
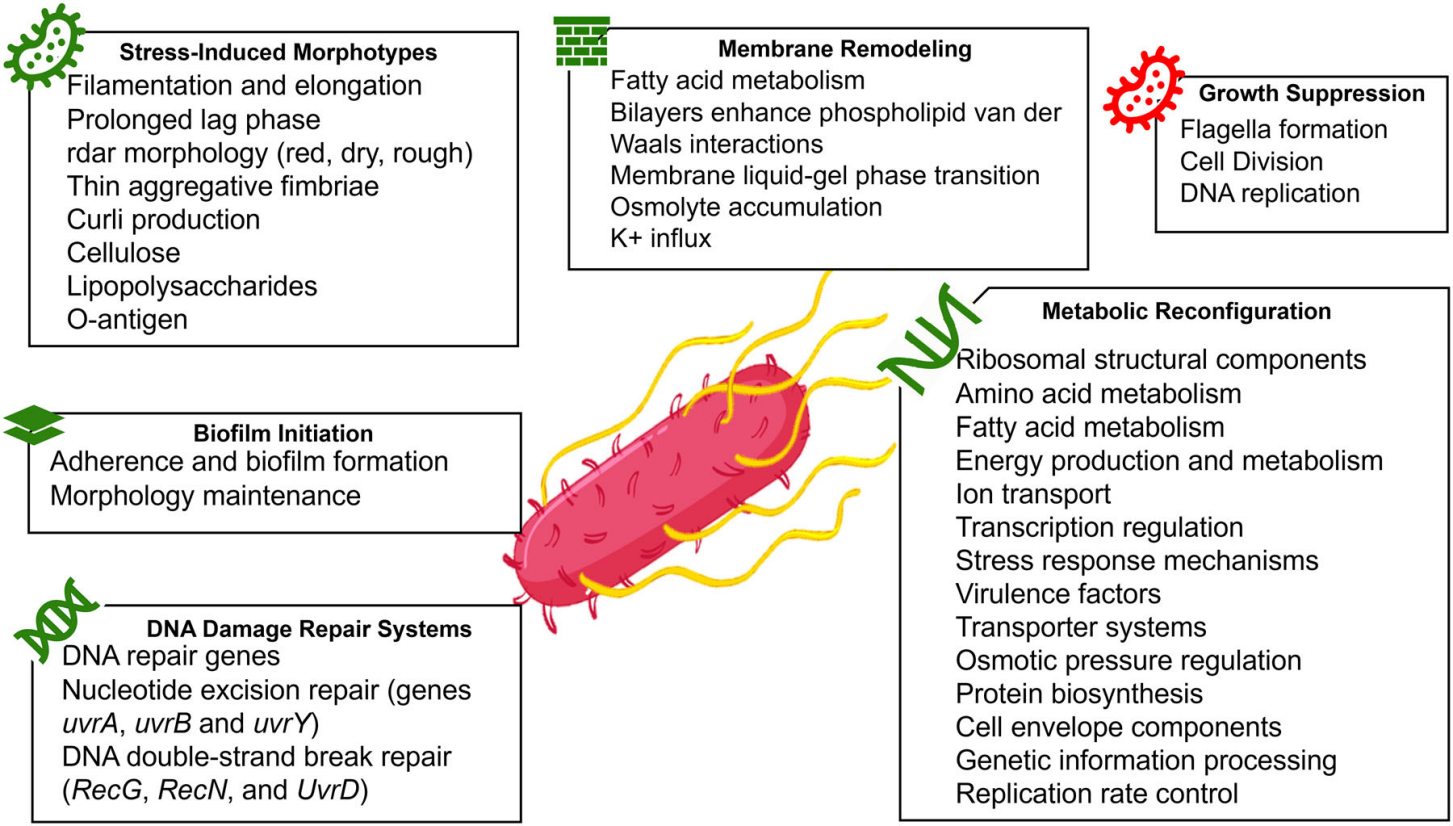
Molecular and phenotypic responses of *Salmonella* under osmotic and desiccation stresses. Green indicates increased expression; red indicates repression (vector illustration of the bacterium modified from brgfx/Freepik).

Nascimento *Salmonella*’s long‐term survival is enabled by multiple strategies, including morphological changes (e.g., filamentation), transcriptional shutdown, activation of sigma factors, altered cell composition, accumulation of compatible solutes, increased virulence and cross‐resistance, and entry into a dormant viable but non‐culturable (VBNC) state (Lebre et al. 2017; Mutz et al. 2020; Oliver 2005). These adaptations (Figure 2) ensure tolerance and persistence despite harsh environmental conditions.

Briefly, it is believed that under the desiccation stress, the cell initially increases the influx of potassium by the kdp transporter, increases the expression of osmoprotectant transport (proPU and osmU), synthesizes glutamate and trehalose, and up‐regulates the catabolism of fatty acid, Fe–S cluster, sigma factors (rpoE and rpoS), and ompC (Mandal and Kwon 2017). Details of these and other mechanisms of resistance to desiccation will be discussed in depth below.

### 2.1 Global Transcriptomic Under Osmotic and Desiccation Stresses

*Salmonella* under low‐aw stress changes its gene expression. This change in the pattern of the previously described genes depends on several factors, such as the type of osmotic stress applied, the time of exposure of the cells to these stresses, the matrix in which the cells are stressed, the techniques used to determine gene expression, and the stability (half‐life) of the mRNA transcribed under the studied conditions (Crucello et al. 2019; Jenniches et al. 2024).

Studies show that *Salmonella* rapidly alters gene expression under osmotic or desiccation stress, both immediately and after prolonged exposure (Aviles et al. 2013; Chen and Jiang 2017; Mutz et al. 2020). Aviles et al. (2013) reported that *Salmonella* Tennessee upregulated stress‐resistance and virulence genes within 2 h after drying to 0.3 aw, though expression stabilized during prolonged exposure. Similarly, Chen and Jiang (2017) found that *Salmonella* Typhimurium upregulated resistance genes within 1 h of desiccation, but changes were not sustained beyond 2 h, nor during 3–24 h of adaptation. These findings highlight *Salmonella*’s rapid physiological responses as critical for long‐term persistence. Under osmotic shock from drying in inert or food matrices, or exposure to high osmolarity from salts or sugars, cells activate integrated protective mechanisms that prevent collapse. Transcriptomic analyses further reveal differentially expressed genes linked to ribosomal structure, amino acid and fatty acid metabolism, energy production, ion transport, transcription, stress response, and virulence (Gruzdev, McClelland, et al. 2012; Li et al. 2012; Maserati et al. 2017).

Several studies have performed global transcriptomic and proteomic analyses of *Salmonella* under low‐aw conditions (Gruzdev, McClelland, et al. 2012; Li et al. 2012; Maserati et al. 2017, 2018). These works identified differentially expressed genes associated with fatty acid metabolism, transport systems, osmotic stress responses, protein biosynthesis, cell envelope functions, ion transport, genetic information processing, and virulence. Together, these findings indicate that under low aw, cells adopt a highly regulated state aimed at conserving energy, stabilizing genetic material, and maintaining essential molecular functions, rather than supporting active replication and metabolism (Maserati et al. 2018).

Although transcriptomic studies often reveal overlapping gene expression, the specific resistance genes identified and grouped into functional categories vary. For instance, Li et al. (2012) reported increased expression of fatty acid metabolism genes in *S*. Tennessee and LT2 after 2 h of air‐drying at 11% relative humidity, whereas Gruzdev McClelland, et al. (2012) found that dehydration in *S*. Typhimurium upregulated 90 genes and downregulated 7, with ribosomal and protein biosynthesis genes most affected. The authors conclude that, instead of shutting down translation (as expected under starvation), dehydration triggers a need for de novo protein synthesis, inducing a strong upregulation of ribosomal genes, translation factors, and protein‐folding chaperones, indicating an elevated need for protein biosynthesis and turnover to support stress adaptation and survival. Maserati et al. (2018) further showed that desiccation and thermal stress caused major proteomic changes, primarily in metabolism, followed by genetic and environmental information processing. These findings suggest that, beyond osmotic stress, multiple factors influence the expression of resistance‐related genes and proteins during *Salmonella*’s desiccation.

### 2.2 DNA Recombination and Repair Mechanisms

Exposure to desiccation and low aw can cause DNA damage in bacteria, including covalent modifications and double‐strand breaks (Humann and Kahn 2015; Maserati et al. 2018). To counteract these effects, many microorganisms, such as *Deinococcus radiodurans* (Tanaka et al. 2004), *Bradyrhizobium japonicum* (Cytryn et al. 2007), and *Salmonella* spp. (Jayeola et al. 2020), upregulate DNA repair genes under such stress conditions (Tanaka et al. 2004).

In a study by Jayeola et al. (2020), the authors used transposon sequencing on *Salmonella* Enteritidis, *S*. Typhimurium, and *S*. Newport inoculated in pistachios (aw 0.32) stored at 25°C for 120 days, finding that DNA recombination and repair genes (uvrA, uvrB, uvrD, recN, recG, Dam, and Dcm) were significantly underrepresented (*p* < 0.05) in mutant populations. These results suggest that low humidity causes DNA damage in *Salmonella* and that activation of DNA repair systems is essential for survival.

UvrA, UvrB, and UvrD proteins are central components of the highly conserved nucleotide excision repair systems in prokaryotes (Jayeola et al. 2020; Kisker et al. 2013). RecG, RecN, and UvrD are involved in recombinational repair of DNA damage that often involves double‐stranded DNA breaks (Jayeola et al. 2020; Kuzminov 2011). DNA adenine methyltransferase (Dam) and DNA cytosine methyltransferase (Dcm) are responsible for the majority of methylated DNA bases in *Escherichia coli* and *Salmonella* (Jayeola et al. 2020; Marinus and Løbner‐Olesen 2014).

Findings from Jayeola et al. (2020) show that uvrA, uvrB, and uvrY were selected during both drying (1 day) and storage (120 days) of *Salmonella* in pistachios, whereas Dam and Dcm were selected only during drying, and uvrD, recN, and recG only during storage. According to the authors, this differential selection indicates that nucleotide excision (lesion detection, excision of damaged bases, repair synthesis) and methylation‐directed DNA repair (mismatch repair signaling, methylation, SOS regulation, oxidative stress tolerance) pathways are especially critical during drying, when viability is most affected.

DnaJ and UvrD proteins were found to be significantly more abundant in dried *S*. Typhimurium on glass beads as the water activity decreased to 0.11 aw (Maserati et al. 2018). However, when *Salmonella* was desiccated on other abiotic surfaces such as glass beads, plastics, and stainless steel, none of the DNA recombination and repair genes were differentially expressed in transcriptome analyses (Finn, Händler, et al. 2013; Gruzdev, McClelland, et al. 2012; Li et al. 2012; Maserati et al. 2017).

### 2.3 Morphological Adaptation and Cell Division

When bacteria are under stressful conditions, such as being faced by low water availability, blocking of DNA replication may occur as part of the SOS response (White et al. 2012). In the context of genetics, the use of “SOS” indicates that a cell is responding to a distress signal generated by DNA (White et al. 2012). Cell division and DNA metabolism are coupled. That is, cell division is inhibited whenever DNA replication or partitioning is blocked (White et al. 2012).

Under reduced aw, *Salmonella* stops division and septation, forming filaments up to 200 µm long compared to the normal 2 µm, as observed in *Salmonella* Enteritidis and *S*. Typhimurium at 0.95 aw and 4–8°C (Finn, Condell, et al. 2013; Li et al. 2012; Mattick et al. 2003). These elongated cells contain regularly spaced nucleoids, showing that chromosome replication and segregation continue without septum formation, consistent with an early block in cell‐division gene transcription (Humphrey 2004; Mattick et al. 2000, 2003 Phillips et al. 1998).

Li et al. (2012) reported that in *S*. Tennessee and *S*. Typhimurium LT2 exposed to 2 h of air‐drying at 11% RH, expression of cell division genes (fts) remained stable or moderately upregulated, whereas MinCD was upregulated in the more desiccation‐resistant *S*. Tennessee. In *E. coli*, the MinCDE system prevents mislocalization of the FtsZ ring during division (Huang et al. 2003; Rothfield et al. 1999), and similarly, filamentous *Salmonella* cells exhibit normal FtsZ levels despite the absence of septation (Humphrey 2004).

Besides impaired FtsZ ring polymerization, other mechanisms contribute to *Salmonella* filamentation under low‐aw stress. DNA damage can activate the SOS response, inducing SulA synthesis, which blocks FtsZ septum formation and inhibits division (White et al. 2012). Division also depends on turgor pressure, and under high osmotic or low‐aw conditions, the lack of signaling prevents cell division (Hecker et al. 1988; Humphrey 2004). Additionally, Mandal and Kwon (2017) showed that tpiA is essential for desiccation survival in *S*. Typhimurium 14028S, as mutants lacking this gene, encoding the glycolytic enzyme triosephosphate isomerase, display elongated morphology and reduced fitness (Paterson et al. 2009).

Under sublethal low‐aw stress, *Salmonella* requires an adaptation period resembling the lag phase before filament formation begins. Mattick et al. (2003) showed this lag period can last about 48 h, with filaments appearing after 5 days at 0.95 aw. The slow generation time is partly linked to increased expression of SeqA, a DNA‐binding protein that sequesters oriC and prevents replication initiation, observed in cells desiccated to 0.11 aw (Brendler et al. 1995; Maserati et al. 2018; Nievera et al. 2006; Waldminghaus et al. 2012; Yamazoe et al. 2005). Once stress is relieved, septation resumes rapidly, producing numerous daughter cells within 2 h (Mattick et al. 2000, 2003). Filamentation benefits the pathogen by conserving energy when reserves are low and reducing the risk of forming anucleate cells (Mattick et al. 2003; White et al. 2012).

*Salmonella*’s resistance to desiccation is also linked to its ability to preserve morphology under extreme stress. Desiccation induces upregulation of the mreBCD operon, suggesting its role in maintaining membrane composition and cell shape (Maserati et al. 2017). Mre proteins are cytoskeletal elements essential for elongation and rod shape in nonspherical bacteria (Maserati et al. 2017; Van den Ent et al. 2001). Mutations in mreC cause *E. coli* and *Salmonella* to become spherical (Bendezú and De Boer 2008; Bulmer et al. 2012), whereas mreB mutants in *Bacillus subtilis* round up and lyse (White et al. 2012).

Using scanning electron microscopy, Maserati et al. (2017) showed that *S*. Typhimurium equilibrated to 0.11 aw exhibited surface corrugation and cell debris, whereas sopD/sseD mutants displayed the rounded morphology seen in mreC mutants of *Salmonella* and *E. coli* (Bendezú and De Boer 2008; Bulmer et al. 2012). Although a direct link between sopD/sseD and the mre operon was not established, the reduced desiccation tolerance of these mutants suggests that desiccation response may involve sopD/sseD‐dependent induction of the mre operon (Maserati et al. 2017).

### 2.4 Phenotypic Changes

Several studies suggest that *Salmonella* relies on filamentation and the rdar (red, dry, and rough) morphotype as survival strategies under long‐term starvation and desiccation (Mattick et al. 2003; White et al. 2006, 2008). The rdar phenotype, characterized by rough, spreading colonies on Congo red agar (Davidson et al. 2008), has been proposed to act as a resting stage comparable to spore formation in Gram‐positives. However, other studies reported that genes regulating rdar morphology, including the agfD promoter, were not upregulated during desiccation (Chen and Jiang 2017; Finn, Händler, et al. 2013; Fong and Wang 2016; Li et al. 2012), suggesting that although filamentation is critical for long‐term persistence in low‐aw environments, rdar morphology may not always be involved. Similarly, genes encoding curli fimbriae (csgDEFG, csgBAC) and cellulose (bcsABZC) were not consistently upregulated under desiccation (Finn, Händler, et al. 2013; Gruzdev, McClelland, et al. 2012; Li et al. 2012).

Despite this, *Salmonella* often undergoes phenotypic shifts under osmotic and desiccation stress, including repression of flagella synthesis and induction of curli, fimbriae, cellulose, lipopolysaccharides, and O‐antigen (Finn, Condell, et al. 2013; Finn, Hinton, et al. 2013; Gibson et al. 2006; Maserati et al. 2017; White et al. 2006). For instance, *S*. Tennessee suppressed flagella production within 2 h at 11% RH, likely as an energy‐saving strategy to enhance survival under starvation and desiccation (Li et al. 2012). These components are also essential for biofilm formation, facilitating adherence to surfaces and other bacteria (Bowman et al. 2015; Maserati et al. 2017; White et al. 2006; Zogaj et al. 2001). Curli fimbriae, in particular, are the key for stable biofilm development (Finn, Hinton, et al. 2013). In a survey of 46 isolates, Finn, Condell, et al. (2013) found that most could form biofilms, with 57% producing curli fimbriae and 75% producing cellulose; all rdar‐positive isolates were also strong cellulose producers. In the same study, strains combining curli and cellulose production with pellicle formation were the most robust biofilm formers, reinforcing the link between these traits and *Salmonella*’s persistence in low‐moisture environments.

### 2.5 Biofilms

Biofilms are a significant challenge in the food industry because they adhere to surfaces and resist sanitation, largely through extracellular polymeric substances (EPS) (Aryal and Muriana 2019). CsgD is the master regulator of biofilm production (Fàbrega and Vila 2013; Mutz et al. 2020), controlling determinants such as thin aggregative fimbriae and extracellular cellulose, which form dense networks and create the hydrophobic environment characteristic of *Salmonella* biofilms (Aviles et al. 2013; Solano et al. 2002; Zogaj et al. 2001).

Biofilms are communities where bacteria aggregate with themselves and with other bacteria and organisms to form multicellular structures (White et al. 2012). During the biofilm formation, EPS are released by the bacteria, and in some cases, extrusion of DNA also occurs (White et al. 2012). In general, production of EPS has been associated to a higher desiccation tolerance in a variety of bacterial species, such as *E. coli*, *Acinetobacter calcoaceticus*, *Erwinia stewartii*, *Rhizobium sullae*, and *S*. Enterica (Gharzouli et al. 2013; Maserati et al. 2017; Ophir and Gutnick 1994). It is believed that EPS works as a water reservoir and protects against desiccation (Maserati et al. 2018; Roberson and Firestone 1992). It also adds nutrients by residues released (Billi and Potts 2002; Gruzdev, McClelland, et al. 2012; Gruzdev, Pinto, et al. 2012; Potts 1994).

Biofilms enhance *Salmonella* survival under nutrient limitation and low‐aw conditions common in LMF processing plants (Aviles et al. 2013). Finn, Condell, et al. (2013) found a strong correlation between biofilm formation and survival in LB broth with humectant solutes, with *Salmonella* Agona (SU26), isolated from LMF, identified as the strongest biofilm former. On spices, biofilms showed no reduction in *Salmonella* after 28 days at 0.3 aw, and microscopy revealed coccoid and rod‐shaped cells embedded in a mucoid, network‐like layer likely composed of exopolysaccharides, proteases, and cellulose, key components of *Salmonella* biofilms (Bowman et al. 2015). In fact, biofilm formation has been demonstrated on a variety of surfaces relevant to LMF processing environments, including polypropylene (von Hertwig et al. 2023), glass beads (Maserati et al. 2017), and LMF products such as powdered milk (Chen et al. 2021). This may indicate that biofilm structures present in the industry of LMF formed by bacteria of other genera may become a reservoir for *Salmonella*.

Biofilm formation during desiccation helps *Salmonella* maintain its morphology. Maserati et al. (2017) showed that dried WT cells remained rod‐shaped, though some displayed corrugated surfaces indicating loss of turgidity and were embedded in a thick extracellular matrix. In contrast, the author presented that sopD/sseD mutants were smaller, spheroidal, and unable to form mature biofilms. Although ΔsopD produced extracellular material, it lacked the three‐dimensional structure of the WT, attaching instead to the bead surface with discontinuities where cells had detached. The study continued, and after 7 days at 0.11 aw, both mutants lacked extracellular matrix altogether, suggesting that virulence genes support cell morphology and biofilm development. Similarly, Aviles et al. (2013) compared planktonic and biofilm *Salmonella* cells in dry milk powder (0.3 aw) under in vitro digestion and found virulence gene expression increased after short‐term dry storage, with the highest expression in biofilm cells stored for 30 days, indicating enhanced virulence potential.

Besides the multicellular morphology, changes in outer membrane lipopolysaccharides have also been reported as the major responses of *Salmonella* to desiccation (Gibson et al. 2006; Li et al. 2012; Mattick et al. 2000; White et al. 2008). Studies have shown that mutants with altered lipopolysaccharides are more sensitive to desiccation than their parental *Salmonella* strains (Garmiri et al. 2008).

### 2.6 Cell Membrane Adaptations

The cellular membrane is the primary barrier between the intracellular space and the external environment (Lebre et al. 2017). It senses osmotic pressure and other environmental changes through protein conformational shifts, membrane stretching, and possibly lipid–protein interactions (Beney et al. 2004; Denich et al. 2003; Poolman and Glaasker 1998; Wood 1999). Reduced aw adversely affects membrane function, which depends on maintaining fluidity, a critical feature for survival under desiccation stress (Gruzdev, McClelland, et al. 2012; Gruzdev, Pinto, et al. 2012).

To maintain the functioning of the membrane and consequently the cell viability in restricted conditions of water availability, it has been demonstrated that *Salmonella* can undergo several changes in the membrane (Finn, Händler, et al. 2013; Gruzdev, McClelland, et al. 2012; Gruzdev, Pinto, et al. 2012; Li et al. 2012). When *Salmonella* is under stress of low water and nutrient availability, there is an increase in fatty acid metabolism because the cell needs energy, and the osmotic pressure induces a need for a more rigid membrane (Li et al. 2012).

In a transcriptome study, Li et al. (2012) demonstrated that the expression of genes associated with fatty acid metabolism was the highest and constituted 51% and 35% of the total expression fold change in *S*. Tennessee and *S*. Typhimurium LT2, respectively, in response to 2 h of air‐drying at 11% equilibrated relative humidity.

Under low‐aw conditions, *Salmonella* increases fatty acid catabolism to meet energy demands, enhancing oxidation pathways that provide efficient ATP production (Finn, Händler, et al. 2013). Among the differentially expressed genes, fadA (3‐ketoacyl‐CoA thiolase) is notably upregulated, catalyzing the final step of β‐oxidation that converts long‐chain fatty acids into acetyl‐CoA for the TCA cycle (Chen and Jiang 2017; Fong and Wang 2016; Li et al. 2012). As fatty acid oxidation yields more ATP per carbon atom than glucose, it represents a highly cost‐effective energy source for survival under desiccation stress (Li et al. 2012).

The isocitrate lyase gene (aceA) is upregulated in dehydrated and water‐suspended *S*. Typhimurium (Gruzdev, McClelland, et al. 2012). Although indirectly linked to fatty acid metabolism, AceA plays a key role in metabolic adaptation, participating in the O‐antigen cycle and enabling the use of C2 compounds for gluconeogenesis when glucose is unavailable (Eprintsev et al. 2014; Kornberg and Krebs 1957). It catalyzes the reversible conversion of isocitrate into succinate and glyoxylate, a central step in the glyoxylate cycle, which replenishes the TCA cycle during growth on fatty acids (UniProt). aceA mutants showed reduced resistance after 12 weeks of storage at 4°C and 25°C, with even lower survival at 25°C compared to the wild type (Gruzdev, McClelland, et al. 2012). These findings indicate that under glucose and water restriction, *Salmonella* sustains viability by complementing energy generation through fatty acid oxidation and the glyoxylate cycle.

Other genes that have also been found upregulated in the metabolism of fatty acids during *Salmonella* desiccation are associated with two short‐chain fatty acids, propionic (prpEBCD), and butyric acid (gabTBD) (Li et al. 2012). The ddg gene, encoding lipid A biosynthesis palmitoleoyl acyltransferase was also upregulated in *S*. Enterica under desiccation. Ddg modifies the lipid A composition in bacteria and enables the cells to maintain optimal outer membrane fluidity (Gruzdev et al. 2012). Knockout mutations in the ddg gene showed that this gene implies involvement in the adaptation of *Salmonella* to desiccation (Gruzdev et al. 2012).

Under drying stress, increased van der Waals interactions between phospholipids shift membranes from a liquid to gel phase, causing segregation, vesicle formation, and solute leakage (Beney et al. 2004; Gruzdev, McClelland, et al. 2012; Gruzdev, Pinto, et al. 2012; Potts 1994). To counteract this, *Salmonella* accumulates or releases compatible solutes to maintain osmotic balance, notably boosting trehalose biosynthesis from glucose (Finn, Condell, et al. 2013).

### 2.7 Accumulation of Compatible Solutes

Evaporation in the environment or food increases solute concentration, creating a hypertonic setting for bacteria. To prevent water loss and metabolic collapse, cells must balance intracellular and extracellular osmotic pressure (Chen and Jiang 2017; Csonka 1989). As water evaporates, weakened hydrophobic interactions destabilize proteins, leading to denaturation and membrane damage (Maserati et al. 2018; Poolman et al. 2002). In this regard, viability depends on minimizing this damage, maintaining turgor pressure, and activating protective cellular responses (Maserati et al. 2018; Poolman et al. 2002). *Salmonella* and other bacteria achieve this through molecular mechanisms that regulate internal solute concentrations to sustain proper turgor (Maserati et al. 2017; Poolman et al. 2002; Wood et al. 2001).

The turgor pressure is maintained by increasing the influx of compatible solutes or osmoprotectors, whose nomenclature is given because they do not present cellular toxicity. The osmoprotectors are small molecules with low‐molecular weight that stabilize proteins and lipids (Csonka and Hanson 1991; Maserati et al. 2017, 2018; Pilonieta et al. 2012; White et al. 2012). Some known osmoprotectants include proline, betaine, choline, and trehalose (Csonka 1989; Finn, Händler, et al. 2013), and some of the genes related to these osmoprotectors were found upregulated in *Salmonella* during desiccation conditions (Li et al. 2012). Osmoprotectants can be intracellularly accumulated from the medium by ABC or de novo synthesis (da Costa et al. 1998; Empadinhas and Da Costa 2008; Roeßler and Müller 2001).

Genes involved in osmoprotectant uptake, including proP, proU (proVWX), and osmU (osmVWXY), are upregulated in *S*. Typhimurium after 4 h on steel surfaces (Finn, Händler, et al. 2013) and at 0.11 aw (Maserati et al. 2017). proP encodes a major facilitator superfamily permease, whereas proU and osmU encode ABC transporters (Finn, Händler, et al. 2013; Frossard et al. 2012; MacMillan et al. 1999; Stirling et al. 1989). All three systems enhance survival under NaCl and desiccation stress by increasing osmoprotectant influx, thereby protecting cells from osmotic shock (Chen and Jiang 2017; Dunlap and Csonka 1985; Finn, Händler, et al. 2013; Frossard et al. 2012; Li et al. 2012; Maserati et al. 2017).

The proP, proU, and osmU transport systems are essential for *Salmonella* survival under desiccation, as deletion of any reduced *S*. Typhimurium viability, with proP showing the greatest impact, followed by osmU and proU (Finn, Händler, et al. 2013; Frossard et al. 2012). Although proU and osmU mutants survived longer than proP mutants, even proP deletions persisted up to 4 weeks, indicating that additional mechanisms contribute to osmotic protection (Finn, Händler, et al. 2013). A transcriptome study also identified a novel small RNA (sRNA2111563) predicted to repress proP; its downregulation after 72 h of desiccation suggests increased proP expression under stress (Barnhill et al. 2019).

The otsAB genes, responsible for trehalose biosynthesis, are crucial for *Salmonella*’s tolerance to low‐aw environments (Finn, Händler, et al. 2013; Fong and Wang 2016; Li et al. 2012). Their expression, possibly linked to regulation of the osmU ABC transporter, is induced under desiccation but declines once cells are rehydrated, highlighting their specific role in low‐moisture survival (Finn, Händler, et al. 2013). Li et al. (2012) showed that *S*. Tennessee strongly upregulated otsAB and accumulated trehalose during desiccation, whereas the less resistant LT2 strain did not, confirming trehalose production as a key mechanism of resistance.

Trehalose metabolism shifts depending on osmolarity. At low osmolarity, the treBC genes encode enzymes that convert trehalose into glucose, whereas TreB and Crr transport periplasmic trehalose into the cell (Horlacher et al. 1996; KEGG n.d.). At high osmolarity, TreA recaptures extruded trehalose in the periplasm and hydrolyzes it to glucose, enabling *Salmonella* and *E. coli* to use trehalose as a carbon source, whereas TreF, a cytoplasmic trehalase homologous to TreA, is weakly induced under these conditions (Horlacher et al. 1996). The otsAB genes, instead, direct glucose conversion into trehalose and are not induced by extracellular trehalose (Horlacher et al. 1996; Klein et al. 1995). Li et al. (2012) reported increased treBC activity in desiccation‐resistant *S*. Tennessee and treA activity in LT2 at 0.11 aw, suggesting TreA and TreF play complementary roles in desiccation resistance. The induction of treF under desiccation indicates that *Salmonella* catabolizes trehalose to glucose, providing an efficient energy source during stress (Finn, Händler, et al. 2013; Horlacher et al. 1996).

Trehalose, a nonreducing glucose disaccharide, protects cell membranes from osmotic stress in many organisms (Elbein et al. 2003; Li et al. 2012). Its α,α‐(1 → 1) glycosidic bond allows a clamshell‐like structure that promotes interactions with membrane lipid head groups (Albertorio et al. 2007; Maserati et al. 2018). In *E. coli*, trehalose has been shown to help maintain membrane integrity and enhance desiccation resistance (Fang et al. 2020). It may also prevent lipid oxidation by forming stable complexes between its hydroxyl groups and the double bonds of unsaturated fatty acids (Fang et al. 2020; Oku et al. 2003).

The prevention of intracellular water loss during osmotic stress due to trehalose is given because this compatible solute binds water that would otherwise be lost from the cell during drying and dry environment (Maserati et al. 2018). Furthermore, this disaccharide has been found to decrease intracellular fluidity through a process called vitrification. Vitrification decreases the diffusion and, therefore, the accumulation rates of reactive oxygen species (ROS), thus slowing the deterioration of cellular components (Crowe et al. 1984; Lebre et al. 2017; Maserati et al. 2018).

Alongside trehalose, betaine and proline play key roles in *Salmonella*’s resistance to desiccation. The proV gene encodes the energy‐coupling component of the ProU system for glycine betaine uptake and is strongly induced under low‐aw stress, underscoring its importance for survival (Li et al. 2012; Finn, Condell, et al. 2013; Finn, Händler, et al. 2013; Chen and Jiang 2017; May et al. 1989). Proline is transported by three systems, PutP, ProP, and ProU, with PutP mediating sodium‐dependent uptake of extracellular L‐proline (Finn, Händler, et al. 2013; Jay et al. 2005). Although these osmoprotectants enhance desiccation tolerance, trehalose stands out as the most critical due to its combined roles in energy generation, water retention, and membrane protection (Y. Fang et al. 2020; Finn, Händler, et al. 2013; Li et al. 2012).

Osmotic regulation models suggest that the first response to dehydration is an influx of K^+^ through the high‐affinity transport system encoded by the kdp operon, because potassium serves as a major compatible osmolyte (White et al. 2012). Induction of KdpFABC in enterobacteria is triggered by dehydration, increasing intracellular ion strength and contributing to turgor loss (Ballal et al. 2007; Epstein 2003; Gruzdev, McClelland, et al. 2012; Gruzdev, Pinto, et al. 2012). Consistently, Gruzdev, McClelland, et al. (2012) and Gruzdev, Pinto, et al. (2012) found that kdpABC genes were the most highly upregulated after 22 h of desiccation, supporting this activation model.

The initial rise in potassium uptake can acidify the cytoplasm, leading to the formation of potassium glutamate, a key strategy for maintaining acid–base balance (White et al. 2012). Glutamate inhibits global transcription by σ^70^ while activating σ^38^, which regulates many stress‐response genes, including osmY, a major osmotic shock‐induced periplasmic protein (Lange et al. 1993; Lee and Gralla 2004; Yim and Villarejo 1992). As glutamate‐driven transcription progresses, other osmolytes accumulate and help restore the activity of various RNA polymerases (Lee and Gralla 2004).

The initial influx of potassium ions also appears to induce proU and proP, which signal the uptake of other osmoprotectants (Balaji et al. 2005; Booth and Higgins 1990; Epstein 1986; Finn, Händler, et al. 2013; S. J. Lee and Gralla 2004; White et al. 2012). When glucose is available, trehalose is synthesized but can later be replaced by betaine as trehalose enters catabolism (Larsen et al. 1987; Lee and Gralla 2004). Under high osmolarity, *E. coli* and *Salmonella* halt division, resuming slow growth only after these protective molecules accumulate in the cytoplasm (Wood 1999; Lee and Gralla 2004; Mattick et al. 2003; Li et al. 2012; Finn, Condell, et al. 2013; Finn, Händler, et al. 2013).

On the other hand, some studies challenge this model of osmotic regulation. Balaji et al. (2005) proposed that proU and proP are upregulated before activation of potassium transport systems like kdpFABC in *S*. Typhimurium. Finn, Condell, et al. (2013) and Finn, Händler, et al. (2013) supported this view, reporting no >5‐fold induction of potassium transport genes during desiccation on stainless steel or after 4 h rehydration. Similarly, Maserati et al. (2017) observed induction of osmV but not kdpFABC after 4 days at 0.11 aw. Deletion studies further showed that although kdpA is not essential for desiccation tolerance, it may contribute to long‐term persistence under storage (Gruzdev, McClelland, et al. 2012).

In view of these divergences, Mutz et al. (2020) summarized *Salmonella*’s osmotic regulation under hyperosmotic stress, showing that responses vary with exposure time. In the short term, K^+^ uptake occurs through the low‐affinity Trk system, whereas ProU, ProP, and OsmU transporters are induced, and the high‐affinity Kdp system is activated. Over longer exposure, ProU, OsmU, and ProP mediate the active uptake of compatible solutes, and sigma factors drive transcription of the otsBA operon, enabling trehalose synthesis from glucose.

Transcriptome studies suggest that *Salmonella* may limit K^+^ accumulation under desiccation because excess uptake would raise intracellular pH, while metabolism often shifts to a static or VBNC state under high osmolarity, making the recruitment of solutes like glutamate insufficient to prevent cell death. As White et al. (2012) noted, K^+^ influx poses two challenges: maintaining cytoplasmic electrical neutrality and preventing membrane depolarization, which would reduce potential energy (∆*p*). Although enterobacteria usually have mechanisms to manage these issues, extreme stress can overwhelm them, forcing reliance on alternative survival strategies.

#### 2.7.1 How the Osmoprotectants End Up in the Cell

**FIGURE 3:**
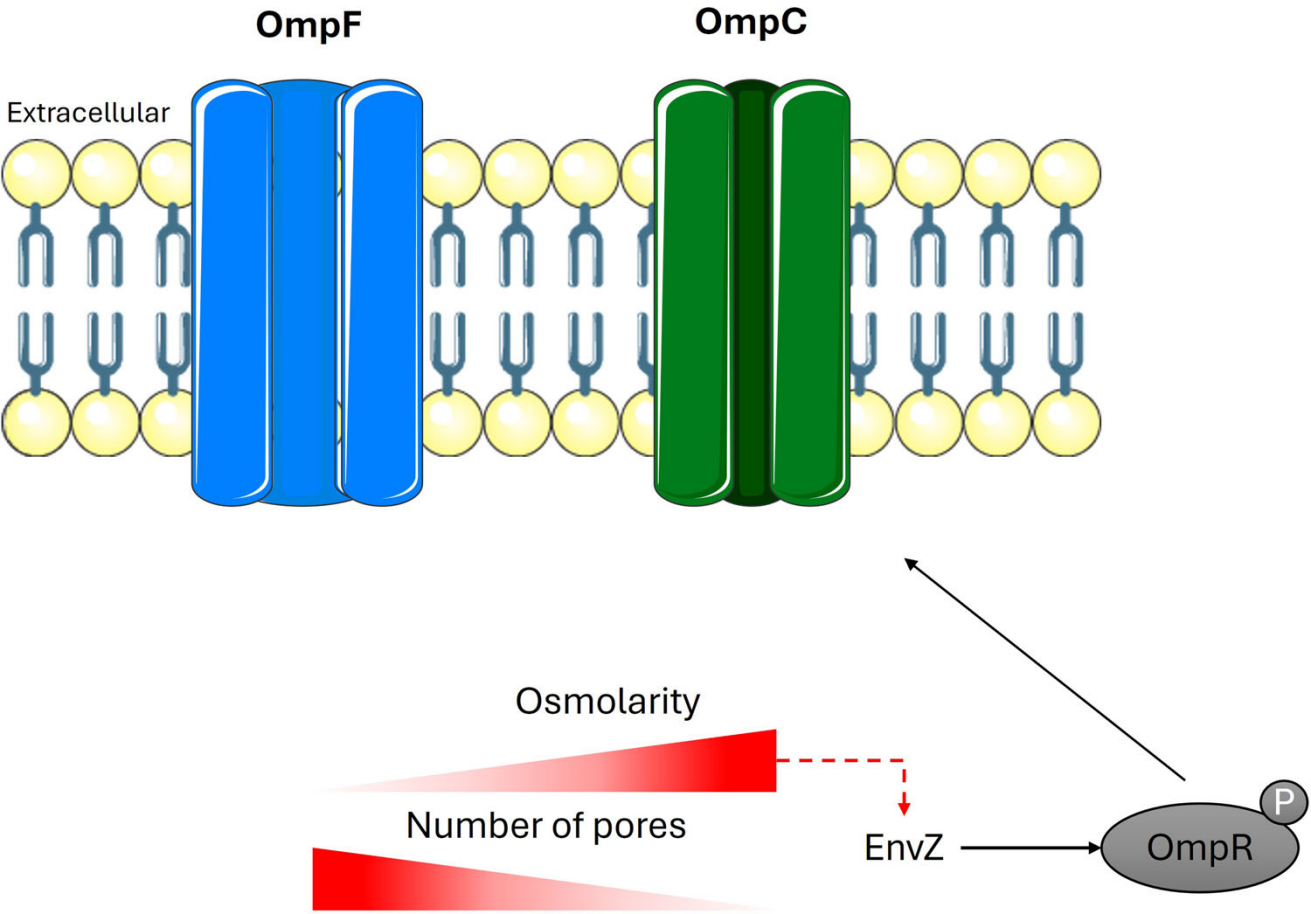
Regulation of OmpF and OmpC porins in response to osmolarity. Under low‐osmolarity conditions, *Salmonella* predominantly expresses the OmpF porin. When environmental osmolarity increases, the EnvZ/OmpR two‐component system becomes activated, resulting in the upregulation of OmpC and the repression of OmpF (Finn, Condell, et al. 2013; Finn, Händler, et al. 2013; Kempf and Bremer 1998; Feng et al. 2003; Hall and Silhavy, 1981a, 1981b; Wang et al. 2012; Mutz et al. 2020). Image credit: The figure was drawn by using modified pictures from Servier Medical Art. Servier Medical Art by Servier is licensed under a Creative Commons Attribution 3.0 Unported License <https://creativecommons.org/licenses/by/3.0/>.

To survive desiccation, *Salmonella* must sense changes in environmental osmolarity (Jayeola et al. 2020). In enterobacteria, the OmpF and OmpC porins of the plasma membrane mediate passive diffusion of osmoprotective substances (Finn, Condell, et al. 2013; Kempf and Bremer 1998) (Figure 3). Their expression is regulated by the two‐component system EnvZ/OmpR (X. Feng, Oropeza, Walthers, et al. 2003; Finn, Condell, et al. 2013; M. N. Hall and Silhavy 1981a, 1981b; Wang et al. 2012) and depends on external osmolarity. Under normal or low osmolarity, OmpF predominates. When osmolarity increases, the sensor kinase EnvZ activates OmpR, which in its phosphorylated form (OmpR‐P) represses OmpF and upregulates OmpC, making OmpC the dominant porin in the membrane (Mutz et al. 2020). The key difference between them lies in pore size: OmpC has a smaller channel, reducing flux, blocking larger molecules, and favoring diffusion of small hydrophilic compounds (X. Feng, Oropeza, Walthers, et al. 2003; X. Feng, Oropeza, Kenney, et al. 2003; Mutz et al. 2020).

The two‐component regulator EnvZ/OmpR plays an important role in long‐term survival. *Salmonella* mutants with Tn5 insertions in envZ and ompR showed reduced persistence on pistachios (Jayeola et al. 2020). Under osmotic shock with NaCl, OmpC transcription was detected after 12 min (Balaji et al. 2005), but under desiccation stress, this shift from OmpR to OmpC expression did not occur (Deng et al. 2012; Finn, Händler, et al. 2013; Gruzdev, McClelland, et al. 2012; Li et al. 2012; Mandal and Kwon 2017). These findings suggest that the predominant OmpC phenotype may be critical for adaptation to low‐aw foods, where NaCl is the main humectant (Finn, Condell, et al. 2013). However, if ompC mRNA has a short half‐life, desiccation systems may fail to capture this signal due to RNA degradation (Finn, Condell, et al. 2013). Overall, the differential regulation of OmpF and OmpC in *E. coli* and *Salmonella* under varying osmolarity is central to their ability to remain stable in both low‐ and high‐osmolarity environments.

## 3 Virulence

Virulence is the ability of a pathogen to overcome host defenses. After ingestion of contaminated food, *Salmonella* faces multiple barriers in the digestive tract that threaten its survival (Mutz et al. 2020). To persist, it employs virulence mechanisms involving the synthesis of proteins and other factors that alter membrane or cytosolic composition, ultimately influencing the host's stress response during infection (Lang et al. 2017).

During LMF processing, shifts in osmolarity help *Salmonella* adapt to varying solute concentrations in the host digestive tract, enabling it to better withstand osmotic stress during infection (Altendorf et al. 2009; A. H. Potts et al. 2019). This tolerance may enhance its pathogenic potential and compromise LMF safety, because preservation barriers applied during processing may not fully eliminate the pathogen. Moreover, exposure to sublethal stresses during manufacturing can further increase *Salmonella*’s resistance once inside the host (Mutz et al. 2020).

The steps preceding LMF drying, often considered sublethal stress to contaminating microbiota, can negatively affect food safety. Drying conditions must be carefully monitored to avoid triggering metabolic pathways that enhance pathogen invasiveness. In milk powder, drying from an aw of 0.80 followed by thermal treatments increased the invasion capacity of *S*. Typhimurium and *Salmonella* Senftenberg in Caco‐2 cells from the onset of drying (Lang et al. 2017). Further decreases to aw 0.58 and 0.25 did not intensify this effect. Lang et al. (2017) suggest that rapid drying below physiological thresholds may promote acclimation and increase the invasive capacity of foodborne pathogens, as cells subjected to drying exhibited greater invasiveness than those not exposed. Thus, the way bacteria experience environmental perturbations in food matrices, as demonstrated in this study, may enhance their survival through the gastrointestinal tract and their ability to infect epithelial cells.

*Salmonella* coordinates the activation of metabolic pathways that support stress adaptation and regulate multiple genes, enhancing its adherence and invasiveness in host cells (Burgess et al. 2016; Feeney et al. 2014; Guo and Gross 2014; Lang et al. 2017; Rychlik and Barrow 2005; Shen and Fang 2012). Virulence factors, including pathogenicity islands, effector protein systems, virulence plasmids, and structures such as fimbriae and flagella, along with metabolic adaptations, enable the pathogen to survive the gastrointestinal tract and infect epithelial cells (dos Santos et al. 2019; Gruzdev, McClelland, et al. 2012; Gruzdev, Pinto, et al. 2012).

### 3.1 Pathogenicity Island and Type III Secretion System

Pathogenicity islands (SPIs) are chromosomal regions in *Salmonella* that encode proteins essential for host cell infection. Five SPIs have been identified, but this review focuses on the three most studied: SPI‐1, SPI‐2, and SPI‐3. SPI‐1 encodes effector proteins responsible for epithelial cell invasion, particularly the Type III secretion system (T3SS‐1), or “needle complex,” and also contributes to the initial formation of the *Salmonella*‐containing vacuole (SCV). SPI‐2 encodes T3SS‐2, which acts at later stages of infection to form *Salmonella*‐induced filaments (SIFs) and is critical for survival and replication within the SCV. SPI‐3 supports intra‐macrophage survival and growth, especially under low Mg^2^^+^ conditions (Fàbrega and Vila 2013; Fong and Wang 2016; Gerlach et al. 2007; Huang et al. 2007; Lang et al. 2017; Maserati et al. 2017; Mutz et al. 2020; Romero‐González et al. 2020; Rychlik and Barrow 2005).

During infection, osmolarity variations trigger the expression of *Salmonella* genes located in SPIS. This activation begins with signals from the macrophage environment, sensed by the EnvZ kinase, which, in turn, activates the response regulator OmpR (Huang et al. 2007; Lang et al. 2017; Rychlik and Barrow 2005). In *S*. Typhimurium, OmpR binds to SPIs 1, 2, and 4, as well as to genes controlled by phoP, and appears to enhance phoP expression through an unknown mechanism, whereas Hariharan et al. (2023) have already shown that deleting phoP reduced bacterial survival in *S*. Typhimurium mechanically stressed dried droplets model. The core OmpR regulon includes genes encoding surface‐associated organelles and proteins, suggesting that modulation of cell‐surface composition is a key function of this regulon under stress conditions (Quinn et al. 2014).

SPI‐1 expression is triggered by multiple environmental signals and regulated by the SPI‐1‐encoded proteins HilC and HilD. These, in turn, activate HilA, which drives the expression of SPI‐1 structural genes encoding the T3SS‐1 (Bajaj et al. 1995; Ellermeier et al. 2005; Quinn et al. 2014). This process also requires core‐genome regulators such as OmpR, Fis, and H‐NS (Dillon et al. 2010; Kelly et al. 2004; Lucchini et al. 2006; Osborne and Coombes 2011; Quinn et al. 2014). Notably, OmpR positively regulates hilC while repressing hilD expression (Cameron and Dorman 2012; Quinn et al. 2014).

HilA is the central transcriptional regulator of SPI‐1 in *Salmonella*, activating invasion genes directly at their promoters and indirectly by increasing InvF and InvA levels (Aviles et al. 2013). It also binds upstream of prgH to directly activate the prgHIJK operon, essential for injectisome assembly (Hu et al. 2019). Exposure of *S*. Typhimurium to osmotic stress, such as seawater with 4% salinity, enhanced adherence and invasion in Caco‐2 cells (Chakroun et al. 2018). Moreover, cells grown in Luria‐Bertani medium supplemented with 0.3 M NaCl expressed sopB, sopE2, and hilA at higher levels than those inside Caco‐2 cells.

Like HilA, OmpR binds within the prgH intergenic region and the prgHIJK operon, which encodes structural components of the SPI‐1 needle complex used to deliver bacterial effectors into host cells (Kubori et al. 1998; Quinn et al. 2014). Chakraborty and Kenney (2018) examined OmpR regulation by comparing the transcriptomes of an ompR mutant and wild‐type *S*. Typhimurium under acid, sucrose, and salt stress. They found that the type of osmolyte influenced the response, with salt stress producing the most differentially expressed genes (947), followed by sucrose (764) and acid (240). Remarkably, half of the most strongly induced genes (fold change >10) were SPI‐2 or SPI‐2‐coregulated, which support survival in the high osmolarity and low pH of macrophage vacuoles (Chakraborty et al. 2015). Although OmpR does not directly regulate most SPI‐2 genes beyond ssrA and ssrB (Chakraborty and Kenney 2018; Feng et al. 2004; X. Feng, Oropeza, Walthers, et al. 2003; Lee et al. 2000), many are directly controlled by SsrB (Chakraborty and Kenney 2018; Feng et al. 2004; Walthers et al. 2007, 2011).

The ssaHIJ SPI‐2 genes are required for *Salmonella*’s survival under desiccation (Mandal and Kwon 2017). Using small RNA sequencing, Barnhill et al. (2019) identified a candidate sRNA (sRNA1448803) predicted to regulate ssaL‐1, a T3SS protein also linked to desiccation tolerance (Mandal and Kwon 2017). Although this suggests a regulatory role for sRNA1448803, further experimental validation is still needed (Barnhill et al. 2019).

Maserati et al. (2018) performed a global transcriptomic analysis of *S*. Typhimurium at 0.11 aw and identified six virulence‐related genes differentially expressed (sscA, sseA, sopD, sseD, mgtC, and mviN). Among these, mgtC, part of the SPI‐3 mgtBC operon regulated by PhoP/PhoQ and involved in membrane potential, and mviN, required for peptidoglycan biosynthesis and virulence in mice, were upregulated (Rensing et al. 1997, 2000; Stenberg et al. 2005; Wu et al. 2005). The most critical genes for desiccation tolerance, however, were sopD and sseD, as deletion mutants showed reduced survival compared to the wild type. Although previously linked to virulence and infection, this was the first evidence connecting them to desiccation adaptation (Maserati et al. 2017).

SseD, a translocon protein encoded by the SPI‐2 T3SS within the sseABCD operon, works with chaperones to ensure proper folding, prevent aggregation, and support translocation of effector proteins across membranes (Cirillo et al. 1998; Duncan et al. 2015; Finka et al. 2015; Maserati et al. 2017; Nikolaus et al. 2001). Maserati et al. (2017) also found induction of sseA and sscA, which encode chaperonins for SseB/D and SseC, respectively, under 0.11 aw, suggesting the SPI‐2 translocon plays a key role in desiccation survival. SopD, originally identified as an SPI‐1 effector (Jones et al. 1998), is also deployed via the SPI‐2 injectisome (Brumell et al. 2003), remains expressed at later infection stages, and contributes to intracellular survival (Brumell et al. 2003; Jiang et al. 2004). Maserati et al. (2017) hypothesized that reduced desiccation tolerance in ΔsopD and ΔsseD mutants results from impaired SopD secretion. Supporting this, SopD is co‐secreted with SipA and other effectors (SopA, SopB, SopE, and SopE2) during host invasion (Raffatellu et al. 2005; Zhang et al. 2002), and SipA itself was upregulated under dry conditions, promoting actin filament stabilization for cell uptake (Maserati et al. 2018; Zhou et al. 1999).

Evidence also links desiccation with increased virulence in biofilm‐associated *Salmonella*. Aviles et al. (2013) showed that *S*. Tennessee biofilms stored in dry milk for 720 h expressed higher levels of virulence genes (sipC, invA, and hilA) than planktonic cells, particularly during the intestinal phase of in vitro digestion. These genes encode critical invasion functions, with hilA regulating multiple virulence pathways, invA encoding a T3SS component for penetration, and sipC producing an actin‐modifying effector. Prolonged drying also induced stress regulators such as RpoS and RpoE, both of which enhance survival and may further boost virulence inside the host (Lang et al. 2017; Li et al. 2015).

### 3.2 Virulence Plasmid, Fimbriae, and Flagella

Unlike the well‐studied virulence factors encoded by pathogenicity islands, little is known about the behavior of plasmid‐borne virulence genes under osmotic stress or desiccation. Although virulence plasmids vary across *Salmonella* serovars, all share a conserved 8 kb region carrying the spv locus, which includes the regulatory gene spvR and structural genes spvABCD. This locus supports survival and growth inside the host, as these genes remain silent during exponential in vitro growth but are rapidly induced upon entry into mammalian cells such as macrophages (Marcus et al. 2000; Mutz et al. 2020). In *S*. Typhimurium, spv transcription is controlled by RpoS (Mutz et al. 2020).

Transcriptomic data from dehydrated and water‐suspended *S*. Typhimurium revealed that, after 22 h of dehydration, four of seven downregulated genes were linked to virulence plasmid maintenance: parA, traN, trbH, and PSLT068 (Gruzdev, McClelland, et al. 2012). Normally induced under stress conditions resembling the host environment, nutrient limitation, high temperature, iron restriction, or low pH (Gruzdev, McClelland, et al. 2012; Guiney et al. 1995; Lucas and Lee 2000; Rychlik et al. 2006), their downregulation under drying may reflect physiological changes, possibly influenced by nutrient release from dead cells, creating conditions favorable to survival (Gruzdev, McClelland, et al. 2012).

Other host‐colonization genes were also regulated under desiccation. In *S*. Typhimurium cells equilibrated to 0.11 aw, genes linked to fimbrial operons (sthA, sthB, sthE, bcfC, and stiA) and flagellar biosynthesis (fliK, flhA, and fliJ) were upregulated, suggesting that structural adaptations support persistence in low‐moisture conditions (Maserati et al. 2017).

### 3.3 Metabolic Changes

Beyond activating virulence mechanisms during infection, *Salmonella* also adjusts its metabolism to survive within the host. Gruzdev et al. (2012) showed that dehydration induces the glyoxylate shunt genes aceA (isocitrate lyase) and aceB (malate synthase), a pathway typically triggered under glucose limitation (Fischer and Sauer 2003; Gruzdev, McClelland, et al. 2012). This shunt, also activated during anaerobic growth with hydrogen (Lamichhane‐Khadka et al. 2011) and required for persistence in chronic infection (Fang et al. 2005), diverts metabolites away from the TCA cycle, reducing NADH and thereby limiting ROS production (Gruzdev, McClelland, et al. 2012; Rui et al. 2010). Such activity may help protect *Salmonella* from oxidative radicals generated during desiccation (Potts 1994). The fnr gene, encoding an oxygen‐sensing regulator of motility, metabolism, and virulence, was also induced under dehydration (Fink et al. 2007; Gruzdev, McClelland, et al. 2012; Kang et al. 2005).

LPS, another key virulence determinant, supports motility, oxidative stress tolerance, and host invasion (Bogomolnaya et al. 2014; Choi and Groisman 2013; Jayeola et al. 2020; Thomsen et al. 2003; Toguchi et al. 2000). Genes involved in O‐antigen synthesis (rfbP, rfbN, rfbC, and rfbD) and core biosynthesis (rfaL, rfaJ, and rfaH) are critical for survival on pistachios (Jayeola et al. 2020; Schnaitman and Klenat 1993), and mutations in LPS genes reduce survival on abiotic surfaces under ambient conditions (Garmiri et al. 2008; Mandal and Kwon 2017).

Trehalose also emerges as a key link between osmoprotection and virulence. Pilonieta et al. (2012) found that ΔopuABCD mutants, unable to produce glycine betaine, compensated by increasing trehalose production, which enhanced resistance to stress in broth, mice, and macrophages. This suggests that the OpuABCD transport system may limit virulence by suppressing trehalose accumulation. Indeed, ΔopuA‐D strains were more resistant to salt, low pH, and oxidative stress and showed increased virulence in mice compared to wild type (Pilonieta et al. 2012).

## 4 Cross‐Protection

Low‐aw foods pose a particular risk because processing conditions may not fully eliminate *Salmonella* and can even expose cells to sublethal stresses that enhance persistence along the food chain (Fong and Wang 2016). Hurdle interventions are widely used to reduce contamination, but they may also promote cross‐protection, making control more challenging (Fong and Wang 2016). Under low aw, *Salmonella* often develops tolerance to other stresses, including biocides, ethanol, sodium hypochlorite, sodium chloride, and UV irradiation (Finn, Condell, et al. 2013; Fong and Wang 2016; Gruzdev et al. 2011; Gruzdev, Pinto, et al. 2012). This highlights the need to carefully validate intervention strategies (Fong and Wang 2016). Desiccation can trigger broad stress responses, with overlapping pathways that allow cross‐tolerance to multiple stresses (Bowman et al. 2015; Chen and Jiang 2017; Gruzdev et al. 2011; Kieboom et al. 2006). Barnhill et al. (2019) showed that many sRNAs expressed under desiccation were also differentially expressed under nutrient limitation, peroxide, bile, or NaCl shock (Kröger et al. 2012, 2013).

This cross‐protection effect is well illustrated by Choi et al. (2016), who sequentially exposed pak choi seeds inoculated with *E. coli* O157:H7 or *S*. Enterica to ClO_2_, drying, and dry heat. However, *E. coli* was inactivated, *S*. Enterica survived, and contaminated sprouts later reached infectious doses (>8.4 CFU/g). These findings confirm that sequential treatments must be optimized to fully inactivate pathogens on seeds. Desiccation‐adapted *Salmonella* also shows increased heat tolerance, with higher D‐values (time to kill 90% of cells at a given temperature) as aw decreases (Liu et al. 2018; Xu et al. 2019, 2020). Xu et al. (2020) reported that desiccated *Salmonella* Enteritidis survived 60 min at 80°C with <1.5 log reduction, whereas cells in broth were destroyed within 5 min. Similarly, Gruzdev et al. (2011) observed no significant reduction in desiccated *S*. Enterica after 1 h at 60°C, compared to a 3‐log drop in non‐desiccated cells. Structural protection underlies this tolerance: TEM studies showed that heat‐treated desiccated cells retained intact ultrastructure, unlike broth‐suspended cells, which displayed ribosome disruption, protein aggregation, and DNA damage (Xu et al. 2020).

Desiccation stress also induces molecular defenses. Heat shock proteins such as Hsp70, encoded by dnaK, and its cofactor grpE are consistently upregulated (Chen and Jiang 2017; Deng et al. 2012; Gruzdev, McClelland, et al. 2012; Gruzdev, Pinto, et al. 2012). These proteins, together with DnaJ, prevent aggregation and assist refolding of damaged proteins (Bateman et al. 2023). Other stress genes, including groEL, ibpA, ydaA, and sigma factors rpoH and rpoE, are also activated (Deng et al. 2012; Gruzdev, McClelland, et al. 2012). RpoH regulates cytoplasmic chaperones, whereas RpoE controls extra cytoplasmic stress responses and multiple pathways linked to heat, oxidative, osmotic, and starvation tolerance (Alba and Gross 2004; Gruzdev, McClelland, et al. 2012; Rhodius et al. 2005; Rowley et al. 2006). Notably, rpoE mutants show reduced dehydration tolerance (Gruzdev, McClelland, et al. 2012). RpoS is another key regulator: ΔrpoS mutants adapted to desiccation were less heat‐resistant than wild types, confirming its role in cross‐protection (Aviles et al. 2013; Chen and Jiang 2017).

Oxidative stress is another major consequence of desiccation. Membrane damage disrupts respiration, leading to ROS accumulation, lipid peroxidation, protein oxidation, and DNA lesions (França et al. 2007; García 2011; Lebre et al. 2017). *Salmonella* responds by upregulating GorA, a glutathione oxidoreductase, and Dps, a DNA‐binding ferritin‐like protein that prevents ROS‐driven DNA damage (Maserati et al. 2018; Michán et al. 1999; Zhao et al. 2002). Genes supporting Fe‐S cluster assembly (nifU, nifS, iscA, and sufD) and regulators like fnr are also induced, protecting respiration and long‐term survival (Gruzdev, McClelland, et al. 2012; Maserati et al. 2017). Preadaptation to desiccation further stabilizes membranes through conversion of unsaturated fatty acids into cyclopropane fatty acids (CFA), which resist ROS better than unsaturated lipids (Fang et al. 2020; Grogan et al. 1986; Zhang and Rock 2008). Trehalose also plays a protective role, preventing lipid peroxidation by forming stable complexes with unsaturated fatty acids (Fang et al. 2020; Oku et al. 2003).

Environmental and food matrix factors further reinforce protection. Biofilms increase heat resistance, with biofilm‐forming *Salmonella* Enteritidis in wheat flour survives higher temperatures than non‐biofilm strains (Maserati et al. 2018; Villa‐Rojas et al. 2017). Similarly, high‐fat, high‐protein foods like peanut butter shield cells against thermal inactivation (He et al. 2011; Maserati et al. 2018). Aviles et al. (2013) also showed that desiccation and biofilm state improved survival of *S*. Tennessee during simulated gastrointestinal passage, highlighting the potential link between stress adaptation and virulence. Altogether, these findings illustrate how *Salmonella* adapts under low‐aw conditions, gaining cross‐tolerance that complicates control and increases risks for food safety.

## 5 Gene Expression After Cell Rehydration

Several studies show that rehydration after osmotic stress can trigger the expression of genes that promote metabolic recovery and cell multiplication (Chen and Jiang 2017; Finn, Händler, et al. 2013; Maserati et al. 2018). However, this recovery is often slow, as cells need time to readjust their processes, particularly after long‐term stress. A sudden return to high aw can be lethal, but when cells survive, they gradually restore metabolism and resume growth. Rehydrated cells undergo major adjustments in protein expression: fatty acid catabolism decreases, phosphate transport increases to support nucleic acid synthesis, and motility genes return to normal activity (Finn, Händler, et al. 2013). Maserati et al. (2018) found higher levels of flagellar proteins (FlgEFGH), membrane proteins, and export systems (SecF, SecD, and Bam complex), along with stress response proteins, indicating that rehydration itself can act as a stressor.

Correct assembly and transport of outer membrane proteins are critical for membrane integrity and cell division. In moist samples, proteins of the Bam complex, YaeT (BamA), YfgL (BamB), and YfiO (BamD), were more abundant, underscoring their role in membrane stability under high aw (Fardini et al. 2007, 2009; Maserati et al. 2018). Likewise, HtpX, a protease that degrades misfolded proteins, was found in moist cells, suggesting a need for quality control during membrane recovery (Maserati et al. 2018; Sakoh et al. 2005; Shimohata et al. 2002). TreA, which hydrolyzes trehalose into glucose, was also more abundant after long‐term desiccation, highlighting its role in mobilizing energy from accumulated trehalose during rehydration (Finn, Händler, et al. 2013; Maserati et al. 2018; Mutz et al. 2020; Strom and Kaasen 1993).

Flagellar expression, suppressed during desiccation to conserve energy, resumed under moist conditions (Cytryn et al. 2007; Kocharunchitt et al. 2014; Lebre et al. 2017; Maserati et al. 2018). Ribosomal proteins, more abundant in dry than moist samples, may be degraded to recycle amino acids for recovery (Maserati et al. 2018). Stress response proteins such as SspA, GorA, and Dps were also detected in moist samples, suggesting that even in favorable conditions, cells prepare against oxidative damage, possibly linked to increased ROS production during metabolic reactivation (Hansen et al. 2005; Maserati et al. 2018). The upregulation of rpoE under both desiccation and rehydration further supports its central role in stress adaptation (Finn, Händler, et al. 2013; Maserati et al. 2018).

Rehydration is not always beneficial. Maserati et al. (2017) observed that desiccated *Salmonella* exposed to aw 1.0 showed loss of turgidity, wrinkled membranes, and cell debris, though some cells regained viability, possibly by scavenging nutrients from lysed cells or EPS residues (Billi and Potts 2002; Colgan et al. 2016; Maserati et al. 2017). Interestingly, ΔsseD mutants failed to recover, suggesting effector proteins are also involved in post‐desiccation survival. Rehydration also reverses desiccation‐acquired thermal tolerance. Chen and Jiang (2017) demonstrated that thermal resistance induced under desiccation diminished upon rehydration, though cells remained more resistant than non‐stressed controls. Gruzdev et al. (2011) reported that this tolerance persists only briefly, whereas Maserati et al. (2018) linked the loss to protein expression changes during rehydration. Altogether, these findings show that although preadaptation to dryness boosts thermal tolerance, rehydration reshapes metabolism in ways that may compromise cross‐protection.

## 6 VBNC State

*Salmonella* poses a major food safety risk because sublethal stress, food composition, and cell adaptations, such as filamentation, can lower its already low infectious dose (Akhtar et al. 2014; Aviles et al. 2013; Lang et al. 2017). High‐fat or high‐protein foods protect cells during gastric passage, enabling infection with as few as 10–100 cells (Birk et al. 2012, 2016; Mutz et al. 2020; Teunis et al. 2010). Filamentous cells formed under stress may be underestimated by culture methods but can separate after ingestion, increasing the risk of disease (Mattick et al. 2003).

Similar underestimations occur with cells in the VBNC state, a dormant condition in which bacteria lose the ability to grow on conventional media but retain metabolic activity (Pienaar et al. 2016). This state is usually triggered by environmental stress and is distinct from sublethally injured cells because VBNC cells preserve membrane integrity, though they display reduced respiration and gene transcription (Kusumoto et al. 2012; Oliver 2005). Importantly, VBNC cells can maintain virulence and have been associated with foodborne illness (Asakura et al. 2002; Li et al. 2017; Makino et al. 2000; Salive et al. 2020). Entry into the VBNC state can be induced by nutrient limitation, shifts in temperature, oxygen, or pH, high salinity, osmolarity, exposure to heavy metals, preservatives, visible or UV light, or combinations of these stresses (Asakura et al. 2008; Besnard et al. 2002; Cunningham et al. 2009; Ghezzi and Steck 1999; Gourmelon et al. 1994; Oliveira et al. 2021; Quirós et al. 2009; Wong and Liu 2008).

Although many conditions are known to induce VBNC, little is reported on *Salmonella* entering this state under desiccation or osmotic stress in food systems. As foods are frequently exposed to adverse conditions, from farm to processing, low‐aw treatments could drive microbiota into VBNC as a survival strategy (Salive et al. 2020). Given *Salmonella*’s prevalence in LMFs, its presence in the VBNC state cannot be excluded, especially since conventional microbiological methods fail to detect such cells. This could explain outbreaks where only low numbers of *Salmonella* were recovered from implicated products (Asakura et al. 2002; Danyluk et al. 2007; Gill et al. 1983; Gruzdev, Pinto, et al. 2012; Jayeola et al. 2022). However, direct experimental evidence for VBNC induction in *Salmonella* contaminating LMFs is still lacking (Jayeola et al. 2022).

### 6.1 Entrance VBNC State Due Osmotic Stress

Studies on *Salmonella* under osmotic stress generally report a slow loss of culturability, known as long‐term induction, whereas cells remain viable (Oliveira et al. 2021). In vitro research models typically use salts and sugars to adjust the aw of experimental matrices. Saline exposure induces stress in *Salmonella* by creating an osmotic imbalance, which the bacterium counteracts through rapid genetic adaptations such as the upregulation of stress‐response genes (rpoS, proX, proW, otsA, OmpR‐EnvZ, and PhoPQ) and physiological responses, including the accumulation of osmoprotectants (e.g., uptake or synthesis of K^+^, trehalose, proline, and betaine) (Gong et al. 2025; Zhang et al. 2024).

High concentrations of sugars, in turn, particularly glucose, can cause intracellular accumulation of phosphorylated sugars (such as glucose‐6‐phosphate) or trigger a reduction in sugar uptake, a process regulated by SgrS and SgrT, which limit further sugar import in *Salmonella* (Bobrovskyy and Vanderpool 2014; Palmer et al. 2015).

Most experiments use saturated saline solutions to impose sublethal stress (Oliveira et al. 2021; Rodrigues et al. 2015; Salive et al. 2020). For instance, *S*. Typhimurium exposed to nutritional, osmotic, and cold stresses in Butterfield's phosphate solution with 1.2 M NaCl at 4°C entered the VBNC state after 135 days, though flow cytometry confirmed cell viability (Oliveira et al. 2021). Similarly, *Salmonella* Enteritidis and *S*. Typhimurium lost culturability after 121 days under comparable conditions, whereas more than 80% of cells remained viable (Salive et al. 2020). Song and Lee (2021) found that *Salmonella* Enteritidis in lactic acid‐adjusted LB medium with 5%, 10%, or 30% NaCl survived 91, 49, and 28 days, respectively, but over 73% of cells remained alive after 84 days in 10% NaCl, as confirmed by SYTO9/PI staining.

Other studies support that VBNC induction requires prolonged exposure to sublethal stress. Li et al. (2020) showed that *S*. Enterica entered the VBNC state under combinations of starvation, acid, and high NaCl, but culturability was lost within 3 days under severe starvation with high salt, leaving no viable cells. Conversely, under acidic conditions, VBNC induction took more than 30 days, suggesting that nutrients or acid delay entry into this state. Rodrigues et al. (2015) reported that adding 0.6 M NaCl to oligotrophic medium reduced culturability in *Salmonella* Enteritidis PT4 578 strains, shortening induction time from 180 to 140 days and slightly lowering the percentage of viable cells.

Sugars can also trigger VBNC entry. Jayeola et al. (2022) observed that high fructose levels in apple juice accelerated the loss of culturability despite an aw >0.85, whereas viability remained unaffected. However, relatively few studies have examined sugar‐induced osmotic stress compared with salt‐based systems.

### 6.2 Entrance VBNC State Due Desiccation Stress

*Salmonella* can also enter the VBNC state under desiccation stress. Most studies induce this state through dehydration combined with poor nutritional conditions, often without using a food matrix (Gruzdev, McClelland, et al. 2012; Gruzdev, Pinto, et al. 2012; Morishige et al. 2017). Jayeola et al. (2022) inoculated a *Salmonella* cocktail onto dried apples, dried the samples at 25°C, and stored them at 4°C and 25°C. At 25°C, *Salmonella* counts declined progressively until becoming undetectable after ∼46 days, even with enrichment, whereas at 4°C, cells were detectable up to 82 days. This showed that storage temperature influences the time required to reach the VBNC state. Using fluorescence microscopy, the authors confirmed that 56%–85% of cells remained viable despite culturability loss. These findings suggest that the combined stressors of dried fruit, including high solute concentration, low pH, and desiccation, rather than low‐aw or acid stress alone, can induce large populations of VBNC *Salmonella* (Jayeola et al. 2022).

### 6.3 Morphology and Physiology Changes in *Salmonella* VBNC Under Low aw

The time required for *Salmonella* to enter the VBNC state and undergo morphological and physiological changes depends on factors such as stress type and intensity, exposure duration, cell age, and population density (Oliveira et al. 2021; Salive et al. 2020). These variables influence both the degree of resistance achieved and the nature of adaptive cellular responses. For example, *Salmonella* Enteritidis PT4 578 ΔrelA and ΔrelAΔspoT mutants exposed to osmotic, nutritional, and cold stress for 140 days entered the VBNC state, showing reduced diameter, volume, and length, with a bacillary‐to‐coccoid shift. Atomic force microscopy revealed a 71% reduction in volume, from ∼0.63 µm^3^ in non‐stressed cells to ∼0.18 µm^3^ in VBNC cells (Rodrigues et al. 2015). Similarly, *S*. Typhimurium ATCC 14028 exposed to 1.2 M NaCl developed roughened surfaces and coccoid morphology (Salive et al. 2020), a change absent in cells rapidly driven to VBNC by acidic or oxidative stress. Such coccoid transformations have also been reported in other studies (Gupte et al. 2003; Zeng et al. 2013). Reducing cell size under stress is considered a survival strategy, minimizing maintenance needs and enhancing nutrient uptake through an increased surface‐to‐volume ratio (Jiang and Chai 1996; Salive et al. 2020).

These morphological changes appear linked to the downregulation of the mreB gene, which helps maintain rod shape. In *Salmonella*, MreB, MreC, and MreD proteins encoded by the mre operon determine morphology (Doble et al. 2012). Under nutritional and cold stress, mreB expression fell significantly after 25 days (Rodrigues et al. 2015). Similar reductions in mreB expression under nutrient limitation were also reported in *Vibrio parahaemolyticus*, *Helicobacter pylori*, and *B. subtilis* (Chiu et al. 2008; Eymann et al. 2002; Thompson et al. 2003). However, not all stressed cells become coccoid. After exposure to 10% NaCl for 28 days, *Salmonella* Enteritidis cells elongated and exhibited cytoplasmic density loss, membrane damage, and periplasmic gaps without yet entering the VBNC state (Song and Lee 2021). Similarly, oxidative agents such as peracetic acid and hydrogen peroxide induced rapid VBNC entry but preserved bacillary shape (Salive et al. 2020). These findings suggest induction time and stress type determine whether VBNC cells adopt coccoid or bacillary forms.

Despite these insights, knowledge of gene expression in VBNC cells remains limited. Most transcriptomic studies are conducted when part of the population remains culturable, potentially masking true VBNC responses, and extended desiccation complicates detection due to RNA degradation (Finn, Händler, et al. 2013). Deng et al. (2012) found that less than 5% of the *Salmonella* genome was transcribed during desiccation in peanut oil (aw 0.3), suggesting cells largely remain physiologically dormant. Yet, molecular tools, such as RT‐qPCR, have provided useful clues. In dry milk powder, *S*. Tennessee biofilms survived better than planktonic cells, though qPCR showed similar genome counts, suggesting planktonic cells had shifted into VBNC (Aviles et al. 2013). Likewise, biofilm *S*. Typhimurium stored in powdered infant formula showed a stronger tendency to enter VBNC, with rpoS significantly upregulated in both biofilm and planktonic populations (Chen et al. 2021).

RpoS appears central to VBNC induction. Kusumoto et al. (2012) found that *Salmonella* Dublin, *Salmonella* Oranienburg, and *S*. Typhimurium LT2 entered VBNC under 7% NaCl in relation to their ability to produce RpoS. *S*. Dublin, with higher RpoS levels, entered VBNC later, whereas RpoS rapidly decreased in *S*. Typhimurium LT2 and *S*. Oranienburg. Mutants deficient in rpoS entered VBNC more quickly, consistent with earlier observations in *E. coli* and *Salmonella* (Boaretti et al. 2003; Munro et al. 1995). Thus, RpoS likely helps delay VBNC entry and supports culturability maintenance.

Overall, the molecular basis of VBNC in *Salmonella* remains poorly understood, but insights into morphological adaptation, mreB downregulation, and RpoS regulation highlight key pathways. Further research into these mechanisms may not only clarify VBNC physiology but also enable the development of novel detection strategies to improve food safety (Li et al. 2014).

### 6.4 How to Detect VBNC Cells?

The ability of *Salmonella* to enter the VBNC state in LMF poses major food safety risks, as these cells are not detected by standard culture‐based methods, leading to false negatives and the possible release of contaminated products (Jayeola et al. 2022; Li et al. 2020). This risk is particularly critical since LMF are ready‐to‐eat, often consumed without further processing, and VBNC cells can retain virulence (Salive et al. 2020). Their presence may also explain the low infectious doses observed in outbreaks, as conventional estimates based on CFU counts underestimate the actual number of viable cells (Aviles et al. 2013). Although *Salmonella* in the VBNC state may not grow on solid media, culturability can persist longer in liquid media (Oliveira et al. 2021; Salive et al. 2020), and methodological adjustments, such as using pour plates with larger aliquots, can improve recovery and detection. As an alternative to direct plating, the most probable number (MPN) method can be employed. This approach allows the use of a nonselective enrichment medium to help recover injured cells, followed by transferring tubes that show growth to a selective medium for confirmation of the target organism (Abd et al. 2017). The MPN technique is recognized for its high sensitivity, suitability for complex food matrices, and statistically based quantification, making it especially useful for detecting *Salmonella* in LMF where contamination levels are typically low (Chakroun et al. 2018; Nascimento et al. 2013).

**FIGURE 4:**
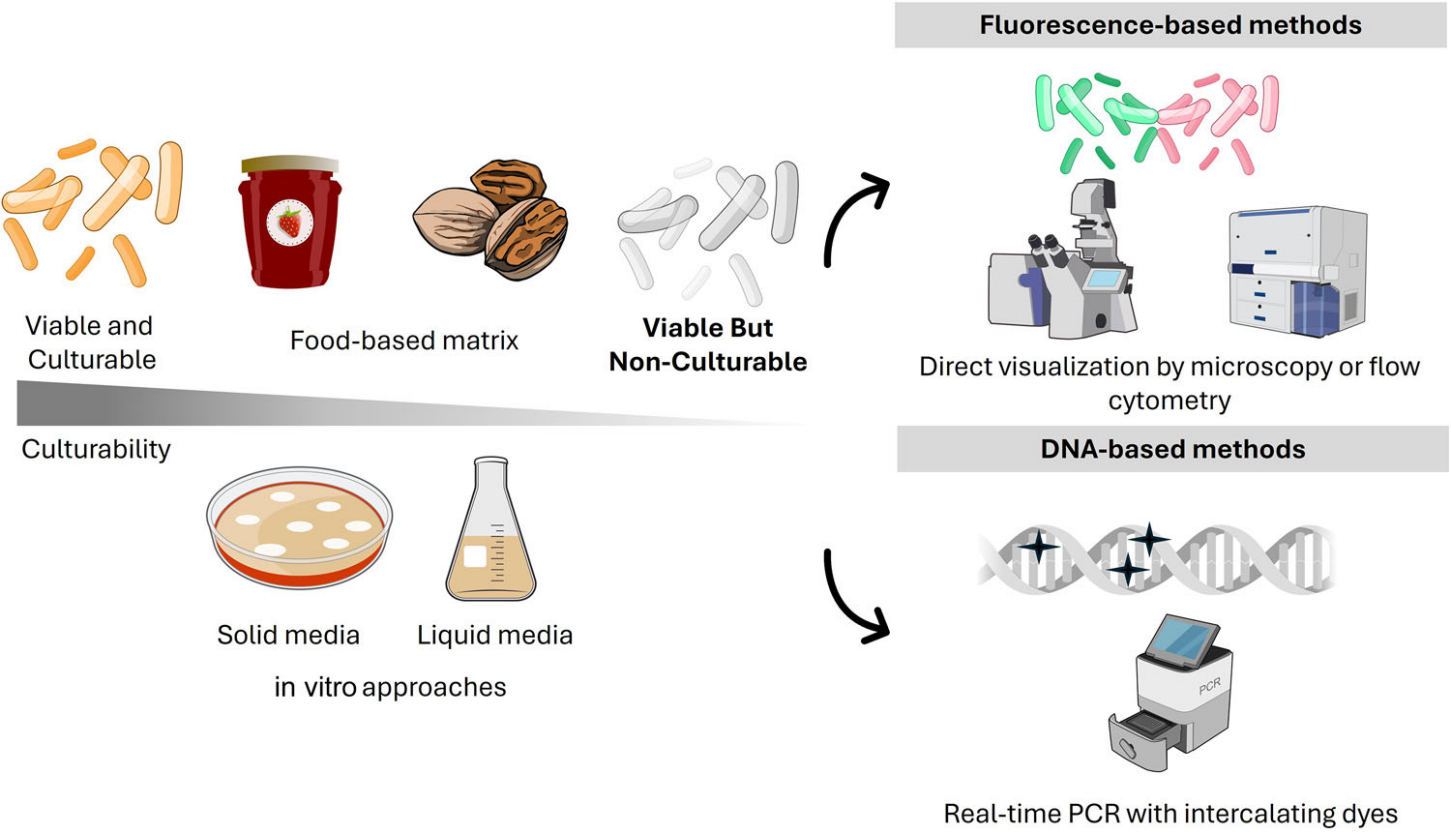
Comparative culturability and advanced detection methods in VBNC *Salmonella*. Image credit: The figure was drawn by using modified pictures from NIH BioArt Source under a Public Domain License and Pixabay.

Other approaches have been developed to detect VBNC cells (Figure 4), though most remain unvalidated against gold standard methods. Two widely used strategies are (i) direct detection by fluorescence microscopy or flow cytometry with viability markers and (ii) real‐time PCR with DNA intercalators to differentiate live and dead cells. The Live/Dead kit, commonly applied to assess membrane integrity, distinguishes intact (green‐stained) from compromised (red‐stained) cells (Dinu and Bach 2011; Gao et al. 2024; Hoefman et al. 2012; Lindbäck et al. 2010; Postnikova et al. 2015; Salive et al. 2020; Zhao et al. 2013). However, Syto 9 can sometimes be excluded by viable cells, and fluorescence microscopy is time‐consuming, limiting its practicality. Flow cytometry combined with these dyes offers faster, more sensitive analysis and has proven efficient in detecting VBNC cells under osmotic stress (Rodrigues et al. 2015; Salive et al. 2020).

In recent years, PMA–PCR methods have emerged as promising tools, as PMA treatment eliminates signals from dead cells, improving specificity. Li et al. (2020) successfully applied PMA–PCR to detect *S*. Enterica VBNC cells in a simulated food system with a sensitivity of 10^5^ CFU/mL, suggesting potential for broader application across food matrices (Liu et al. 2017; Ma et al. 2017; Miao et al. 2017a, 2017b, 2018; Wang et al. 2011, 2019). mRNA‐based assays have also been proposed, because mRNA is highly unstable and degrades rapidly, making its presence a marker of cell viability (Del Mar Lleo et al. 2000; Sheridan et al. 1998). However, short mRNA half‐lives can lead to false negatives. Advancing VBNC detection will depend on better understanding the mechanisms by which cells exit dormancy, which could guide the optimization of current methods.

### 6.5 Resuscitation of VBNC Cells

The resuscitation of *Salmonella* from the VBNC state was first described in *Salmonella* Enteritidis in 1984 (Roszak et al. 1984). This transition, termed “resuscitation,” refers to the recovery of culturability under favorable conditions, such as within the host intestine (Del Mar Lleo’ et al. 1998; Kusumoto et al. 2012; Lleò et al. 2001; Oliver 2005; Oliver and Bockian 1995; Pan and Ren 2022). Pathogens in the VBNC state retain virulence factors and may resume pathogenicity upon recovery (Chen et al. 2021; Zeng et al. 2013), making their presence in food a public health concern (Kusumoto et al. 2012; Oliver 2005). The stimuli that promote resuscitation remain poorly understood, though stress type, duration, and cell age all seem influential (Ayrapetyan and Oliver 2016; Zhao et al. 2017). Reported triggers include temperature changes (Ayrapetyan and Oliver 2016; Zhao et al. 2017), coculture with eukaryotic cells (Senoh et al. 2010), and exposure to compounds such as sodium pyruvate (Morishige et al. 2013), catalase (Morishige et al. 2017), or quorum sensing autoinducers (Ayrapetyan et al. 2014; Pan and Ren 2022).

Pyruvate is one of the most studied resuscitation agents, acting as an antioxidant that scavenges reactive oxygen species and prevents lipid peroxidation (Constantopoulos and Barranger 1984; Göing and Jung 2021; Mizunoe et al. 1999; O'donnell‐Tormey et al. 1987; Varma et al. 2003; Woo et al. 2004). Enzymes in pyruvate metabolism are strongly upregulated in VBNC cells, and pyruvate may serve as a preferred carbon source in starving bacteria because it bypasses phosphorylation steps required for glucose utilization (Göing and Jung 2021). In *Salmonella* Enteritidis, VBNC cells stressed with H_2_O_2_ regained culturability in minimal medium supplemented with ≥0.3 mM sodium pyruvate (Morishige et al. 2013). Similar antioxidant effects are observed in mammalian systems, where pyruvate secretion helps neutralize reactive oxygen species (Varma et al. 2003; Yin et al. 2016). Although not yet tested for desiccation‐ or osmotic‐induced *Salmonella* VBNC, pyruvate may promote recovery under these conditions.

Catalase has also been identified as a resuscitation trigger. In *Salmonella* Enteritidis exposed to desiccation under nutrient‐poor, low‐temperature conditions, catalase addition restored culturability in a dose‐dependent manner (Morishige et al. 2017). Like pyruvate, catalase neutralizes H_2_O_2_ and reverses ROS‐mediated damage, but its effectiveness appears limited when VBNC cells have adapted to long‐term stress, maintaining low ROS levels (Oliveira et al. 2021; Salive et al. 2020). Quorum sensing autoinducers (AHLs, oligopeptides, butyrolactones, and AI‐2 family signals) can also promote resuscitation by coordinating stress adaptation pathways, including catalase induction (Abisado et al. 2018; Hassett et al. 1999; Liao et al. 2019; Pan and Ren 2022; Waters and Bassler 2005). In *S*. Typhimurium, quorum sensing signals secreted during coculture restored growth in cells previously unculturable after extended stress (Reissbrodt et al. 2002).

Another resuscitation mechanism involves resuscitation‐promoting factors (RPFs), proteins structurally similar to lysozymes that hydrolyze peptidoglycan and trigger cell wall remodeling. In *S*. Oranienburg, RPFs promoted recovery from osmotic stress‐induced VBNC (Göing and Jung 2021; Pan and Ren 2022; Panutdaporn et al. 2006). These proteins may act by producing peptidoglycan fragments that serve as signaling molecules, though the exact mechanism remains unclear (Mukamolova et al. 2006; Nikitushkin et al. 2015). Inhibiting RPF activity with antibodies blocked resuscitation, suggesting their role as signal molecules (Panutdaporn et al. 2006). YeaZ, a protease‐like protein conserved across *Salmonella* and *E. coli*, also supports VBNC recovery, though its precise mechanism is less understood (Aydin et al. 2011; Handford et al. 2009; Pan and Ren 2022; Vecchietti et al. 2016).

As host environments can provide such stimuli, VBNC *Salmonella* surviving in foods may regain pathogenicity upon reaching the intestine, where they can resume virulence programs and cause infection. Their low metabolic activity also confers resistance to antibiotics, complicating treatment (Cunningham et al. 2009; Dinu and Bach 2011; Nicolò et al. 2011). Moreover, persistence in the VBNC state enables survival through cleaning and disinfection, raising concerns for food industry sanitation. Thus, clarifying the environmental factors and molecular mechanisms governing VBNC induction and resuscitation in *Salmonella* is crucial for developing improved detection and control strategies. Finally, it remains debated whether the VBNC state represents a true physiological adaptation or reflects limitations in our ability to replicate bacterial growth requirements in dormant cells (Pan and Ren 2022).

## 7 Conclusion

Our understanding of how *Salmonella* behaves under stress, particularly under low aw, remains limited. In industrial environments, LMF such as peanut products, spices, powdered ingredients, chocolate, and nuts frequently expose *Salmonella* to osmotic and desiccation stresses. These conditions induce physiological adaptations that could enhance its environmental persistence, induce cross‐protection against heat and chemicals, and complicate pathogen removal from equipment surfaces due to biofilm formation. In practical terms, these stress responses also hinder effective detection. *Salmonella*’s cells exposed to desiccation or osmotic stress may enter the VBNC state, leading to false negatives during routine monitoring and environmental verification. This directly affects hazard assessment and sanitation validation because stressed cells, although undetectable, remain capable of resuscitation under favorable conditions. In fact, these phenotypes likewise complicate outbreak investigations. Expanding knowledge on these adaptive mechanisms is therefore essential not only for refining cultivation techniques for stressed cells but also for developing more robust industrial interventions. Improved detection methods, tailored sanitation protocols for low‐aw environments, and cleaning strategies that consider stress‐induced cross‐protection can substantially enhance pathogen control. Ultimately, advances in this field will support the design of more effective preventive measures in processing plants, reduce the persistence of *Salmonella* in dry facilities, and strengthen the accuracy of outbreak characterization, contributing directly to improved public health outcomes.

## Author Contributions

**Mayara Messias Oliveira**: conceptualization, investigation, writing – original draft, writing – review and editing, visualization, validation, methodology, formal analysis, data curation. **Dionisio P. Amorim‐Neto**: writing – original draft, writing – review and editing, visualization, validation, resources. **Anderson S. Sant'Ana**: conceptualization, funding acquisition, writing – original draft, writing – review and editing, visualization, methodology, validation, project administration, supervision, resources.

## Conflicts of Interest

The authors declare no conflicts of interest.

## References

[crf370439-bib-0001] Abd, S. J. , C. M. H. Ferstl , and W. Ocasio . 2017. “Techniques to Determine Thermal Inactivation Kinetics for Pathogenic Bacteria and Their Surrogate Organisms in Low‐Moisture Foods.” In Control of Salmonella and Other Bacterial Pathogens in Low‐Moisture Foods, edited by R. Podolak and D. G. Black , 197–217. John Wiley & Sons Ltd. 10.1002/9781119071051.ch9.

[crf370439-bib-0315] Abdelhamid, A. G. , and A. E. Yousef . 2020. “Collateral Adaptive Responses Induced by Desiccation Stress in Salmonella enterica.” LWT 133: 110089. 10.1016/j.lwt.2020.110089.

[crf370439-bib-0002] Abisado, R. G. , S. Benomar , J. R. Klaus , A. A. Dandekar , and J. R. Chandler . 2018. “Bacterial Quorum Sensing and Microbial Community Interactions.” MBio 9, no. 3: e02331‐17. 10.1128/mbio.02331-17. 29789364 PMC5964356

[crf370439-bib-0003] Akhtar, S. , M. R. Sarker , and A. Hossain . 2014. “Microbiological Food Safety: A Dilemma of Developing Societies.” Critical Reviews in Microbiology 40, no. 4: 348–359. 10.3109/1040841X.2012.742036. 23173983

[crf370439-bib-0004] Alba, B. M. , and C. A. Gross . 2004. “Regulation of the *Escherichia coli* σE‐Dependent Envelope Stress Response.” Molecular Microbiology 52, no. 3: 613–619. 10.1111/J.1365-2958.2003.03982.X. 15101969

[crf370439-bib-0005] Albertorio, F. , V. A. Chapa , X. Chen , A. J. Diaz , and P. S. Cremer . 2007. “The α,α‐(1→1) Linkage of Trehalose Is Key to Anhydrobiotic Preservation.” Journal of the American Chemical Society 129, no. 34: 10567–10574. 10.1021/ja0731266. 17676844 PMC2551324

[crf370439-bib-0006] Alp, D. , and Ö. Bulantekin . 2021. “The Microbiological Quality of Various Foods Dried by Applying Different Drying Methods: A Review.” European Food Research and Technology 247, no. 6: 1333–1343. 10.1007/s00217-021-03731-z. 33824622 PMC8017434

[crf370439-bib-0007] Altendorf, K. , I. R. Booth , J. Gralla , J.‐C. Greie , A. Z. Rosenthal , and J. M. Wood . 2009. “Osmotic Stress.” EcoSal Plus 3, no. 2: 1–42. 10.1128/ecosalplus.5.4.5. 26443764

[crf370439-bib-0008] Aryal, M. , and P. M. Muriana . 2019. “Efficacy of Commercial Sanitizers Used in Food Processing Facilities for Inactivation of *Listeria monocytogenes*, *E. Coli* O157:H7, and *Salmonella* Biofilms.” Foods 8, no. 12: 639. 10.3390/foods8120639. 31817159 PMC6963748

[crf370439-bib-0009] Asakura, H. , K. Kawamoto , Y. Haishima , S. Igimi , S. Yamamoto , and S. I. Makino . 2008. “Differential Expression of the Outer Membrane Protein W (OmpW) Stress Response in Enterohemorrhagic *Escherichia coli* O157:H7 Corresponds to the Viable but Non‐Culturable State.” Research in Microbiology 159, no. 9–10: 709–717. 10.1016/J.RESMIC.2008.08.005. 18824229

[crf370439-bib-0010] Asakura, H. , S. Makino , T. Takagi , et al. 2002. “Passage in Mice Causes a Change in the Ability of *Salmonella enterica* Serovar Oranienburg to Survive NaCl Osmotic Stress: Resuscitation From the Viable but Non‐Culturable State.” FEMS Microbiology Letters 212, no. 1: 87–93. 10.1111/J.1574-6968.2002.TB11249.X. 12076792

[crf370439-bib-0011] Aviles, B. , C. Klotz , J. Eifert , R. Williams , and M. Ponder . 2013. “Biofilms Promote Survival and Virulence of *Salmonella enterica* Sv. Tennessee During Prolonged Dry Storage and After Passage Through an in Vitro Digestion System.” International Journal of Food Microbiology 162, no. 3: 252–259. 10.1016/J.IJFOODMICRO.2013.01.026. 23454816

[crf370439-bib-0012] Aydin, I. , Y. Saijo‐Hamano , K. Namba , C. Thomas , and A. Roujeinikova . 2011. “Structural Analysis of the Essential Resuscitation Promoting Factor YeaZ Suggests a Mechanism of Nucleotide Regulation Through Dimer Reorganization.” PLoS ONE 6, no. 8: e23245. 10.1371/JOURNAL.PONE.0023245. 21858042 PMC3157347

[crf370439-bib-0013] Ayrapetyan, M. , and J. D. Oliver . 2016. “The Viable but Non‐Culturable State and Its Relevance in Food Safety.” Current Opinion in Food Science 8: 127–133. 10.1016/J.COFS.2016.04.010.

[crf370439-bib-0014] Ayrapetyan, M. , T. C. Williams , and J. D. Oliver . 2014. “Interspecific Quorum Sensing Mediates the Resuscitation of Viable but Nonculturable Vibrios.” Applied and Environmental Microbiology 80, no. 8: 2478–2483. 10.1128/AEM.00080-14. 24509922 PMC3993182

[crf370439-bib-0015] Bajaj, V. , C. Hwang , and C. A. Lee . 1995. “hilA Is a Novel ompR/toxR Family Member That Activates the Expression of *Salmonella typhimurium* Invasion Genes.” Molecular Microbiology 18, no. 4: 715–727. 10.1111/j.1365-2958.1995.mmi_18040715.x. 8817493

[crf370439-bib-0016] Balaji, B. , K. O'Connor , J. R. Lucas , J. M. Anderson , and L. N. Csonka . 2005. “Timing of Induction of Osmotically Controlled Genes in *Salmonella enterica* Serovar Typhimurium, Determined With Quantitative Real‐Time Reverse Transcription‐PCR.” Applied and Environmental Microbiology 71, no. 12: 8273–8283. 10.1128/AEM.71.12.8273-8283.2005. 16332813 PMC1317391

[crf370439-bib-0017] Ballal, A. , B. Basu , and S. K. Apte . 2007. “The Kdp‐ATPase System and Its Regulation.” Journal of Biosciences 32, no. 3: 559–568. 10.1007/s12038-007-0055-7. 17536175

[crf370439-bib-0018] Barbosa‐Cánovas, G. V. , J. J. Fernández‐Molina , S. M. Alzamora , M. S. Tapia , A. López‐Malo , and J. W. Chanes . 2003. Handling and Preservation of Fruits and Vegetables by Combined Methods for Rural Areas: Technical Manual (FAO Agricultural Services Bulletin 149). Food And Agriculture Organization Of The United Nations.

[crf370439-bib-0019] Barnhill, E. C. , A. Crucello , D. Houserova , et al. 2019. “Characterization of Novel Small RNAs (sRNAs) Contributing to the Desiccation Response of *Salmonella enterica* Serovar Typhimurium.” RNA Biology 16, no. 11: 1643–1657. 10.1080/15476286.2019.1653680. 31390935 PMC6779410

[crf370439-bib-0322] Bateman, A. , M. Martin , S. Orchard , et al. 2023. “UniProt: the Universal Protein Knowledgebase in 2023.” Nucleic Acids Research 51, no. D1: D523–D531. 10.1093/nar/gkac1052. 36408920 PMC9825514

[crf370439-bib-0020] Belitz, H.‐D. , and W. Grosch . 2013. Food Chemistry. 2nd ed.. Springer Science & Business Media.

[crf370439-bib-0021] Bendezú, F. O. , and P. A. J. De Boer . 2008. “Conditional Lethality, Division Defects, Membrane Involution, and Endocytosis in Mre and Mrd Shape Mutants of *Escherichia coli* .” Journal of Bacteriology 190, no. 5: 1792–1811. 10.1128/jb.01322-07. 17993535 PMC2258658

[crf370439-bib-0022] Beney, L. , Y. Mille , and P. Gervais . 2004. “Death of *Escherichia coli* During Rapid and Severe Dehydration Is Related to Lipid Phase Transition.” Applied Microbiology and Biotechnology 65, no. 4: 457–464. 10.1007/s00253-004-1574-x. 15095024

[crf370439-bib-0023] Besnard, V. , M. Federighi , E. Declerq , F. Jugiau , and J. M. Cappelier . 2002. “Environmental and Physico‐Chemical Factors Induce VBNC State in *Listeria monocytogenes* .” Veterinary Research 33, no. 4: 359–370. 10.1051/VETRES:2002022. 12199363

[crf370439-bib-0024] Beuchat, L. R. , E. Komitopoulou , H. Beckers , et al. 2013. “Low–Water Activity Foods: Increased Concern as Vehicles of Foodborne Pathogens.” Journal of Food Protection 76, no. 1: 150–172. 10.4315/0362-028X.JFP-12-211. 23317872

[crf370439-bib-0025] Billi, D. , and M. Potts . 2002. “Life and Death of Dried Prokaryotes.” Research in Microbiology 153, no. 1: 7–12. 10.1016/S0923-2508(01)01279-7. 11881900

[crf370439-bib-0026] Birk, T. , S. Henriksen , K. Müller , T. B. Hansen , and S. Aabo . 2016. “Growth Potential of Exponential‐ and Stationary‐Phase *Salmonella typhimurium* During Sausage Fermentation.” Meat Science 121: 342–349. 10.1016/J.MEATSCI.2015.08.012. 27423056

[crf370439-bib-0027] Birk, T. , K. Kristensen , A. Harboe , et al. 2012. “Dietary Proteins Extend the Survival of *Salmonella* Dublin in a Gastric Acid Environment.” Journal of Food Protection 75, no. 2: 353–358. 10.4315/0362-028X.JFP-11-132. 22289597

[crf370439-bib-0028] Boaretti, M. , M. D. M. Lleò , B. Bonato , C. Signoretto , and P. Canepari . 2003. “Involvement of rpoS in the Survival of *Escherichia coli* in the Viable but Non‐Culturable State.” Environmental Microbiology 5, no. 10: 986–996. 10.1046/j.1462-2920.2003.00497.x. 14510852

[crf370439-bib-0029] Bobrovskyy, M. , and C. K. Vanderpool . 2014. “The Small RNA SgrS: Roles in Metabolism and Pathogenesis of Enteric Bacteria.” Frontiers in Cellular and Infection Microbiology 4: 61. 10.3389/fcimb.2014.00061. 24847473 PMC4021124

[crf370439-bib-0030] Bogomolnaya, L. M. , L. Aldrich , Y. Ragoza , et al. 2014. “Identification of Novel Factors Involved in Modulating Motility of *Salmonella enterica* Serotype Typhimurium.” PLoS ONE 9, no. 11: e111513. 10.1371/JOURNAL.PONE.0111513. 25369209 PMC4219756

[crf370439-bib-0031] Booth, I. R. , and C. F. Higgins . 1990. “Enteric Bacteria and Osmotic Stress: Intracellular Potassium Glutamate as a Secondary Signal of Osmotic Stress?” FEMS Microbiology Reviews 6, no. 2–3: 239–246. 10.1111/J.1574-6968.1990.TB04097.X. 1974769

[crf370439-bib-0032] Bowman, L. S. , K. M. Waterman , R. C. Williams , and M. A. Ponder . 2015. “Inoculation Preparation Affects Survival of *Salmonella enterica* on Whole Black Peppercorns and Cumin Seeds Stored at Low Water Activity.” Journal of Food Protection 78, no. 7: 1259–1265. 10.4315/0362-028X.JFP-14-483. 26197275

[crf370439-bib-0033] Brendler, T. , A. Abeles , and S. Austin . 1995. “A Protein That Binds to the P1 Origin Core and the oriC 13mer Region in a Methylation‐Specific Fashion Is the Product of the Host seqA Gene.” EMBO Journal 14, no. 16: 4083–4089. 10.1002/J.1460-2075.1995.TB00080.X. 7664748 PMC394487

[crf370439-bib-0034] Brumell, J. H. , S. Kujat‐Choy , N. F. Brown , B. A. Vallance , L. A. Knodler , and B. B. Finlay . 2003. “SopD2 is a Novel Type III Secreted Effector of *Salmonella typhimurium* That Targets Late Endocytic Compartments Upon Delivery Into Host Cells.” Traffic (Copenhagen, Denmark) 4, no. 1: 36–48. 10.1034/J.1600-0854.2003.40106.X. 12535274

[crf370439-bib-0035] Bulmer, D. M. , L. Kharraz , A. J. Grant , et al. 2012. “The Bacterial Cytoskeleton Modulates Motility, Type 3 Secretion, and Colonization in *Salmonella* .” PLOS Pathogens 8, no. 1: e1002500. 10.1371/JOURNAL.PPAT.1002500. 22291596 PMC3266929

[crf370439-bib-0036] Burgess, C. M. , A. Gianotti , N. Gruzdev , et al. 2016. “The Response of Foodborne Pathogens to Osmotic and Desiccation Stresses in the Food Chain.” International Journal of Food Microbiology 221: 37–53. 10.1016/J.IJFOODMICRO.2015.12.014. 26803272

[crf370439-bib-0037] Cameron, A. D. S. , and C. J. Dorman . 2012. “A Fundamental Regulatory Mechanism Operating Through OmpR and DNA Topology Controls Expression of *Salmonella* Pathogenicity Islands SPI‐1 and SPI‐2.” PLOS Genetics 8, no. 3: e1002615. 10.1371/JOURNAL.PGEN.1002615. 22457642 PMC3310775

[crf370439-bib-0038] CDC . 2012. 2012 Salmonella Outbreak Linked to Peanut Butter . Centers for Disease Control and Prevention. https://archive.cdc.gov/www_cdc_gov/salmonella/bredeney‐09‐12/index.html.

[crf370439-bib-0039] CDC . 2013. Salmonella Montevideo and Salmonella Mbandaka Infections Linked to Tahini Sesame Paste . Centers for Disease Control and Prevention. https://archive.cdc.gov/www_cdc_gov/salmonella/montevideo‐tahini‐05‐13/index.html.

[crf370439-bib-0040] CDC . 2014a. Salmonella Stanley Infections Linked to Raw Cashew Cheese (Final Update) . Centers for Disease Control and Prevention. https://archive.cdc.gov/www_cdc_gov/salmonella/stanley‐01‐14/index.html.

[crf370439-bib-0041] CDC . 2014b. Multistate Outbreak of Salmonella Infections Linked to Organic Sprouted Chia Powder . Centers for Disease Control and Prevention. https://archive.cdc.gov/www_cdc_gov/salmonella/newport‐05‐14/index.html.

[crf370439-bib-0042] CDC . 2014c. Salmonella Braenderup Infections Linked to Nut Butter . Centers for Disease Control and Prevention. https://archive.cdc.gov/www_cdc_gov/salmonella/braenderup‐08‐14/index.html.

[crf370439-bib-0043] CDC . 2016a. Multistate Outbreak of Salmonella Paratyphi B Variant L(+) Tartrate(+) Infections Linked to JEM Raw Brand Sprouted Nut Butter Spreads (Final Update) . Centers for Disease Control and Prevention. https://archive.cdc.gov/www_cdc_gov/salmonella/paratyphi‐b‐12‐15/index.html.

[crf370439-bib-0044] CDC . 2016b. Multistate Outbreak of Salmonella Virchow Infections Linked to Garden of Life RAW Meal Organic Shake & Meal Products . Centers for Disease Control and Prevention. https://archive.cdc.gov/www_cdc_gov/salmonella/virchow‐02‐16/index.html.

[crf370439-bib-0045] CDC . 2016c. Multistate Outbreak of Salmonella Montevideo and Salmonella Senftenberg Infections Linked to Wonderful Pistachios . Centers for Disease Control and Prevention. https://archive.cdc.gov/www_cdc_gov/salmonella/montevideo‐03‐16/index.html.

[crf370439-bib-0046] CDC . 2018a. Salmonella typhimurium Infections Linked to Dried Coconut (Final Update)—Advice to Consumers and Retailers . Centers for Disease Control and Prevention. https://archive.cdc.gov/www_cdc_gov/salmonella/typhimurium‐03‐18/advice.html.

[crf370439-bib-0047] CDC . 2018b. Multistate Outbreak of Salmonella I 4,[5],12:b:‐ Infections Linked to Kratom Products . Centers for Disease Control and Prevention. https://archive.cdc.gov/www_cdc_gov/salmonella/kratom‐02‐18/index.html.

[crf370439-bib-0048] CDC . 2018c. Salmonella Infections Linked to Hy‐Vee Spring Pasta Salad—Final Update . Centers for Disease Control and Prevention. https://archive.cdc.gov/www_cdc_gov/salmonella/sandiego‐07‐18/index.html.

[crf370439-bib-0049] CDC . 2018d. Salmonella Mbandaka Infections Linked to Kellogg's Honey Smacks Cereal (Final Update) . Centers for Disease Control and Prevention. https://archive.cdc.gov/www_cdc_gov/salmonella/mbandaka‐06‐18/index.html.

[crf370439-bib-0050] CDC . 2019a. Multistate Outbreak of Salmonella Agbeni Infections—Cake Mix . Centers for Disease Control and Prevention. https://archive.cdc.gov/www_cdc_gov/salmonella/agbeni‐11‐18/index.html.

[crf370439-bib-0051] CDC . 2019b. Salmonella Infections Linked to Tahini From Achdut Ltd . Centers for Disease Control and Prevention. https://archive.cdc.gov/www_cdc_gov/salmonella/concord‐11‐18/index.html.

[crf370439-bib-0052] CDC . 2021a. Salmonella Outbreak Linked to Jule's Cashew Brie—Investigation Details . Centers for Disease Control and Prevention. https://archive.cdc.gov/www_cdc_gov/salmonella/duisburg‐04‐21/details.html.

[crf370439-bib-0053] CDC . 2021b. Salmonella Outbreak Linked to Salami Sticks—Investigation Details . Centers for Disease Control and Prevention. https://archive.cdc.gov/www_cdc_gov/salmonella/i45‐10‐21/details.html.

[crf370439-bib-0054] CDC . 2022. Salmonella Outbreak Linked to Peanut Butter . Centers for Disease Control and Prevention. https://www.cdc.gov/salmonella/outbreaks/senftenberg‐05‐22/index.html.

[crf370439-bib-0055] CDC . 2024a. Investigation Update: Salmonella Outbreak, Dry Dog Food, November 2023 . Centers for Disease Control and Prevention. https://www.cdc.gov/salmonella/outbreaks/dog‐food‐10‐23/investigation.html.

[crf370439-bib-0056] CDC . 2024b. Investigation Update: Salmonella Outbreak, Charcuterie Meats, January 2024 . Centers for Disease Control and Prevention. https://www.cdc.gov/salmonella/outbreaks/meats‐01‐24/investigation.html.

[crf370439-bib-0057] CDC . 2024c. Investigation Update: Salmonella outbreak, Flour—March 2023 . Centers for Disease Control and Prevention. https://www.cdc.gov/salmonella/outbreaks/infantis‐03‐23/investigation.html.

[crf370439-bib-0058] CDC . 2025a. Salmonella Infection . https://www.cdc.gov/salmonella/index.html.

[crf370439-bib-0059] CDC . 2025b. Investigation Update: Pistachio Cream Outbreak, June 2025 . Centers for Disease Control and Prevention. https://www.cdc.gov/salmonella/outbreaks/pistachiocream‐06‐25/investigation.html.

[crf370439-bib-0060] Chakraborty, S. , and L. J. Kenney . 2018. “A New Role of OmpR in Acid and Osmotic Stress in *Salmonella* and *E. coli* .” Frontiers in Microbiology 9, no. NOV: 414167. 10.3389/fmicb.2018.02656. PMC6262077 30524381

[crf370439-bib-0061] Chakraborty, S. , H. Mizusaki , and L. J. Kenney . 2015. “A FRET‐Based DNA Biosensor Tracks OmpR‐Dependent Acidification of *Salmonella* During Macrophage Infection.” PLoS Biology 13, no. 4: e1002116. 10.1371/JOURNAL.PBIO.1002116. 25875623 PMC4397060

[crf370439-bib-0062] Chakroun, I. , A. Mahdhi , P. Morcillo , et al. 2018. “Motility, Biofilm Formation, Apoptotic Effect and Virulence Gene Expression of Atypical *Salmonella typhimurium* Outside and Inside Caco‐2 Cells.” Microbial Pathogenesis 114: 153–162. 10.1016/J.MICPATH.2017.11.010. 29146500

[crf370439-bib-0063] Chen, G. , M. Lin , Y. Chen , W. Xu , and H. Zhang . 2021. “Induction of a Viable but Nonculturable State, Thermal and Sanitizer Tolerance, and Gene Expression Correlation With Desiccation‐Adapted Biofilm and Planktonic *Salmonella* in Powdered Infant Formula.” Journal of Food Protection 84, no. 7: 1194–1201. 10.4315/JFP-20-402. 33770177

[crf370439-bib-0064] Chen, Z. , and X. Jiang . 2017. “Thermal Resistance and Gene Expression of Both Desiccation‐Adapted and Rehydrated *Salmonella enterica* Serovar Typhimurium Cells in Aged Broiler Litter.” Applied and Environmental Microbiology 83, no. 12: e00367‐17. 10.1128/AEM.00367-17. PMC5452820 28389541

[crf370439-bib-0065] Chiu, S. W. , S. Y. Chen , and H. C. Wong . 2008. “Localization and Expression of MreB in *Vibrio parahaemolyticus* Under Different Stresses.” Applied and Environmental Microbiology 74, no. 22: 7016–7022. 10.1128/AEM.01020-08. 18820055 PMC2583491

[crf370439-bib-0066] Choi, J. , and E. A. Groisman . 2013. “The Lipopolysaccharide Modification Regulator PmrA Limits *Salmonella* Virulence by Repressing the Type Three‐Secretion System Spi/Ssa.” Proceedings of the National Academy of Sciences of the United States of America 110, no. 23: 9499–9504. 10.1073/pnas.1303420110. 23690578 PMC3677452

[crf370439-bib-0067] Choi, S. , L. R. Beuchat , H. Kim , and J. H. Ryu . 2016. “Viability of Sprout Seeds as Affected by Treatment With Aqueous Chlorine Dioxide and Dry Heat, and Reduction of *Escherichia coli* O157:H7 and *Salmonella enterica* on Pak Choi Seeds by Sequential Treatment With Chlorine Dioxide, Drying, and Dry Heat.” Food Microbiology 54: 127–132. 10.1016/J.FM.2015.10.007.

[crf370439-bib-0068] Cirillo, D. M. , R. H. Valdivia , D. M. Monack , and S. Falkow . 1998. “Macrophage‐Dependent Induction of the *Salmonella* Pathogenicity Island 2 Type III Secretion System and Its Role in Intracellular Survival.” Molecular Microbiology 30, no. 1: 175–188. 10.1046/j.1365-2958.1998.01048.x. 9786194

[crf370439-bib-0069] Colgan, A. M. , C. Kröger , M. Diard , et al. 2016. “The Impact of 18 Ancestral and Horizontally‐Acquired Regulatory Proteins Upon the Transcriptome and sRNA Landscape of *Salmonella enterica* Serovar Typhimurium.” PLoS Genetics 12, no. 8: e1006258. 10.1371/JOURNAL.PGEN.1006258. 27564394 PMC5001712

[crf370439-bib-0070] Constantopoulos, G. , and J. A. Barranger . 1984. “Nonenzymatic Decarboxylation of Pyruvate.” Analytical Biochemistry 139, no. 2: 353–358. 10.1016/0003-2697(84)90016-2. 6476373

[crf370439-bib-0071] Crowe, J. H. , L. M. Crowe , and D. Chapman . 1984. “Preservation of Membranes in Anhydrobiotic Organisms: The Role of Trehalose.” Science 223, no. 4637: 701–703. 10.1126/science.223.4637.701. 17841031

[crf370439-bib-0072] Crucello, A. , M. M. Furtado , M. D. R. Chaves , and A. S. Sant'Ana . 2019. “Transcriptome Sequencing Reveals Genes and Adaptation Pathways in *Salmonella typhimurium* Inoculated in Four Low Water Activity Foods.” Food Microbiology 82: 426–435. 10.1016/J.FM.2019.03.016. 31027802

[crf370439-bib-0073] Csonka, L. N. 1989. “Physiological and Genetic Responses of Bacteria to Osmotic Stress.” Microbiological Reviews 53, no. 1: 121–147. 10.1128/MR.53.1.121-147.1989. 2651863 PMC372720

[crf370439-bib-0074] Csonka, L. N. , and A. D. Hanson . 1991. “Prokaryotic Osmoregulation: Genetics and Physiology.” Annual Review of Microbiology 45: 569–606. 10.1146/annurev.mi.45.100191.003033. 1741624

[crf370439-bib-0075] Cunningham, E. , C. O'Byrne , and J. D. Oliver . 2009. “Effect of Weak Acids on *Listeria monocytogenes* Survival: Evidence for a Viable but Nonculturable State in Response to Low pH.” Food Control 20, no. 12: 1141–1144. 10.1016/J.FOODCONT.2009.03.005.

[crf370439-bib-0076] Cytryn, E. J. , D. P. Sangurdekar , J. G. Streeter , et al. 2007. “Transcriptional and Physiological Responses of *Bradyrhizobium japonicum* to Desiccation‐Induced Stress.” Journal of Bacteriology 189, no. 19: 6751–6762. 10.1128/jb.00533-07. 17660288 PMC2045231

[crf370439-bib-0077] daCosta, M. S. , H. Santos , and E. A. Galinski . 1998. “An Overview of the Role and Diversity of Compatible Solutes in Bacteria and Archaea.” Advances in Biochemical Engineering/Biotechnology 61: 117–153. 10.1007/BFB0102291. 9670799

[crf370439-bib-0078] Danyluk, M. D. , T. M. Jones , S. J. Abd , F. Schlitt‐Dittrich , M. Jacobs , and L. J. Harris . 2007. “Prevalence and Amounts of *Salmonella* Found on Raw California Almonds.” Journal of Food Protection 70, no. 4: 820–827. 10.4315/0362-028X-70.4.820. 17477248

[crf370439-bib-0079] Davidson, C. J. , A. P. White , and M. G. Surette . 2008. “Evolutionary Loss of the Rdar Morphotype in *Salmonella* as a Result of High Mutation Rates During Laboratory Passage.” ISME Journal 2, no. 3: 293–307. 10.1038/ismej.2008.4. 18256702

[crf370439-bib-0080] Del Mar Lleo, M. , S. Pierobon , M. C. Tafi , C. Signoretto , and P. Canepari . 2000. “mRNA Detection by Reverse Transcription‐PCR for Monitoring Viability Over Time in an *Enterococcus faecalis* Viable but Nonculturable Population Maintained in a Laboratory Microcosm.” Applied and Environmental Microbiology 66, no. 10: 4564–4567. 10.1128/AEM.66.10.4564-4567.2000. 11010918 PMC92344

[crf370439-bib-0081] Del Mar Lleo', M. , M. C. Tafi , and P. Canepari . 1998. “Nonculturable *Enterococcus faecalis* Cells Are Metabolically Active and Capable of Resuming Active Growth.” Systematic and Applied Microbiology 21, no. 3: 333–339. 10.1016/S0723-2020(98)80041-6. 9841123

[crf370439-bib-0082] Deng, X. , Z. Li , and W. Zhang . 2012. “Transcriptome Sequencing of *Salmonella enterica* Serovar Enteritidis Under Desiccation and Starvation Stress in Peanut Oil.” Food Microbiology 30, no. 1: 311–315. 10.1016/J.FM.2011.11.001. 22265317

[crf370439-bib-0319] Denich, T. , L. Beaudette , H. Lee , and J. Trevors . 2003. “Effect of Selected Environmental and Physico‐chemical Factors on Bacterial Cytoplasmic Membranes.” Journal of Microbiological Methods 52, no. 2: 149–182. 10.1016/s0167-7012(02)00155-0. 12459238

[crf370439-bib-0083] Dillon, S. C. , A. D. S. Cameron , K. Hokamp , S. Lucchini , J. C. D. Hinton , and C. J. Dorman . 2010. “Genome‐Wide Analysis of the H‐NS and Sfh Regulatory Networks in *Salmonella typhimurium* Identifies a Plasmid‐Encoded Transcription Silencing Mechanism.” Molecular Microbiology 76, no. 5: 1250–1265. 10.1111/j.1365-2958.2010.07173.x. 20444106

[crf370439-bib-0084] Dinu, L. D. , and S. Bach . 2011. “Induction of Viable but Nonculturable *Escherichia coli* O157:H7 in the Phyllosphere of Lettuce: A Food Safety Risk Factors.” Applied and Environmental Microbiology 77, no. 23: 8295–8302. 10.1128/AEM.05020-1131535MetricsTotalCitations123TotalDownloads6.751. 21965401 PMC3233046

[crf370439-bib-0085] Doble, A. C. , D. M. Bulmer , L. Kharraz , M. H. Karavolos , and C. M. A. Khan . 2012. “The Function of the Bacterial Cytoskeleton in *Salmonella* Pathogenesis.” Virulence 3, no. 5: 446–449. 10.4161/viru.20993. 23076249 PMC3485982

[crf370439-bib-0086] dos Santos, A. M. P. , R. G. Ferrari , and C. A. Conte‐Junior . 2019. “Virulence Factors in *Salmonella typhimurium*: The Sagacity of a Bacterium.” Current Microbiology 76, no. 6: 762–773. 10.1007/s00284-018-1510-4. 29785632

[crf370439-bib-0087] Duncan, E. J. , M. E. Cheetham , J. P. Chapple , and J. Van Der Spuy . 2015. “The Role of HSP70 and Its Co‐Chaperones in Protein Misfolding, Aggregation and Disease.” Subcellular Biochemistry 78: 243–273. 10.1007/978-3-319-11731-7_12. 25487025

[crf370439-bib-0088] Dunlap, V. J. , and L. N. Csonka . 1985. “Osmotic Regulation of L‐Proline Transport in *Salmonella typhimurium* .” Journal of Bacteriology 163, no. 1: 296–304. 10.1128/JB.163.1.296-304.1985. 3924895 PMC219112

[crf370439-bib-0089] ECDC . 2017. Rapid Outbreak Assessment: Multi‐Country Outbreak of New Salmonella enterica 11:z41:e,n,z15 Infections Associated With Sesame Seeds . European Centre for Disease Prevention and Control. https://www.ecdc.europa.eu/en/publications‐data/rapid‐outbreak‐assessment‐multi‐country‐outbreak‐new‐salmonella‐enterica.

[crf370439-bib-0090] ECDC . 2018. Joint Rapid Outbreak Assessment: Multi‐Country Outbreak of Salmonella Agona Infections Linked to Infant Formula . European Centre for Disease Prevention and Control. https://www.ecdc.europa.eu/en/publications‐data/joint‐rapid‐outbreak‐assessment‐multi‐country‐outbreak‐salmonella‐agona.

[crf370439-bib-0091] ECDC . 2019a. Salmonella the most common cause of foodborne outbreaks in the European Union . European Centre for Disease Prevention and Control. https://www.ecdc.europa.eu/en/news‐events/salmonella‐most‐common‐cause‐foodborne‐outbreaks‐european‐union.

[crf370439-bib-0092] ECDC . 2019b. Rapid Outbreak Assessment: Multi‐Country Outbreak of Salmonella Poona Infections Linked to Consumption of Infant Formula . European Centre for Disease Prevention and Control. https://www.ecdc.europa.eu/en/publications‐data/rapid‐outbreak‐assessment‐multi‐country‐outbreak‐salmonella‐poona‐infections.

[crf370439-bib-0093] ECDC . 2021. Rapid Outbreak Assessment: Multi‐Country Outbreak of Multiple Salmonella enterica Serotypes Linked to Imported Sesame Based Products . European Centre for Disease Prevention and Control. https://www.ecdc.europa.eu/en/publications‐data/rapid‐outbreak‐assessment‐multi‐country‐outbreak‐multiple‐salmonella‐enterica.

[crf370439-bib-0094] ECDC . 2022. Rapid Outbreak Assessment: Multi‐Country Outbreak of Monophasic Salmonella typhimurium Sequence Type 34 Linked to Chocolate Products—First Update . European Centre for Disease Prevention and Control. https://www.ecdc.europa.eu/en/publications‐data/rapid‐outbreak‐assessment‐multi‐country‐salmonella‐outbreak‐first‐update.

[crf370439-bib-0095] Elbein, A. D. , Y. T. Pan , I. Pastuszak , and D. Carroll . 2003. “New Insights on Trehalose: A Multifunctional Molecule.” Glycobiology 13, no. 4: 17R–27R. 10.1093/GLYCOB/CWG047. 12626396

[crf370439-bib-0096] Ellermeier, C. D. , J. R. Ellermeier , and J. M. Slauch . 2005. “HilD, HilC and RtsA Constitute a Feed Forward Loop That Controls Expression of the SPI1 Type Three Secretion System Regulator hilA in *Salmonella enterica* Serovar Typhimurium.” Molecular Microbiology 57, no. 3: 691–705. 10.1111/J.1365-2958.2005.04737.X. 16045614

[crf370439-bib-0097] Empadinhas, N. , and M. S. Da Costa . 2008. “Osmoadaptation Mechanisms in Prokaryotes: Distribution of Compatible Solutes.” International Microbiology 11, no. 3: 151–161. 10.2436/20.1501.01.55. 18843593

[crf370439-bib-0098] Eprintsev, A. T. , A. V. Salnikov , A. M. Haba , and M. V. Zaichikova . 2014. “Isocitrate Lyase Isozymes and Their Role in Organisms With Different Levels of Organization.” Biology Bulletin Reviews 4, no. 4: 323–334. 10.1134/S2079086414040021.

[crf370439-bib-0099] Epstein, W. 1986. “Osmoregulation by Potassium Transport in *Escherichia coli* .” FEMS Microbiology Reviews 2, no. 1–2: 73–78. 10.1111/J.1574-6968.1986.TB01845.X.

[crf370439-bib-0100] Epstein, W. 2003. “The Roles and Regulation of Potassium in Bacteria.” Progress in Nucleic Acid Research and Molecular Biology 75: 293–320. 10.1016/S0079-6603(03)75008-9. 14604015

[crf370439-bib-0101] Eymann, C. , G. Homuth , C. Scharf , and M. Hecker . 2002. “ *Bacillus subtilis* Functional Genomics: Global Characterization of the Stringent Response by Proteome and Transcriptome Analysis.” Journal of Bacteriology 184, no. 9: 2500–2520. 10.1128/jb.184.9.2500-2520.2002. 11948165 PMC134987

[crf370439-bib-0102] Fàbrega, A. , and J. Vila . 2013. “ *Salmonella enterica* Serovar Typhimurium Skills to Succeed in the Host: Virulence and Regulation.” Clinical Microbiology Reviews 26, no. 2: 308–341. 10.1128/cmr.00066-12. 23554419 PMC3623383

[crf370439-bib-0103] Fang, F. C. , S. J. Libby , M. E. Castor , and A. M. Fung . 2005. “Isocitrate Lyase (AceA) Is Required for *Salmonella* Persistence but Not for Acute Lethal Infection in Mice.” Infection and Immunity 73, no. 4: 2547–2549. 10.1128/iai.73.4.2547-2549.2005. 15784602 PMC1087437

[crf370439-bib-0104] Fang, Y. , L. M. McMullen , and M. G. Gänzle . 2020. “Effect of Drying on Oxidation of Membrane Lipids and Expression of Genes Encoded by the Shiga Toxin Prophage in *Escherichia coli* .” Food Microbiology 86: 103332. 10.1016/J.FM.2019.103332. 31703888

[crf370439-bib-0105] Fardini, Y. , K. Chettab , O. Grépinet , et al. 2007. “The YfgL Lipoprotein Is Essential for Type III Secretion System Expression and Virulence of *Salmonella enterica* Serovar Enteritidis.” Infection and Immunity 75, no. 1: 358–370. 10.1128/iai.00716-06. 17060472 PMC1828421

[crf370439-bib-0106] Fardini, Y. , J. Trotereau , E. Bottreau , C. Souchard , P. Velge , and I. Virlogeux‐Payant . 2009. “Investigation of the Role of the BAM Complex and SurA Chaperone in Outer‐Membrane Protein Biogenesis and Type III Secretion System Expression in *Salmonella* .” Microbiology (Reading, England) 155, no. 5: 1613–1622. 10.1099/mic.0.025155-0. 19372159

[crf370439-bib-0107] Feeney, A. , C. D. Johnston , R. Govender , J. O'Mahony , A. Coffey , and R. D. Sleator . 2014. “Analysis of the Role of the *Cronobacter sakazakii* ProP Homologues in Osmotolerance.” Gut Pathogens 6, no. 1: 1–9. 10.1186/1757-4749-6-15. 24910715 PMC4047261

[crf370439-bib-0108] Feng, X. , R. Oropeza , and L. J. Kenney . 2003. “Dual Regulation by Phospho‐OmpR of ssrA/B Gene Expression in *Salmonella* Pathogenicity Island 2.” Molecular Microbiology 48, no. 4: 1131–1143. 10.1046/j.1365-2958.2003.03502.x. 12753201

[crf370439-bib-0109] Feng, X. , R. Oropeza , D. Walthers , and L. J. Kenney . 2003. “OmpR Phosphorylation and Its Role in Signaling and Pathogenesis.” ASM News‐American Society for Microbiology 69, no. 8: 390–395.

[crf370439-bib-0110] Feng, X. , D. Walthers , R. Oropeza , and L. J. Kenney . 2004. “The Response Regulator SsrB Activates Transcription and Binds to a Region Overlapping OmpR Binding Sites at *Salmonella* Pathogenicity Island 2.” Molecular Microbiology 54, no. 3: 823–835. 10.1111/j.1365-2958.2004.04317.x. 15491370

[crf370439-bib-0111] Fernández‐Salguero, J. , R. Gómez , and M. A. Carmona . 1993. “Water Activity in Selected High‐Moisture Foods.” Journal of Food Composition and Analysis 6, no. 4: 364–369. 10.1006/JFCA.1993.1040.

[crf370439-bib-0112] Fink, R. C. , M. R. Evans , S. Porwollik , et al. 2007. “FNR Is a Global Regulator of Virulence and Anaerobic Metabolism in *Salmonella enterica* Serovar Typhimurium (ATCC 14028s).” Journal of Bacteriology 189, no. 6: 2262–2273. 10.1128/jb.00726-06. 17220229 PMC1899381

[crf370439-bib-0113] Finka, A. , S. K. Sharma , and P. Goloubinoff . 2015. “Multi‐Layered Molecular Mechanisms of Polypeptide Holding, Unfolding and Disaggregation by HSP70/HSP110 Chaperones.” Frontiers in Molecular Biosciences 2: 29. 10.3389/fmolb.2015.00029. 26097841 PMC4456865

[crf370439-bib-0114] Finn, S. , O. Condell , P. McClure , A. Amézquita , and S. Fanning . 2013. “Mechanisms of Survival, Responses, and Sources of *Salmonella* in Low‐Moisture Environments.” Frontiers in Microbiology 4: 331. 10.3389/fmicb.2013.00331. 24294212 PMC3827549

[crf370439-bib-0115] Finn, S. , K. Hïndler , O. Condell , et al. 2013. “ProP Is Required for the Survival of Desiccated Salmonella Enterica Serovar Typhimurium Cells on a Stainless Steel Surface.” Applied and Environmental Microbiology 79, no. 14: 4376–4384. 10.1128/aem.00515-13. 23666329 PMC3697505

[crf370439-bib-0116] Finn, S. , J. C. D. Hinton , P. McClure , A. Amézquita , M. Martins , and S. Fanning . 2013. “Phenotypic Characterization of *Salmonella* Isolated From Food Production Environments Associated With Low–Water Activity Foods.” Journal of Food Protection 76, no. 9: 1488–1499. 10.4315/0362-028X.JFP-13-088. 23992493

[crf370439-bib-0117] Fischer, E. , and U. Sauer . 2003. “Metabolic Flux Profiling of *Escherichia coli* Mutants in Central Carbon Metabolism Using GC‐MS.” European Journal of Biochemistry 270, no. 5: 880–891. 10.1046/J.1432-1033.2003.03448.X. 12603321

[crf370439-bib-0118] Fong, K. , and S. Wang . 2016. “Heat Resistance of *Salmonella enterica* Is Increased by Pre‐Adaptation to Peanut Oil or Sub‐Lethal Heat Exposure.” Food Microbiology 58: 139–147. 10.1016/J.FM.2016.04.004. 27217370

[crf370439-bib-0119] França, M. B. , A. D. Panek , and E. C. A. Eleutherio . 2007. “Oxidative Stress and Its Effects During Dehydration.” Comparative Biochemistry and Physiology Part A: Molecular & Integrative Physiology 146, no. 4: 621–631. 10.1016/J.CBPA.2006.02.030. 16580854

[crf370439-bib-0120] Frossard, S. M. , A. A. Khan , E. C. Warrick , et al. 2012. “Identification of a Third Osmoprotectant Transport System, the osmU System, in *Salmonella enterica* .” Journal of Bacteriology 194, no. 15: 3861–3871. 10.1128/jb.00495-12. 22609924 PMC3416524

[crf370439-bib-0121] Gao, M. , F. Tan , Y. Shen , and Y. Peng . 2024. “Rapid Detection Method of Bacterial Pathogens in Surface Waters and a New Risk Indicator for Water Pathogenic Pollution.” Scientific Reports 14, no. 1: 1614. 10.1038/s41598-023-49774-y. 38238351 PMC10796392

[crf370439-bib-0122] García, A. H. 2011. “Anhydrobiosis in Bacteria: From Physiology to Applications.” Journal of Biosciences 36, no. 5: 939–950. 10.1007/s12038-011-9107-0. 22116292

[crf370439-bib-0123] Garmiri, P. , K. E. Coles , T. J. Humphrey , and T. A. Cogan . 2008. “Role of Outer Membrane Lipopolysaccharides in the Protection of *Salmonella enterica* Serovar Typhimurium From Desiccation Damage.” FEMS Microbiology Letters 281, no. 2: 155–159. 10.1111/J.1574-6968.2008.01093.X. 18312578

[crf370439-bib-0124] Gast, R. K. , and R. E. Porter . 2020. “ *Salmonella* Infections.” In Diseases of Poultry, edited by D. E. Swayne , M. Boulianne , C. M. Logue , et al., 717–753. Wiley. 10.1002/9781119371199.ch16.

[crf370439-bib-0125] Gerlach, R. G. , D. Jäckel , N. Geymeier , and M. Hensel . 2007. “ *Salmonella* Pathogenicity Island 4‐Mediated Adhesion Is Coregulated With Invasion Genes in *Salmonella enterica* .” Infection and Immunity 75, no. 10: 4697–4709. 10.1128/iai.00228-07. 17635868 PMC2044552

[crf370439-bib-0126] Gharzouli, R. , M. A. Carpéné , F. Couderc , A. Benguedouar , and V. Poinsot . 2013. “Relevance of Fucose‐Rich Extracellular Polysaccharides Produced by *Rhizobium sullae* Strains Nodulating *Hedysarum coronarium* L. Legumes.” Applied and Environmental Microbiology 79, no. 6: 1764–1776. 10.1128/AEM.02903-12. 23183977 PMC3592251

[crf370439-bib-0127] Ghezzi, J. I. , and T. R. Steck . 1999. “Induction of the Viable but Non‐Culturable Condition in *Xanthomonas campestris* pv. Campestris in Liquid Microcosms and Sterile Soil.” FEMS Microbiology Ecology 30, no. 3: 203–208. 10.1111/J.1574-6941.1999.TB00648.X. 10525176

[crf370439-bib-0128] Gibson, D. L. , A. P. White , S. D. Snyder , et al. 2006. “ *Salmonella* Produces an O‐Antigen Capsule Regulated by AgfD and Important for Environmental Persistence.” Journal of Bacteriology 188, no. 22: 7722–7730. 10.1128/jb.00809-06. 17079680 PMC1636306

[crf370439-bib-0129] Gill, O. N. , C. L. R. Bartlett , P. N. Sockett , et al. 1983. “Outbreak of *Salmonella* Napoli Infection Caused by Contaminated Chocolate Bars.” Lancet 321, no. 8324: 574–577. 10.1016/S0140-6736(83)92822-2. 6131266

[crf370439-bib-0130] Göing, S. , and K. Jung . 2021. “Viable but Nonculturable Gastrointestinal Bacteria and Their Resuscitation.” Archives of Gastroenterology Research 2, no. 2: 55–62. 10.33696/GASTROENTEROLOGY.2.027.

[crf370439-bib-0131] Gong, Y. , X. Li , J. Wang , Y. Zhao , J. Meng , and L. Zhai . 2025. “Unveiling *Salmonella* Derby Survival: Stress Responses to Prolonged Hyperosmotic Stress.” Foods 14, no. 9: 1440. 10.3390/foods14091440. 40361524 PMC12072157

[crf370439-bib-0132] Gourmelon, M. , J. Cillard , and M. Pommepuy . 1994. “Visible Light Damage to *Escherichia coli* in Seawater: Oxidative Stress Hypothesis.” Journal of Applied Bacteriology 77, no. 1: 105–112. 10.1111/j.1365-2672.1994.tb03051.x. 7928776

[crf370439-bib-0133] Grogan, D. W. , J. E. Cronan , W. Grogan , and J. E. Cronan . 1986. “Characterization of *Escherichia coli* Mutants Completely Defective in Synthesis of Cyclopropane Fatty Acids.” Journal of Bacteriology 166, no. 3: 872–877. 10.1128/JB.166.3.872-877.1986. 3519583 PMC215207

[crf370439-bib-0134] Gruzdev, N. , M. McClelland , S. Porwollik , S. Ofaim , R. Pinto , and S. Saldinger‐Sela . 2012. “Global Transcriptional Analysis of Dehydrated *Salmonella enterica* Serovar Typhimurium.” Applied and Environmental Microbiology 78, no. 22: 7866–7875. 10.1128/AEM.01822-12. 22941081 PMC3485933

[crf370439-bib-0135] Gruzdev, N. , R. Pinto , and S. Sela . 2011. “Effect of Desiccation on Tolerance of *Salmonella Enterica* to Multiple Stresses.” Applied and Environmental Microbiology 77, no. 5: 1667–1673. 10.1128/AEM.02156-10. 21216905 PMC3067256

[crf370439-bib-0136] Gruzdev, N. , R. Pinto , and S. Sela Saldinger . 2012. “Persistence of *Salmonella enterica* During Dehydration and Subsequent Cold Storage.” Food Microbiology 32, no. 2: 415–422. 10.1016/j.fm.2012.08.003. 22986208

[crf370439-bib-0137] Guiney, D. G. , S. Libby , F. C. Fang , M. Krause , and J. Fierer . 1995. “Growth‐Phase Regulation of Plasmid Virulence Genes in *Salmonella* .” Trends in Microbiology 3, no. 7: 275–279. 10.1016/S0966-842X(00)88944-1. 7551642

[crf370439-bib-0138] Guo, M. S. , and C. A. Gross . 2014. “Stress‐Induced Remodeling of the Bacterial Proteome.” Current Biology 24, no. 10: R424–R434. 10.1016/j.cub.2014.03.023. 24845675 PMC4089988

[crf370439-bib-0139] Gupte, A. R. , C. L. E. De Rezende , and S. W. Joseph . 2003. “Induction and Resuscitation of Viable but Nonculturable *Salmonella enterica* Serovar Typhimurium DT104.” Applied and Environmental Microbiology 69, no. 11: 6669–6675. 10.1128/AEM.69.11.6669-6675.2003. 14602627 PMC262293

[crf370439-bib-0140] Hall, M. N. , and T. J. Silhavy . 1981a. “Genetic Analysis of the Major Outer Membrane Proteins of *Escherichia coli* .” Annual Review of Genetics 15: 91–142. 10.1146/annurev.ge.15.120181.000515. 7039497

[crf370439-bib-0141] Hall, M. N. , and T. J. Silhavy . 1981b. “The ompB Locus and the Regulation of the Major Outer Membrane Porin Proteins of *Escherichia coli* K12.” Journal of Molecular Biology 146, no. 1: 23–43. 10.1016/0022-2836(81)90364-8. 7021856

[crf370439-bib-0142] Handford, J. I. , B. Ize , G. Buchanan , et al. 2009. “Conserved Network of Proteins Essential for Bacterial Viability.” Journal of Bacteriology 191, no. 15: 4732–4749. 10.1128/jb.00136-09. 19376873 PMC2715707

[crf370439-bib-0143] Hansen, A. M. , Y. Qiu , N. Yeh , F. R. Blattner , T. Durfee , and D. J. Jin . 2005. “SspA Is Required for Acid Resistance in Stationary Phase by Downregulation of H‐NS in *Escherichia coli* .” Molecular Microbiology 56, no. 3: 719–734. 10.1111/j.1365-2958.2005.04567.x. 15819627

[crf370439-bib-0144] Hariharan, V. , A. R. Chowdhury , S. Rao S , D. Chakravortty , and S. Basu . 2023. “phoP Maintains the Environmental Persistence and Virulence of Pathogenic Bacteria in Mechanically Stressed Desiccated Droplets.” Iscience 26, no. 5: 106580. 10.1016/j.isci.2023.106580. 37168573 PMC10164896

[crf370439-bib-0145] Hassett, D. J. , J. F. Ma , J. G. Elkins , et al. 1999. “Quorum Sensing in *Pseudomonas aeruginosa* Controls Expression of Catalase and Superoxide Dismutase Genes and Mediates Biofilm Susceptibility to Hydrogen Peroxide.” Molecular Microbiology 34, no. 5: 1082–1093. 10.1046/j.1365-2958.1999.01672.x. 10594832

[crf370439-bib-0146] He, Y. , D. Guo , J. Yang , M. L. Tortorello , and W. Zhang . 2011. “Survival and Heat Resistance of *Salmonella enterica* and *Escherichia coli* O157:H7 in Peanut Butter.” Applied and Environmental Microbiology 77, no. 23: 8434–8438. 10.1128/AEM.06270-11. 21965404 PMC3233057

[crf370439-bib-0147] Hecker, M. , C. Heim , U. Völker , and L. Wölfel . 1988. “Induction of Stress Proteins by Sodium Chloride Treatment in *Bacillus subtilis* .” Archives of Microbiology 150, no. 6: 564–566. 10.1007/BF00408250. 3144958

[crf370439-bib-0148] Hoefman, S. , K. van Hoorde , N. Boon , P. Vandamme , P. de Vos , and K. Heylen . 2012. “Survival or Revival: Long‐Term Preservation Induces a Reversible Viable but Non‐Culturable State in Methane‐Oxidizing Bacteria.” PLoS ONE 7, no. 4: e34196. 10.1371/JOURNAL.PONE.0034196. 22539945 PMC3335116

[crf370439-bib-0149] Horlacher, R. , K. Uhland , W. Klein , M. Ehrmann , and W. Boos . 1996. “Characterization of a Cytoplasmic Trehalase of *Escherichia coli* .” Journal of Bacteriology 178, no. 21: 6250–6257. 10.1128/JB.178.21.6250-6257.1996. 8892826 PMC178497

[crf370439-bib-0150] Hu, J. , L. J. Worrall , M. Vuckovic , et al. 2019. “T3S Injectisome Needle Complex Structures in Four Distinct States Reveal the Basis of Membrane Coupling and Assembly.” Nature Microbiology 4, no. 11: 2010–2019. 10.1038/s41564-019-0545-z. 31427728

[crf370439-bib-0151] Huang, K. C. , Y. Meir , and N. S. Wingreen . 2003. “Dynamic Structures in *Escherichia coli*: Spontaneous Formation of MinE Rings and MinD Polar Zones.” Proceedings of the National Academy of Sciences of the United States of America 100, no. 22: 12724–12728. 10.1073/pnas.2135445100. 14569005 PMC240685

[crf370439-bib-0152] Huang, X. , H. Xu , X. Sun , K. Ohkusu , Y. Kawamura , and T. Ezaki . 2007. “Genome‐Wide Scan of the Gene Expression Kinetics of *Salmonella enterica* Serovar Typhi During Hyperosmotic Stress.” International Journal of Molecular Sciences 8, no. 2: 116–135. 10.3390/I8020116.

[crf370439-bib-0153] Humann, J. L. , and M. L. Kahn . 2015. “Genes Involved in Desiccation Resistance of Rhizobia and Other Bacteria.” Biological Nitrogen Fixation 1–2: 397–404. 10.1002/9781119053095.ch39.

[crf370439-bib-0154] Humphrey, T. 2004. “ *Salmonella*, Stress Responses and Food Safety.” Nature Reviews Microbiology 2, no. 6: 504–509. 10.1038/nrmicro907. 15152206

[crf370439-bib-0155] Jay, J. M. , M. J. Loessner , and D. A. Golden . 2005. Modern Food Microbiology. 7th ed.. Springer. 10.1007/b100840.

[crf370439-bib-0156] Jay, J. M. , M. J. Loessner , and D. A. Golden . 2008. Modern Food Microbiology, Food Science Text Series. 7th ed.. Springer Science & Business Media.

[crf370439-bib-0157] Jayeola, V. , J. M. Farber , and S. Kathariou . 2022. “Induction of the Viable‐But‐Nonculturable State in *Salmonella* Contaminating Dried Fruit.” Applied and Environmental Microbiology 88, no. 2: e0173321. 10.1128/AEM.01733-21. 34731057 PMC8788685

[crf370439-bib-0158] Jayeola, V. , M. McClelland , S. Porwollik , W. Chu , J. Farber , and S. Kathariou . 2020. “Identification of Novel Genes Mediating Survival of *Salmonella* on Low‐Moisture Foods via Transposon Sequencing Analysis.” Frontiers in Microbiology 11: 726. 10.3389/fmicb.2020.00726. 32499760 PMC7242855

[crf370439-bib-0159] Jenniches, L. , C. Michaux , L. Popella , et al. 2024. “Improved RNA Stability Estimation Through Bayesian Modeling Reveals Most *Salmonella* Transcripts Have Subminute Half‐Lives.” Proceedings of the National Academy of Sciences of the United States of America 121, no. 14: e2308814121. 10.1073/pnas.2308814121. 38527194 PMC10998600

[crf370439-bib-0160] Jiang, X. , and T. J. Chai . 1996. “Survival of *Vibrio parahaemolyticus* at Low Temperatures Under Starvation Conditions and Subsequent Resuscitation of Viable, Nonculturable Cells.” Applied and Environmental Microbiology 62, no. 4: 1300–1305. 10.1128/AEM.62.4.1300-1305.1996. 8919790 PMC167895

[crf370439-bib-0161] Jiang, X. , O. W. Rossanese , N. F. Brown , et al. 2004. “The Related Effector Proteins SopD and SopD2 From *Salmonella enterica* Serovar Typhimurium Contribute to Virulence During Systemic Infection of Mice.” Molecular Microbiology 54, no. 5: 1186–1198. 10.1111/j.1365-2958.2004.04344.x. 15554961

[crf370439-bib-0162] Jones, M. A. , M. W. Wood , P. B. Mullan , P. R. Watson , T. S. Wallis , and E. E. Galyov . 1998. “Secreted Effector Proteins of *Salmonella* Dublin Act in Concert to Induce Enteritis.” Infection and Immunity 66, no. 12: 5799–5804. 10.1128/iai.66.12.5799-5804.1998. 9826357 PMC108733

[crf370439-bib-0163] Kang, Y. , K. D. Weber , Y. Qiu , P. J. Kiley , and F. R. Blattner . 2005. “Genome‐Wide Expression Analysis Indicates That FNR of *Escherichia coli* K‐12 Regulates a Large Number of Genes of Unknown Function.” Journal of Bacteriology 187, no. 3: 1135–1160. 10.1128/jb.187.3.1135-1160.2005. 15659690 PMC545700

[crf370439-bib-0164] KEGG . n.d. “Kyoto Encyclopedia of Genes and Genomes—KEGG PATHWAY Database.” Accessed June 21, 2025. https://www.genome.jp/kegg/pathway.html.

[crf370439-bib-0165] Kelly, A. , M. D. Goldberg , R. K. Carroll , V. Danino , J. C. D. Hinton , and C. J. Dorman . 2004. “A Global Role for Fis in the Transcriptional Control of Metabolism and Type III Secretion in *Salmonella enterica* Serovar Typhimurium.” Microbiology (Reading, England) 150, no. 7: 2037–2053. 10.1099/mic.0.27209-0. 15256548

[crf370439-bib-0166] Kempf, B. , and E. Bremer . 1998. “Uptake and Synthesis of Compatible Solutes as Microbial Stress Responses to High‐Osmolality Environments.” Archives of Microbiology 170, no. 5: 319–330. 10.1007/s002030050649. 9818351

[crf370439-bib-0167] Kieboom, J. , H. D. Kusumaningrum , M. H. Tempelaars , W. C. Hazeleger , T. Abee , and R. R. Beumer . 2006. “Survival, Elongation, and Elevated Tolerance of *Salmonella enterica* Serovar Enteritidis at Reduced Water Activity.” Journal of Food Protection 69, no. 11: 2681–2686. 10.4315/0362-028X-69.11.2681. 17133811

[crf370439-bib-0168] Kisker, C. , J. Kuper , and B. Van Houten . 2013. “Prokaryotic Nucleotide Excision Repair.” Cold Spring Harbor Perspectives in Biology 5, no. 3: a012591. 10.1101/CSHPERSPECT.A012591. 23457260 PMC3578354

[crf370439-bib-0169] Klein, W. , R. Horlacher , and W. Boos . 1995. “Molecular Analysis of treB Encoding the *Escherichia coli* Enzyme II Specific for Trehalose.” Journal of Bacteriology 177, no. 14: 4043–4052. 10.1128/jb.177.14.4043-4052.1995. 7608078 PMC177135

[crf370439-bib-0170] Kocharunchitt, C. , T. King , K. Gobius , J. P. Bowman , and T. Ross . 2014. “Global Genome Response of *Escherichia coli* O157∶H7 Sakai During Dynamic Changes in Growth Kinetics Induced by an Abrupt Downshift in Water Activity.” PLoS ONE 9, no. 3: e90422. 10.1371/JOURNAL.PONE.0090422. 24594867 PMC3940904

[crf370439-bib-0171] Kornberg, H. L. , and H. A. Krebs . 1957. “Synthesis of Cell Constituents From C2‐Units by a Modified Tricarboxylic Acid Cycle.” Nature 179, no. 4568: 988–991. 10.1038/179988a0. 13430766

[crf370439-bib-0172] Kröger, C. , A. Colgan , S. Srikumar , et al. 2013. “An Infection‐Relevant Transcriptomic Compendium for *Salmonella enterica* Serovar Typhimurium.” Cell Host & Microbe 14, no. 6: 683–695. 10.1016/J.CHOM.2013.11.010. 24331466

[crf370439-bib-0173] Kröger, C. , S. C. Dillon , A. D. S. Cameron , et al. 2012. “The Transcriptional Landscape and Small RNAs of *Salmonella enterica* Serovar Typhimurium.” Proceedings of the National Academy of Sciences of the United States of America 109, no. 20: E1277–E1286. 10.1073/pnas.1201061109. 22538806 PMC3356629

[crf370439-bib-0174] Kubori, T. , Y. Matsushima , D. Nakamura , et al. 1998. “Supramolecular Structure of the *Salmonella typhimurium* Type III Protein Secretion System.” Science 280, no. 5363: 602–605. 10.1126/science.280.5363.602. 9554854

[crf370439-bib-0175] Kusumoto, A. , H. Asakura , and K. Kawamoto . 2012. “General Stress Sigma Factor RpoS Influences Time Required to Enter the Viable but Non‐Culturable State in *Salmonella enterica* .” Microbiology and Immunology 56, no. 4: 228–237. 10.1111/J.1348-0421.2012.00428.X. 22256797

[crf370439-bib-0176] Kuzminov, A. 2011. “Homologous Recombination—Experimental Systems, Analysis, and Significance.” EcoSal Plus 4, no. 2: 1–44. 10.1128/ecosalplus.7.2.6. PMC4190071 26442506

[crf370439-bib-0177] Lamichhane‐Khadka, R. , J. G. Frye , S. Porwollik , M. McClelland , and R. J. Maier . 2011. “Hydrogen‐Stimulated Carbon Acquisition and Conservation in *Salmonella enterica* Serovar Typhimurium.” Journal of Bacteriology 193, no. 20: 5824–5832. 10.1128/jb.05456-11. 21856852 PMC3187206

[crf370439-bib-0178] Lang, E. , S. Guyot , P. Alvarez‐Martin , J. M. Perrier‐Cornet , and P. Gervais . 2017. “Caco‐2 Invasion by *Cronobacter sakazakii* and *Salmonella enterica* Exposed to Drying and Heat Treatments in Dried state in Milk Powder.” Frontiers in Microbiology 8: 1893. 10.3389/fmicb.2017.01893. 29033925 PMC5627024

[crf370439-bib-0179] Lange, R. , M. Barth , and R. Hengge‐Aronis . 1993. “Complex Transcriptional Control of the Sigma s‐Dependent Stationary‐Phase‐Induced and Osmotically Regulated osmY (csi‐5) Gene Suggests Novel Roles for Lrp, Cyclic AMP (cAMP) Receptor Protein‐cAMP Complex, and Integration Host Factor in the Stationary‐Phase Response of *Escherichia coli* .” Journal of Bacteriology 175, no. 24: 7910–7917. 10.1128/JB.175.24.7910-7917.1993. 8253679 PMC206969

[crf370439-bib-0180] Larsen, P. I. , L. K. Sydnes , B. Landfald , and A. R. Strøm . 1987. “Osmoregulation in *Escherichia coli* by Accumulation of Organic Osmolytes: Betaines, Glutamic Acid, and Trehalose.” Archives of Microbiology 147, no. 1: 1–7. 10.1007/BF00492896. 2883950

[crf370439-bib-0181] Lebre, P. H. , P. De Maayer , and D. A. Cowan . 2017. “Xerotolerant Bacteria: Surviving Through a Dry Spell.” Nature Reviews Microbiology 15, no. 5: 285–296. 10.1038/nrmicro.2017.16. 28316329

[crf370439-bib-0182] Lee, A. K. , C. S. Detweiler , and S. Falkow . 2000. “OmpR Regulates the Two‐Component System SsrA‐SsrB in *Salmonella* Pathogenicity Island 2.” Journal of Bacteriology 182, no. 3: 771–781. 10.1128/jb.182.3.771-781.2000. 10633113 PMC94342

[crf370439-bib-0183] Lee, S. J. , and J. D. Gralla . 2004. “Osmo‐Regulation of Bacterial Transcription via Poised RNA Polymerase.” Molecular Cell 14, no. 2: 153–162. 10.1016/S1097-2765(04)00202-3. 15099515

[crf370439-bib-0184] Li, H. , A. Bhaskara , C. Megalis , and M. L. Tortorello . 2012. “Transcriptomic Analysis of *Salmonella* Desiccation Resistance.” Foodborne Pathogens and Disease 9, no. 12: 1143–1151. 10.1089/fpd.2012.1254. 23237410

[crf370439-bib-0185] Li, J. , E. S. Nakayasu , C. C. Overall , et al. 2015. “Global Analysis of *Salmonella* Alternative Sigma Factor E on Protein Translation.” Journal of Proteome Research 14, no. 4: 1716–1726. 10.1021/pr5010423. 25686268 PMC4476319

[crf370439-bib-0186] Li, L. , N. Mendis , H. Trigui , J. D. Oliver , and S. P. Faucher . 2014. “The Importance of the Viable but Non‐Culturable State in Human Bacterial Pathogens.” Frontiers in Microbiology 5: 258. 10.3389/fmicb.2014.00258. 24917854 PMC4040921

[crf370439-bib-0187] Li, Y. , T. Huang , C. Bai , et al. 2020. “Reduction, Prevention, and Control of *Salmonella enterica* Viable but Non‐Culturable Cells in Flour Food.” Frontiers in Microbiology 11: 1859. 10.3389/fmicb.2020.01859. 32973696 PMC7472744

[crf370439-bib-0188] Li, Y. , L. Yang , J. Fu , M. Yan , D. Chen , and L. Zhang . 2017. “The Novel Loop‐Mediated Isothermal Amplification Based Confirmation Methodology on the Bacteria in Viable but Non‐Culturable (VBNC) State.” Microbial Pathogenesis 111: 280–284. 10.1016/J.MICPATH.2017.09.007. 28888887

[crf370439-bib-0189] Liao, H. , X. Zhong , L. Xu , et al. 2019. “Quorum‐Sensing Systems Trigger Catalase Expression to Reverse the oxyR Deletion‐Mediated VBNC State in *Salmonella typhimurium* .” Research in Microbiology 170, no. 2: 65–73. 10.1016/J.RESMIC.2018.10.004. 30414454

[crf370439-bib-0190] Limcharoenchat, P. , M. K. James , and B. P. Marks . 2019. “Survival and Thermal Resistance of *Salmonella* Enteritidis PT 30 on Almonds After Long‐Term Storage.” Journal of Food Protection 82, no. 2: 194–199. 10.4315/0362-028X.JFP-18-152. 30667289

[crf370439-bib-0191] Lindbäck, T. , M. E. Rottenberg , S. M. Roche , and L. M. Rørvik . 2010. “The Ability to Enter Into an Avirulent Viable but Non‐Culturable (VBNC) Form Is Widespread Among *Listeria monocytogenes* Isolates From Salmon, Patients and Environment.” Veterinary Research 41, no. 1: 1–10. 10.1051/VETRES/2009056. 19796607 PMC2775167

[crf370439-bib-0192] Liu, J. , R. Zhou , L. Li , et al. 2017. “Viable but Non‐Culturable State and Toxin Gene Expression of Enterohemorrhagic *Escherichia coli* O157 Under Cryopreservation.” Research in Microbiology 168, no. 3: 188–193. 10.1016/J.RESMIC.2016.11.002. 27884785

[crf370439-bib-0193] Liu, S. , J. Tang , R. K. Tadapaneni , R. Yang , and M. J. Zhu . 2018. “Exponentially Increased Thermal Resistance of *Salmonella* spp. and *Enterococcus faecium* at Reduced Water Activity.” Applied and Environmental Microbiology 84, no. 8: e02742‐17. 10.1128/AEM.02742-17. 29439987 PMC5881056

[crf370439-bib-0194] Lleò, M. M. , B. Bonato , M. C. Tafi , C. Signoretto , M. Boaretti , and P. Canepari . 2001. “Resuscitation Rate in Different Enterococcal Species in the Viable but Non‐Culturable State.” Journal of Applied Microbiology 91, no. 6: 1095–1102. 10.1046/J.1365-2672.2001.01476.X. 11851818

[crf370439-bib-0195] Lucas, R. L. , and C. A. Lee . 2000. “Unravelling the Mysteries of Virulence Gene Regulation in *Salmonella typhimurium* .” Molecular Microbiology 36, no. 5: 1024–1033. 10.1046/j.1365-2958.2000.01961.x. 10844688

[crf370439-bib-0196] Lucchini, S. , G. Rowley , M. D. Goldberg , D. Hurd , M. Harrison , and J. C. D. Hinton . 2006. “H‐NS Mediates the Silencing of Laterally Acquired Genes in Bacteria.” PLOS Pathogens 2, no. 8: e81. 10.1371/JOURNAL.PPAT.0020081. 16933988 PMC1550270

[crf370439-bib-0197] Ma, Y. , Y. Deng , Z. Xu , et al. 2017. “Development of a Propidium Monoazide‐Polymerase Chain Reaction Assay for Detection of Viable *Lactobacillus brevis* in Beer.” Brazilian Journal of Microbiology 48, no. 4: 740–746. 10.1016/J.BJM.2016.11.012. 28633981 PMC5628306

[crf370439-bib-0198] MacMillan, S. V. , D. A. Alexander , D. E. Culham , et al. 1999. “The Ion Coupling and Organic Substrate Specificities of Osmoregulatory Transporter ProP in *Escherichia coli* .” Biochimica et Biophysica Acta (BBA)—Biomembranes 1420, no. 1–2: 30–44. 10.1016/S0005-2736(99)00085-1. 10446288

[crf370439-bib-0199] Makino, S. I. , T. Kii , H. Asakura , et al. 2000. “Does Enterohemorrhagic *Escherichia coli* O157:H7 Enter the Viable but Nonculturable State in Salted Salmon Roe?” Applied and Environmental Microbiology 66, no. 12: 5536–5539. 10.1128/AEM.66.12.5536-5539.2000. 11097946 PMC92500

[crf370439-bib-0200] Mandal, R. K. , and Y. M. Kwon . 2017. “Global Screening of *Salmonella enterica* Serovar Typhimurium Genes for Desiccation Survival.” Frontiers in Microbiology 8, no. SEP: 278455. 10.3389/fmicb.2017.01723. PMC5596212 28943871

[crf370439-bib-0201] Marcus, S. L. , J. H. Brumell , C. G. Pfeifer , and B. B. Finlay . 2000. “ *Salmonella* Pathogenicity Islands: Big Virulence in Small Packages.” Microbes and Infection 2, no. 2: 145–156. 10.1016/S1286-4579(00)00273-2. 10742687

[crf370439-bib-0202] Marinus, M. G. , and A. Løbner‐Olesen . 2014. “DNA Methylation.” EcoSal Plus 6, no. 1: 1–35. 10.1128/ECOSALPLUS.ESP-0003-2013. PMC4231299 26442938

[crf370439-bib-0203] Maserati, A. , R. C. Fink , A. Lourenco , M. L. Julius , and F. Diez‐Gonzalez . 2017. “General Response of *Salmonella enterica* Serovar Typhimurium to Desiccation: A New Role for the Virulence Factors sopD and sseD in Survival.” PLoS ONE 12, no. 11: e0187692. 10.1371/JOURNAL.PONE.0187692. 29117268 PMC5678696

[crf370439-bib-0204] Maserati, A. , A. Lourenco , F. Diez‐Gonzalez , and R. C. Fink . 2018. “iTRAQ‐Based Global Proteomic Analysis of *Salmonella enterica* Serovar Typhimurium in Response to Desiccation, Low Water Activity, and Thermal Treatment.” Applied and Environmental Microbiology 84, no. 18: e00393‐18. 10.1128/AEM.00393-18. 29959250 PMC6121987

[crf370439-bib-0205] Mattick, K. L. , F. Jørgensen , J. D. Legan , et al. 2000. “Survival and Filamentation of *Salmonella enterica* Serovar Enteritidis PT4 and *Salmonella enterica* Serovar Typhimurium DT104 at Low Water Activity.” Applied and Environmental Microbiology 66, no. 4: 1274–1279. 10.1128/AEM.66.4.1274-1279.2000. 10742199 PMC91980

[crf370439-bib-0207] Mattick, K. L. , R. J. Rowbury , and T. J. Humphrey . 2003. “Morphological Changes to *Escherichia Coli* O157:H7, Commensal *E. coli* and *Salmonella* spp in Response to Marginal Growth Conditions, With Special Reference to Mildly Stressing Temperatures.” Science Progress 86, no. Pt 1–2: 103–113. 10.3184/003685003783238725. 12838606 PMC10368319

[crf370439-bib-0323] May, G. , E. Faatz , J. M. Lucht , M. Haardt , M. Bolliger , and E. Bremer . 1989. “Characterization of the Osmoregulated Escherichia coli proU Promoter and Identification of ProV as a Membrane‐associated Protein.” Molecular Microbiology 3, no. 11: 1521–1531. 10.1111/j.1365-2958.1989.tb00138.x. 2515417

[crf370439-bib-0208] Miao, J. , L. Chen , J. Wang , et al. 2017a. “Current Methodologies on Genotyping for Nosocomial Pathogen Methicillin‐Resistant *Staphylococcus aureus* (MRSA).” Microbial Pathogenesis 107: 17–28. 10.1016/J.MICPATH.2017.03.010. 28284852

[crf370439-bib-0209] Miao, J. , L. Chen , J. Wang , et al. 2017b. “Evaluation and Application of Molecular Genotyping on Nosocomial Pathogen‐Methicillin‐Resistant *Staphylococcus aureus* Isolates in Guangzhou Representative of Southern China.” Microbial Pathogenesis 107: 397–403. 10.1016/J.MICPATH.2017.04.016. 28414166

[crf370439-bib-0210] Miao, J. , W. Wang , W. Xu , et al. 2018. “The Fingerprint Mapping and Genotyping Systems Application on Methicillin‐Resistant *Staphylococcus aureus* .” Microbial Pathogenesis 125: 246–251. 10.1016/J.MICPATH.2018.09.031. 30243550

[crf370439-bib-0211] Michán, C. , M. Manchado , G. Dorado , and C. Pueyo . 1999. “In Vivo Transcription of the *Escherichia coli* oxyR Regulon as a Function of Growth Phase and in Response to Oxidative Stress.” Journal of Bacteriology 181, no. 9: 2759–2764. 10.1128/jb.181.9.2759-2764.1999. 10217765 PMC93716

[crf370439-bib-0212] Mizunoe, Y. , S. N. Wai , A. Takade , and S. I. Yoshida . 1999. “Restoration of Culturability of Starvation‐Stressed and Low‐Temperature‐Stressed *Escherichia coli* 0157 Cells by Using H_2_O_2_‐Degrading Compounds.” Archives of Microbiology 172, no. 1: 63–67. 10.1007/s002030050741. 10398754

[crf370439-bib-0213] Morishige, Y. , K. Fujimori , and F. Amano . 2013. “Differential Resuscitative Effect of Pyruvate and Its Analogues on VBNC (Viable But Non‐Culturable) *Salmonella* .” Microbes and Environments 28, no. 2: 180–186. 10.1264/JSME2.ME12174. 23595023 PMC4070669

[crf370439-bib-0214] Morishige, Y. , A. Koike , A. Tamura‐Ueyama , and F. Amano . 2017. “Induction of Viable but Nonculturable *Salmonella* in Exponentially Grown Cells by Exposure to a Low‐Humidity Environment and Their Resuscitation by Catalase.” Journal of Food Protection 80, no. 2: 288–294. 10.4315/0362-028X.JFP-16-183. 28221986

[crf370439-bib-0215] Mukamolova, G. V. , A. G. Murzin , E. G. Salina , et al. 2006. “Muralytic Activity of *Micrococcus luteus* Rpf and Its Relationship to Physiological Activity in Promoting Bacterial Growth and Resuscitation.” Molecular Microbiology 59, no. 1: 84–98. 10.1111/j.1365-2958.2005.04930.x. 16359320

[crf370439-bib-0216] Munro, P. M. , G. N. Flatau , R. L. Clement , and M. J. Gauthier . 1995. “Influence of the RpoS (KatF) Sigma Factor on Maintenance of Viability and Culturability of *Escherichia coli* and *Salmonella typhimurium* in Seawater.” Applied and Environmental Microbiology 61, no. 5: 1853–1858. 10.1128/aem.61.5.1853-1858.1995. 7646022 PMC167447

[crf370439-bib-0217] Mutz, Y. , S. da , D. K. A. Rosario , V. M. F. Paschoalin , and C. A. Conte‐Junior . 2020. “ *Salmonella enterica*: A Hidden Risk for Dry‐Cured Meat Consumption?” Critical Reviews in Food Science and Nutrition 60, no. 6: 976–990. 10.1080/10408398.2018.1555132. 30663891

[crf370439-bib-0218] Nascimento, M. d. S. d. , P. O. Pena , D. M. Brum , F. T. Imazaki , M. L. S. Tucci , and P. Efraim . 2013. “Behavior of *Salmonella* During Fermentation, Drying and Storage of Cocoa Beans.” International Journal of Food Microbiology 167, no. 3: 363–368. 10.1016/j.ijfoodmicro.2013.10.003. 24184616

[crf370439-bib-0219] Nascimento, M. S. , J. A. Carminati , K. N. Morishita , D. P. Amorim Neto , H. P. Pinheiro , and R. P. Maia . 2018. “Long‐Term Kinetics of *Salmonella typhimurium* ATCC 14028 Survival on Peanuts and Peanut Confectionery Products.” PLoS ONE 13, no. 2: e0192457. 10.1371/journal.pone.0192457. 29401480 PMC5798841

[crf370439-bib-0220] Nicolò, M. S. , A. Gioffrè , S. Carnazza , G. Platania , I. D. Silvestro , and S. P. P. Guglielmino . 2011. “Viable but Nonculturable State of Foodborne Pathogens in Grapefruit Juice: A Study of Laboratory.” Foodborne Pathogens and Disease 8, no. 1: 11–17. 10.1089/fpd.2009.0491. 20932087

[crf370439-bib-0221] Nievera, C. , J. J. C. Torgue , J. E. E. Grimwade , and A. C. Leonard . 2006. “SeqA Blocking of DnaA‐oriC Interactions Ensures Staged Assembly of the *E. coli* Pre‐RC.” Molecular Cell 24, no. 4: 581–592. 10.1016/J.MOLCEL.2006.09.016. 17114060 PMC1939805

[crf370439-bib-0222] Nikitushkin, V. D. , G. R. Demina , M. O. Shleeva , et al. 2015. “A Product of RpfB and RipA Joint Enzymatic Action Promotes the Resuscitation of Dormant Mycobacteria.” FEBS Journal 282, no. 13: 2500–2511. 10.1111/febs.13292. 25846449

[crf370439-bib-0223] Nikolaus, T. , J. Deiwick , C. Rappl , et al. 2001. “SseBCD Proteins Are Secreted by the Type III Secretion System of *Salmonella* Pathogenicity Island 2 and Function as a Translocon.” Journal of Bacteriology 183, no. 20: 6036–6045. 10.1128/jb.183.20.6036-6045.2001. 11567004 PMC99683

[crf370439-bib-0224] O'donnell‐Tormey, J. , C. F. Nathan , K. Lanks , C. J. Deboer , and J. De La Harpe . 1987. “Secretion of Pyruvate. An Antioxidant Defense of Mammalian Cells.” Journal of Experimental Medicine 165, no. 2: 500–514. 10.1084/JEM.165.2.500. 3102672 PMC2188509

[crf370439-bib-0225] Oku, K. , H. Watanabe , M. Kubota , et al. 2003. “NMR and Quantum Chemical Study on the OH⋯π and CH⋯O Interactions Between Trehalose and Unsaturated Fatty Acids: Implication for the Mechanism of Antioxidant Function of Trehalose.” Journal of the American Chemical Society 125, no. 42: 12739–12748. 10.1021/ja034777e. 14558821

[crf370439-bib-0226] Oliveira, M. M. , F. A. de Almeida , F. Baglinière , L. L. de Oliveira , and M. C. D. Vanetti . 2021. “Behavior of *Salmonella* Enteritidis and *Shigella flexneri* During Induction and Recovery of the Viable but Nonculturable State.” FEMS Microbiology Letters 368, no. 14: 87. 10.1093/FEMSLE/FNAB087. 34227668

[crf370439-bib-0227] Oliver, J. D. 2005. “The Viable but Nonculturable State in Bacteria.” Journal of Microbiology 43, no. spc1: 93–100. 15765062

[crf370439-bib-0228] Oliver, J. D. , and R. Bockian . 1995. “In Vivo Resuscitation, and Virulence Towards Mice, of Viable but Nonculturable Cells of Vibrio Vulnificus.” Applied and Environmental Microbiology 61, no. 7: 2620–2623. 10.1128/aem.61.7.2620-2623.1995. 7618873 PMC167533

[crf370439-bib-0229] Ophir, T. , and D. L. Gutnick . 1994. “A Role for Exopolysaccharides in the Protection of Microorganisms From Desiccation.” Applied and Environmental Microbiology 60, no. 2: 740–745. 10.1128/aem.60.2.740-745.1994. 16349202 PMC201377

[crf370439-bib-0230] Osborne, S. E. , and B. K. Coombes . 2011. “Transcriptional Priming of *Salmonella* Pathogenicity Island‐2 Precedes Cellular Invasion.” PLoS ONE 6, no. 6: e21648. 10.1371/JOURNAL.PONE.0021648. 21738750 PMC3125303

[crf370439-bib-0231] Palmer, L. D. , M. D. Paxhia , and D. M. Downs . 2015. “Induction of the Sugar‐Phosphate Stress Response Allows Saccharomyces Cerevisiae 2‐Methyl‐4‐Amino‐5‐Hydroxymethylpyrimidine Phosphate Synthase to Function in *Salmonella enterica* .” Journal of Bacteriology 197, no. 22: 3554–3562. 10.1128/jb.00576-15. 26324451 PMC4621092

[crf370439-bib-0232] Pan, H. , and Q. Ren . 2022. “Wake Up! Resuscitation of Viable but Nonculturable Bacteria: Mechanism and Potential Application.” Foods 12, no. 1: 82. 10.3390/FOODS12010082. 36613298 PMC9818539

[crf370439-bib-0233] Panutdaporn, N. , K. Kawamoto , H. Asakura , and S. I. Makino . 2006. “Resuscitation of the Viable but Non‐Culturable State of *Salmonella enterica* Serovar Oranienburg by Recombinant Resuscitation‐Promoting Factor Derived From *Salmonella typhimurium* Strain LT2.” International Journal of Food Microbiology 106, no. 3: 241–247. 10.1016/J.IJFOODMICRO.2005.06.022. 16213054

[crf370439-bib-0234] Paterson, G. K. , D. B. Cone , H. Northen , S. E. Peters , and D. J. Maskell . 2009. “Deletion of the Gene Encoding the Glycolytic Enzyme Triosephosphate Isomerase (tpi) Alters Morphology of *Salmonella enterica* Serovar Typhimurium and Decreases Fitness in Mice.” FEMS Microbiology Letters 294, no. 1: 45–51. 10.1111/J.1574-6968.2009.01553.X. 19493007

[crf370439-bib-0316] Pereira, A. , F. Prestes , and A. Silva , and Nascimento . 2020. “Evaluation of the Thermal Resistance of Salmonella Typhimurium ATCC 14028 After Long‐term Blanched Peanut Kernel Storage.” LWT 117: 108701. 10.1016/j.lwt.2019.108701.

[crf370439-bib-0235] PHAC . 2014. Public Health Notice: Outbreak of Salmonella Infections Related to Sprouted Chia Seed Powder . Public Health Agency of Canada. https://www.canada.ca/en/public‐health/services/public‐health‐notices/2014/public‐health‐notice‐outbreak‐salmonella‐infections‐related‐sprouted‐chia‐seed‐powder.html.

[crf370439-bib-0236] PHAC . 2019. Public Health Notice—Outbreak of Salmonella Infections—Canada.ca . Public Health Agency of Canada. https://www.canada.ca/en/public‐health/services/public‐health‐notices/2019/outbreak‐salmonella.html.

[crf370439-bib-0237] Phillips, L. E. , T. J. Humphrey , and H. M. Lappin‐Scott . 1998. “Chilling Invokes Different Morphologies in Two *Salmonella* Enteritidis PT4 Strains.” Journal of Applied Microbiology 84, no. 5: 820–826. 10.1046/J.1365-2672.1998.00417.X. 9674136

[crf370439-bib-0238] Pienaar, J. A. , A. Singh , and T. G. Barnard . 2016. “The Viable but Non‐Culturable State in Pathogenic *Escherichia coli*: A General Review.” African Journal of Laboratory Medicine 5, no. 1: a368. 10.4102/AJLM.V5I1.368. PMC5436400 28879110

[crf370439-bib-0239] Pilonieta, M. C. , T. A. Nagy , D. R. Jorgensen , and C. S. Detweiler . 2012. “A Glycine Betaine Importer Limits *Salmonella* Stress Resistance and Tissue Colonization by Reducing Trehalose Production.” Molecular Microbiology 84, no. 2: 296–309. 10.1111/J.1365-2958.2012.08022.X. 22375627 PMC3323685

[crf370439-bib-0320] Poolman, B. , and E. Glaasker . 1998. “Regulation of Compatible Solute Accumulation in Bacteria.” Molecular Microbiology 29, no. 2: 397–407. 10.1046/j.1365-2958.1998.00875.x. 9720860

[crf370439-bib-0240] Poolman, B. , P. Blount , J. H. A. Folgering , R. H. E. Friesen , P. C. Moe , and T. Van Der Heide . 2002. “How Do Membrane Proteins Sense Water Stress?” Molecular Microbiology 44, no. 4: 889–902. 10.1046/J.1365-2958.2002.02894.X. 12010487

[crf370439-bib-0241] Postnikova, O. A. , J. Shao , N. M. Mock , C. J. Baker , and L. G. Nemchinov . 2015. “Gene Expression Profiling in Viable but Nonculturable (VBNC) Cells of *Pseudomonas syringae* Pv. Syringae.” Frontiers in Microbiology 6: 1419. 10.3389/fmicb.2015.01419. 26733964 PMC4683178

[crf370439-bib-0242] Potts, A. H. , Y. Guo , B. M. M. Ahmer , and T. Romeo . 2019. “Role of CsrA in Stress Responses and Metabolism Important for *Salmonella* Virulence Revealed by Integrated Transcriptomics.” PLoS ONE 14, no. 1: e0211430. 10.1371/JOURNAL.PONE.0211430. 30682134 PMC6347204

[crf370439-bib-0243] Potts, M. 1994. “Desiccation Tolerance of Prokaryotes.” Microbiological Reviews 58, no. 4: 755–805. 10.1128/MR.58.4.755-805.1994. 7854254 PMC372989

[crf370439-bib-0244] Quinn, H. J. , A. D. S. Cameron , and C. J. Dorman . 2014. “Bacterial Regulon Evolution: Distinct Responses and Roles for the Identical OmpR Proteins of *Salmonella typhimurium* and *Escherichia coli* in the Acid Stress Response.” PLOS Genetics 10, no. 3: e1004215. 10.1371/JOURNAL.PGEN.1004215. 24603618 PMC3945435

[crf370439-bib-0245] Quirós, C. , M. Herrero , L. A. Garcáa , and M. Dáaz . 2009. “Quantitative Approach to Determining the Contribution of Viable‐But‐Nonculturable Subpopulations to Malolactic Fermentation Processes.” Applied and Environmental Microbiology 75, no. 9: 2977–2981. 10.1128/AEM.01707-08. 19270138 PMC2681708

[crf370439-bib-0246] Raffatellu, M. , Y. H. Sun , R. P. Wilson , et al. 2005. “Host Restriction of *Salmonella enterica* Serotype Typhi Is Not Caused by Functional Alteration of SipA, SopB, or SopB.” Infection and Immunity 73, no. 12: 7817–7826. 10.1128/iai.73.12.7817-7826.2005. 16299271 PMC1307101

[crf370439-bib-0247] Reissbrodt, R. , I. Rienaecker , J. M. Romanova , et al. 2002. “Resuscitation of *Salmonella enterica* Serovar Typhimurium and Enterohemorrhagic *Escherichia coli* From the Viable but Nonculturable State by Heat‐Stable Enterobacterial Autoinducer.” Applied and Environmental Microbiology 68, no. 10: 4788–4794. 10.1128/AEM.68.10.4788-4794.2002. 12324321 PMC126406

[crf370439-bib-0248] Rensing, C. , B. Fan , R. Sharma , B. Mitra , and B. P. Rosen . 2000. “CopA: An *Escherichia coli* Cu(I)‐Translocating P‐Type ATPase.” Proceedings of the National Academy of Sciences of the United States of America 97, no. 2: 652–656. 10.1073/pnas.97.2.652. 10639134 PMC15385

[crf370439-bib-0249] Rensing, C. , B. Mitra , and B. P. Rosen . 1997. “The zntA Gene of *Escherichia coli* Encodes a Zn(II)‐Translocating P‐Type ATPase.” Proceedings of the National Academy of Sciences of the United States of America 94, no. 26: 14326–14331. 10.1073/pnas.94.26.14326. 9405611 PMC24962

[crf370439-bib-0250] Rhodius, V. A. , W. C. Suh , G. Nonaka , J. West , and C. A. Gross . 2005. “Conserved and Variable Functions of the σE Stress Response in Related Genomes.” PLoS Biology 4, no. 1: e2. 10.1371/JOURNAL.PBIO.0040002. PMC1312014 16336047

[crf370439-bib-0251] Roberson, E. B. , and M. K. Firestone . 1992. “Relationship Between Desiccation and Exopolysaccharide Production in a Soil *Pseudomonas* sp.” Applied and Environmental Microbiology 58, no. 4: 1284–1291. 10.1128/AEM.58.4.1284-1291.1992. 16348695 PMC195588

[crf370439-bib-0252] Rodrigues, R. C. , E. Martins , M. C. D. Vanetti , U. M. Pinto , and M. T. dos Santos . 2015. “Induction of the Viable but Nonculturable State of *Salmonella enterica* Serovar Enteritidis Deficient in (p)ppGpp Synthesis.” Annals of Microbiology 65, no. 4: 2171–2178. 10.1007/s13213-015-1057-6.

[crf370439-bib-0253] Roeßler, M. , and V. Müller . 2001. “Osmoadaptation in Bacteria and Archaea: Common Principles and Differences.” Environmental Microbiology 3, no. 12: 743–754. 10.1046/j.1462-2920.2001.00252.x. 11846768

[crf370439-bib-0254] Romero‐González, L. E. , D. Pérez‐Morales , D. Cortés‐Avalos , et al. 2020. “The *Salmonella typhimurium* InvF‐SicA Complex Is Necessary for the Transcription of sopB in the Absence of the Repressor H‐NS.” PLoS ONE 15, no. 10: e0240617. 10.1371/JOURNAL.PONE.0240617. 33119619 PMC7595419

[crf370439-bib-0255] Roszak, D. B. , D. J. Grimes , and R. R. Colwell . 1984. “Viable but Nonrecoverable Stage of *Salmonella* Enteritidis in Aquatic Systems.” Canadian Journal of Microbiology 30, no. 3: 334–338. 10.1139/m84-049. 6372975

[crf370439-bib-0256] Rothfield, L. , S. Justice , and J. García‐Lara . 1999. “Bacterial Cell Division.” Annual Review of Genetics 33: 423–448. 10.1146/annurev.genet.33.1.423. 10690414

[crf370439-bib-0257] Rowley, G. , M. Spector , J. Kormanec , and M. Roberts . 2006. “Pushing the Envelope: Extracytoplasmic Stress Responses in Bacterial Pathogens.” Nature Reviews Microbiology 4, no. 5: 383–394. 10.1038/nrmicro1394. 16715050

[crf370439-bib-0258] Rui, B. , T. Shen , H. Zhou , et al. 2010. “A Systematic Investigation of *Escherichia coli* Central Carbon Metabolism in Response to Superoxide Stress.” BMC Systems Biology 4, no. 1: 122. 10.1186/1752-0509-4-122. 20809933 PMC2944137

[crf370439-bib-0259] Rychlik, I. , and P. A. Barrow . 2005. “ *Salmonella* Stress Management and Its Relevance to Behaviour During Intestinal Colonisation and Infection.” FEMS Microbiology Reviews 29, no. 5: 1021–1040. 10.1016/J.FEMSRE.2005.03.005. 16023758

[crf370439-bib-0260] Rychlik, I. , D. Gregorova , and H. Hradecka . 2006. “Distribution and Function of Plasmids in *Salmonella enterica* .” Veterinary Microbiology 112, no. 1: 1–10. 10.1016/J.VETMIC.2005.10.030. 16303262

[crf370439-bib-0261] Sakoh, M. , K. Ito , and Y. Akiyama . 2005. “Proteolytic Activity of HtpX, a Membrane‐Bound and Stress‐Controlled Protease From *Escherichia coli* .” Journal of Biological Chemistry 280, no. 39: 33305–33310. 10.1074/JBC.M506180200. 16076848

[crf370439-bib-0262] Salive, A. F. V. , C. V. Prudêncio , F. Baglinière , L. L. Oliveira , S. O. Ferreira , and M. C. D. Vanetti . 2020. “Comparison of Stress Conditions to Induce Viable but Non‐Cultivable State in *Salmonella* .” Brazilian Journal of Microbiology 51, no. 3: 1269–1277. 10.1007/s42770-020-00261-w. 32291740 PMC7455614

[crf370439-bib-0263] Schnaitman, C. A. , and J. D. Klenat . 1993. “Genetics of Lipopolysaccharide Biosynthesis in Enteric Bacteria.” Microbiological Reviews 57, no. 3: 655–682. 10.1128/MR.57.3.655-682.1993. 7504166 PMC372930

[crf370439-bib-0264] Senoh, M. , J. Ghosh‐Banerjee , T. Ramamurthy , et al. 2010. “Conversion of Viable but Nonculturable Vibrio Cholerae to the Culturable State by Co‐Culture With Eukaryotic Cells.” Microbiology and Immunology 54, no. 9: 502–507. 10.1111/j.1348-0421.2010.00245.x. 20840148

[crf370439-bib-0265] Shen, S. , and F. C. Fang . 2012. “Integrated Stress Responses in *Salmonella* .” International Journal of Food Microbiology 152, no. 3: 75–81. 10.1016/J.IJFOODMICRO.2011.04.017. 21570144 PMC3164900

[crf370439-bib-0266] Sheridan, G. E. C. , C. I. Masters , J. A. Shallcross , and B. M. Mackey . 1998. “Detection of mRNA by Reverse Transcription‐PCR as Indicator of Viability in *Escherichia coli* Cells.” Applied and Environmental Microbiology 64, no. 4: 1313–1318. 10.1128/AEM.64.4.1313-1318.1998. 9546166 PMC106147

[crf370439-bib-0267] Shimohata, N. , S. Chiba , N. Saikawa , K. Ito , and Y. Akiyama . 2002. “The Cpx Stress Response System of *Escherichia coli* Senses Plasma Membrane Proteins and Controls HtpX, a Membrane Protease With a Cytosolic Active Site.” Genes to Cells 7, no. 7: 653–662. 10.1046/J.1365-2443.2002.00554.X. 12081643

[crf370439-bib-0268] Solano, C. , B. García , J. Valle , et al. 2002. “Genetic Analysis of *Salmonella* Enteritidis Biofilm Formation: Critical Role of Cellulose.” Molecular Microbiology 43, no. 3: 793–808. 10.1046/j.1365-2958.2002.02802.x. 11929533

[crf370439-bib-0269] Song, H. , and S. Y. Lee . 2021. “High Concentration of Sodium Chloride Could Induce the Viable and Culturable States of *Escherichia coli* O157:H7 and *Salmonella enterica* Serovar Enteritidis.” Letters in Applied Microbiology 72, no. 6: 741–749. 10.1111/LAM.13468. 33650683

[crf370439-bib-0270] Stenberg, F. , P. Chovanec , S. L. Maslen , et al. 2005. “Protein Complexes of the *Escherichia coli* Cell Envelope.” Journal of Biological Chemistry 280, no. 41: 34409–34419. 10.1074/JBC.M506479200. 16079137

[crf370439-bib-0271] Stirling, D. A. , C. S. J. Hulton , L. Waddell , et al. 1989. “Molecular Characterization of the proU Loci of *Salmonella typhimurium* and *Escherichia coli* Encoding Osmoregulated Glycine Betaine Transport Systems.” Molecular Microbiology 3, no. 8: 1025–1038. 10.1111/j.1365-2958.1989.tb00253.x. 2691838

[crf370439-bib-0272] Strom, A. R. , and I. Kaasen . 1993. “Trehalose Metabolism in *Escherichia coli*: Stress Protection and Stress Regulation of Gene Expression.” Molecular Microbiology 8, no. 2: 205–210. 10.1111/j.1365-2958.1993.tb01564.x. 8391102

[crf370439-bib-0273] Sun, S. , Y. Xie , X. Zhou , M. J. Zhu , S. Sablani , and J. Tang . 2023. “Survival and Thermal Resistance of *Salmonella* in Chocolate Products With Different Water Activities.” Food Research International 172: 113209. 10.1016/J.FOODRES.2023.113209. 37689954

[crf370439-bib-0274] Tanaka, M. , A. M. Earl , H. A. Howell , et al. 2004. “Analysis of *Deinococcus radiodurans*'s Transcriptional Response to Ionizing Radiation and Desiccation Reveals Novel Proteins That Contribute to Extreme Radioresistance.” Genetics 168, no. 1: 21–33. 10.1534/GENETICS.104.029249. 15454524 PMC1448114

[crf370439-bib-0275] Teunis, P. F. M. , F. Kasuga , A. Fazil , I. D. Ogden , O. Rotariu , and N. J. C. Strachan . 2010. “Dose–Response Modeling of *Salmonella* Using Outbreak Data.” International Journal of Food Microbiology 144, no. 2: 243–249. 10.1016/J.IJFOODMICRO.2010.09.026. 21036411

[crf370439-bib-0276] Thompson, L. J. , D. S. Merrell , B. A. Neilan , H. Mitchell , A. Lee , and S. Falkow . 2003. “Gene Expression Profiling of *Helicobacter pylori* Reveals a Growth‐Phase‐Dependent Switch in Virulence Gene Expression.” Infection and Immunity 71, no. 5: 2643–2655. 10.1128/iai.71.5.2643-2655.2003. 12704139 PMC153220

[crf370439-bib-0277] Thomsen, L. E. , M. S. Chadfield , J. Bispham , T. S. Wallis , J. E. Olsen , and H. Ingmer . 2003. “Reduced Amounts of LPS Affect Both Stress Tolerance and Virulence of *Salmonella enterica* Serovar Dublin.” FEMS Microbiology Letters 228, no. 2: 225–231. 10.1016/S0378-1097(03)00762-6. 14638428

[crf370439-bib-0278] Toguchi, A. , M. Siano , M. Burkart , and R. M. Harshey . 2000. “Genetics of Swarming Motility in *Salmonella enterica* Serovar Typhimurium: Critical Role for Lipopolysaccharide.” Journal of Bacteriology 182, no. 22: 6308–6321. 10.1128/jb.182.22.6308-6321.2000. 11053374 PMC94776

[crf370439-bib-0279] Van den Ent, F. , L. A. Amos , and J. Löwe . 2001. “Prokaryotic Origin of the Actin Cytoskeleton.” Nature 413, no. 6851: 39–44. 10.1038/35092500. 11544518

[crf370439-bib-0280] Varma, S. D. , K. Hegde , and M. Henein . 2003. “Oxidative Damage to Mouse Lens in Culture. Protective Effect of Pyruvate.” Biochimica et Biophysica Acta (BBA)—General Subjects 1621, no. 3: 246–252. 10.1016/S0304-4165(03)00075-8. 12787921

[crf370439-bib-0281] Vecchietti, D. , S. Ferrara , R. Rusmini , R. Macchi , M. Milani , and G. Bertoni . 2016. “Crystal Structure of YeaZ From *Pseudomonas aeruginosa* .” Biochemical and Biophysical Research Communications 470, no. 2: 460–465. 10.1016/J.BBRC.2016.01.008. 26768361

[crf370439-bib-0282] Villa‐Rojas, R. , M. J. Zhu , N. C. Paul , et al. 2017. “Biofilm Forming *Salmonella* Strains Exhibit Enhanced Thermal Resistance in Wheat Flour.” Food Control 73: 689–695. 10.1016/J.FOODCONT.2016.09.021.

[crf370439-bib-0283] von Hertwig, A. M. , F. S. Prestes , and M. S. Nascimento . 2023. “Comparative Evaluation of the Effectiveness of Alcohol‐Based Sanitizers, UV‐C Radiation and Hot Air on Three‐Age *Salmonella* Biofilms.” Food Microbiology 113: 104278. 10.1016/J.FM.2023.104278. 37098425

[crf370439-bib-0284] Waldminghaus, T. , C. Weigel , and K. Skarstad . 2012. “Replication Fork Movement and Methylation Govern SeqA Binding to the *Escherichia coli* Chromosome.” Nucleic Acids Research 40, no. 12: 5465–5476. 10.1093/NAR/GKS187. 22373925 PMC3384311

[crf370439-bib-0285] Walthers, D. , R. K. Carroll , W. W. Navarre , S. J. Libby , F. C. Fang , and L. J. Kenney . 2007. “The Response Regulator SsrB Activates Expression of Diverse *Salmonella* Pathogenicity Island 2 Promoters and Counters Silencing by the Nucleoid‐Associated Protein H‐NS.” Molecular Microbiology 65, no. 2: 477–493. 10.1111/j.1365-2958.2007.05800.x. 17630976

[crf370439-bib-0286] Walthers, D. , Y. Li , Y. Liu , G. Anand , J. Yan , and L. J. Kenney . 2011. “ *Salmonella enterica* Response Regulator SsrB Relieves H‐NS Silencing by Displacing H‐NS Bound in Polymerization Mode and Directly Activates Transcription.” Journal of Biological Chemistry 286, no. 3: 1895–1902. 10.1074/JBC.M110.164962. 21059643 PMC3023485

[crf370439-bib-0287] Wang, L. , Z. Xihong , J. Chu , et al. 2011. “Application of an Improved Loop‐Mediated Isothermal Amplification Detection of *Vibrio parahaemolyticus* From Various Seafood Samples.” African Journal of Microbiology Research 5, no. 31. 10.5897/AJMR11.1237.

[crf370439-bib-0288] Wang, L. C. , L. K. Morgan , P. Godakumbura , L. J. Kenney , and G. S. Anand . 2012. “The Inner Membrane Histidine Kinase EnvZ Senses Osmolality via Helix‐Coil Transitions in the Cytoplasm.” EMBO Journal 31, no. 11: 2648–2659. 10.1038/emboj.2012.99. 22543870 PMC3365433

[crf370439-bib-0289] Wang, X. , X. Xu , W. Hu , et al. 2019. “Visual Detection of Porcine Epidemic Diarrhea Virus Using a Novel Reverse Transcription Polymerase Spiral Reaction Method.” BMC Veterinary Research 15, no. 1: 1–7. 10.1186/s12917-019-1851-7. 30987635 PMC6466714

[crf370439-bib-0290] Waters, C. M. , and B. L. Bassler . 2005. “Quorum Sensing: Cell‐to‐Cell Communication in Bacteria.” Annual Review of Cell and Developmental Biology 21: 319–346. 10.1146/annurev.cellbio.21.012704.131001. 16212498

[crf370439-bib-0291] White, A. P. , D. L. Gibson , G. A. Grassl , et al. 2008. “Aggregation via the Red, Dry, and Rough Morphotype Is Not a Virulence Adaptation in *Salmonella enterica* Serovar Typhimurium.” Infection and Immunity 76, no. 3: 1048–1058. 10.1128/iai.01383-07. 18195033 PMC2258808

[crf370439-bib-0292] White, A. P. , D. L. Gibson , W. Kim , W. W. Kay , and M. G. Surette . 2006. “Thin Aggregative Fimbriae and Cellulose Enhance Long‐Term Survival and Persistence of *Salmonella* .” Journal of Bacteriology 188, no. 9: 3219–3227. 10.1128/jb.188.9.3219-3227.2006. 16621814 PMC1447457

[crf370439-bib-0293] White, D. , J. Drummond , and C. Fuqua . 2012. The Physiology and Biochemistry of Prokaryotes. Oxford University Press.

[crf370439-bib-0294] Whitham, H. K. , P. Sundararaman , D. Dewey‐Mattia , et al. 2021. “Novel Outbreak‐Associated Food Vehicles, United States.” Emerging Infectious Diseases 27, no. 10: 2554–2559. 10.3201/eid2710.204080. 34545783 PMC8462308

[crf370439-bib-0295] Wong, H. C. , and S. H. Liu . 2008. “Characterization of the Low‐Salinity Stress in Vibrio Vulnificus.” Journal of Food Protection 71, no. 2: 416–419. 10.4315/0362-028X-71.2.416. 18326198

[crf370439-bib-0296] Woo, Y. J. , M. D. Taylor , J. E. Cohen , et al. 2004. “Ethyl Pyruvate Preserves Cardiac Function and Attenuates Oxidative Injury After Prolonged Myocardial Ischemia.” Journal of Thoracic and Cardiovascular Surgery 127, no. 5: 1262–1269. 10.1016/J.JTCVS.2003.11.032. 15115981

[crf370439-bib-0297] Wood, J. M. 1999. “Osmosensing by Bacteria: Signals and Membrane‐Based Sensors.” Microbiology and Molecular Biology Reviews 63, no. 1: 230–262. 10.1128/mmbr.63.1.230-262.1999. 10066837 PMC98963

[crf370439-bib-0298] Wood, J. M. , E. Bremer , L. N. Csonka , et al. 2001. “Osmosensing and Osmoregulatory Compatible Solute Accumulation by Bacteria.” Comparative Biochemistry and Physiology Part A: Molecular & Integrative Physiology 130, no. 3: 437–460. 10.1016/S1095-6433(01)00442-1. 11913457

[crf370439-bib-0299] Wu, T. , J. Malinverni , N. Ruiz , S. Kim , T. J. Silhavy , and D. Kahne . 2005. “Identification of a Multicomponent Complex Required for Outer Membrane Biogenesis in *Escherichia coli* .” Cell 121, no. 2: 235–245. 10.1016/J.CELL.2005.02.015. 15851030

[crf370439-bib-0300] Xu, J. , D. H. Shah , J. Song , and J. Tang . 2020. “Changes in Cellular Structure of Heat‐Treated *Salmonella* in Low‐Moisture Environments.” Journal of Applied Microbiology 129, no. 2: 434–442. 10.1111/JAM.14614. 32052556

[crf370439-bib-0301] Xu, J. , J. Tang , Y. Jin , et al. 2019. “High Temperature Water Activity as a Key Factor Influencing Survival of *Salmonella* Enteritidis PT30 in Thermal Processing.” Food Control 98: 520–528. 10.1016/J.FOODCONT.2018.11.054.

[crf370439-bib-0302] Yamazoe, M. , S. Adachi , S. Kanaya , K. Ohsumi , and S. Hiraga . 2005. “Sequential Binding of SeqA Protein to Nascent DNA Segments at Replication Forks in Synchronized Cultures of *Escherichia coli* .” Molecular Microbiology 55, no. 1: 289–298. 10.1111/j.1365-2958.2004.04389.x. 15612935

[crf370439-bib-0303] Yim, H. H. , and M. Villarejo . 1992. “osmY, a New Hyperosmotically Inducible Gene, Encodes a Periplasmic Protein in *Escherichia coli* .” Journal of Bacteriology 174, no. 11: 3637–3644. 10.1128/JB.174.11.3637-3644.1992. 1317380 PMC206052

[crf370439-bib-0304] Yin, C. , D. He , S. Chen , et al. 2016. “Exogenous Pyruvate Facilitates Cancer Cell Adaptation to Hypoxia by Serving as an Oxygen Surrogate.” Oncotarget 7, no. 30: 47494–47510. 10.18632/ONCOTARGET.10202. 27374086 PMC5216956

[crf370439-bib-0305] Zeng, B. , G. Zhao , X. Cao , Z. Yang , C. Wang , and L. Hou . 2013. “Formation and Resuscitation of Viable but Nonculturable *Salmonella* Typhi.” BioMed Research International 2013, no. 1: 907170. 10.1155/2013/907170. 23509799 PMC3591152

[crf370439-bib-0306] Zhang, S. , R. L. Santos , R. M. Tsolis , et al. 2002. “The *Salmonella enterica* Serotype Typhimurium Effector Proteins SipA, SopA, SopB, SopD, and SopE2 Act in Concert to Induce Diarrhea in Calves.” Infection and Immunity 70, no. 7: 3843–3855. 10.1128/iai.70.7.3843-3855.2002. 12065528 PMC128071

[crf370439-bib-0307] Zhang, Y. , X. Liao , T. Ding , and J. Feng . 2024. “Tolerance Variations and Mechanisms of *Salmonella enterica* Serovar Newport in Response to Long‐Term Hypertonic Stress.” Food Quality and Safety 8: 1–13. 10.1093/FQSAFE/FYAD068.

[crf370439-bib-0308] Zhang, Y. M. , and C. O. Rock . 2008. “Membrane Lipid Homeostasis in Bacteria.” Nature Reviews Microbiology 6, no. 3: 222–233. 10.1038/nrmicro1839. 18264115

[crf370439-bib-0309] Zhao, F. , X. Bi , Y. Hao , and X. Liao . 2013. “Induction of Viable but Nonculturable *Escherichia coli* O157:H7 by High Pressure CO_2_ and Its Characteristics.” PLoS ONE 8, no. 4: e62388. 10.1371/journal.pone.0062388. 23626816 PMC3633907

[crf370439-bib-0310] Zhao, F. , Y. Wang , H. An , Y. Hao , X. Hu , and X. Liao . 2016. “New Insights Into the Formation of Viable but Nonculturable *Escherichia coli* O157:H7 Induced by High‐Pressure CO_2_ .” MBio 7, no. 4: 961–977. 10.1128/mBio.00961-16. PMC4999544 27578754

[crf370439-bib-0311] Zhao, G. , P. Ceci , A. Ilari , et al. 2002. “Iron and Hydrogen Peroxide Detoxification Properties of DNA‐Binding Protein From Starved Cells: A FERRITIN‐LIKE DNA‐BINDING PROTEIN of *Escherichia coli* .” Journal of Biological Chemistry 277, no. 31: 27689–27696. 10.1074/JBC.M202094200. 12016214

[crf370439-bib-0312] Zhao, X. , J. Zhong , C. Wei , C.‐W. Lin , and T. Ding . 2017. “Current Perspectives on Viable but Non‐Culturable State in Foodborne Pathogens.” Frontiers in Microbiology 8: 580. 10.3389/fmicb.2017.00580. 28421064 PMC5378802

[crf370439-bib-0313] Zhou, D. , M. S. Mooseker , and J. E. Galán . 1999. “Role of the *S. typhimurium* Actin‐Binding Protein SipA in Bacterial Internalization.” Science 283, no. 5410: 2092–2095. 10.1126/science.283.5410.209. 10092234

[crf370439-bib-0314] Zogaj, X. , M. Nimtz , M. Rohde , W. Bokranz , and U. Römling . 2001. “The Multicellular Morphotypes of *Salmonella typhimurium* and *Escherichia coli* Produce Cellulose as the Second Component of the Extracellular Matrix.” Molecular Microbiology 39, no. 6: 1452–1463. 10.1046/J.1365-2958.2001.02337.X. 11260463

